# Immunogenicity, safety, and efficacy of the HPV vaccines among people living with HIV: A systematic review and meta-analysis

**DOI:** 10.1016/j.eclinm.2022.101585

**Published:** 2022-08-03

**Authors:** Lisa Staadegaard, Minttu M. Rönn, Nirali Soni, Meghan E. Bellerose, Paul Bloem, Marc Brisson, Mathieu Maheu-Giroux, Ruanne V. Barnabas, Melanie Drolet, Philippe Mayaud, Shona Dalal, Marie-Claude Boily

**Affiliations:** a Department of Infectious Diseases Epidemiology, Imperial College, London, United Kingdom; b Department of Global Health and Population, Harvard T.H. Chan School of Public Health, Boston, United States; c Medical Research Council Centre for Global Infectious Disease Analysis, School of Public Health, Imperial College London, London, United Kingdom; d World Health Organization, Geneva, Switzerland; e Laval University, Québec, Canada; f Centre de recherche du CHU de Québec-Université Laval, Canada; g Department of Epidemiology and Biostatistics, School of Population and Global Health, McGill University, Montreal, Canada; h Department of Global Health, Division of Allergy and Infectious Diseases, University of Washington, Seattle, WA, United States; i Faculty of Infectious & Tropical Diseases, London School of Hygiene and Tropical Medicine, London, United Kingdom

**Keywords:** Human papilloma virus, HIV, Cervical cancer, Vaccination, Meta-analysis

## Abstract

**Background:**

Vaccines have been demonstrated to protect against high-risk human papillomavirus infection (HPV), including HPV-16/18, and cervical lesions among HIV negative women. However, their efficacy remains uncertain for people living with HIV (PLHIV).We systematically reviewed available evidence on HPV vaccine on immunological, virological, or other biological outcomes in PLHIV.

**Methods:**

We searched five electronic databases (PubMed, Medline and Embase, clinicaltrials.gov and the WHO clinical trial database) for longitudinal prospective studies reporting immunogenicity, virological, cytological, histological, clinical or safety endpoints following prophylactic HPV vaccination among PLHIV. We included studies published by February 11th, 2021. We summarized results, assessed study quality, and conducted meta-analysis and subgroup analyses, where possible.

**Findings:**

We identified 43 publications stemming from 18 independent studies (*N_s_*=18), evaluating the quadrivalent (*N_s_*=15), bivalent (*N_s_*=4) and nonavalent (*N_s_*=1) vaccines. A high proportion seroconverted for the HPV vaccine types. Pooled proportion seropositive by 28 weeks following 3 doses with the bivalent, quadrivalent, and nonavalent vaccines were 0.99 (95% confidence interval: 0.95-1.00, *N_s_*=1), 0.99 (0.98-1.00, *N_s_*=9), and 1.00 (0.99-1.00, *N_s_*=1) for HPV-16 and 0.99 (0.96-1.00, *N_s_*=1), 0.94 (0.91-0.96, *N_s_*=9), and 1.00 (0.99-1.00, *N_s_*=1) for HPV-18, respectively. Seropositivity remained high among people who received 3 doses despite some declines in antibody titers and lower seropositivity over time, especially for HPV-18, for the quadrivalent than the bivalent vaccine, and for HIV positive than negative individuals. Seropositivity for HPV-18 at 29–99 weeks among PLHIV was 0.72 (0.66-0.79, *N_s_*=8) and 0.96 (0.92-0.99, *N_s_*=2) after 3 doses of the quadrivalent and bivalent vaccine, respectively and 0.94 (0.90-0.98, *N_s_*=3) among HIV-negative historical controls. Evidence suggests that the seropositivity after vaccination declines over time but it can lasts at least 2–4 years. The vaccines were deemed safe among PLHIV with few serious adverse events. Evidence of HPV vaccine efficacy against acquisition of HPV infection and/or associated disease from the eight trials available was inconclusive due to the low quality.

**Interpretation:**

PLHIV have a robust and safe immune response to HPV vaccination. Antibody titers and seropositivity rates decline over time but remain high. The lack of a formal correlate of protection and efficacy results preclude definitive conclusions on the clinical benefits. Nevertheless, given the burden of HPV disease in PLHIV, although the protection may be shorter or less robust against HPV-18, the robust immune response suggests that PLHIV may benefit from receiving HPV vaccination after acquiring HIV. Better quality studies are needed to demonstrate the clinical efficacy among PLHIV.

**Funding:**

World Health Organization. MRC Centre for Global Infectious Disease Analysis, Canadian Institutes of Health Research, UK Medical Research Council (MRC).

**Research in context**

**Evidence before this study**

There are four licensed HPV vaccines, which are effective in preventing cervical cancer caused by the most common high-risk HPV infections. Cervical cancer incidence in women living with HIV is 6 times higher than for women without HIV, but there remains uncertainty regarding the vaccine effectiveness among people with HIV. We searched PubMed, Medline and Embase for peer-reviewed articles without language restrictions. We used terms for HIV, HPV, HPV-associated disease, and HPV vaccines, and included studies published up to February 11^th^, 2021. We identified five systematic reviews including two reviews with quantitative summaries of data. The studies summarized evidence from up to seven randomised controlled trials (RCTs). The reviews suggested the HPV vaccines were safe and immunogenic across different populations. Despite highlighting heterogeneity across studies, none of the reviews evaluated the influence of vaccine type, vaccine dose, timing of measurement, or baseline serostatus.

**Added value of this study**

We identified 43 publications stemming from 18 independent prospective longitudinal studies, including 20 new publications and 2 new longitudinal studies compared to previous systematic reviews. We demonstrate that people with HIV develop a robust initial immune response following HPV vaccination and that vaccines were regarded as safe for all three vaccine types evaluated. Among people living with HIV (PLHIV), who were seronegative for the vaccine type HPV infection prior to vaccination, seroconversion rates were high 28 weeks after receiving the first vaccine dose: more than 94% of PLHIV seroconverted for HPV-16/18 across three vaccines. Seropositivity remained high among people who received 3 doses despite some declines in antibody titers, with more pronounced decline among PLHIV as compared to people without HIV. We conducted subgroup and meta-regression analyses to assess heterogeneity by vaccine, participant, and study characteristics. Four trials reported immunogenicity results by HIV disease stage. There is modest evidence to suggest that antibody titers and seroconversion rates were lower among PLHIV who had lower CD4 cell counts or who had detectable HIV plasma viral loads.

Our findings demonstrate important data gaps in the evidence-base for vaccine effectiveness against HPV infections and associated disease among PLHIV. The few studies identified offered low quality evidence with only 2 clinical trials including a placebo arm. Issues with measurement of the outcome, and baseline serostatus limit the inferences that can be drawn.

**Implications of all the available evidence**

PLHIV can develop a robust humoral immune response following HPV vaccination. The vaccine is safe and well tolerated, with few serious adverse events. Evidence of vaccine efficacy on biological outcomes following vaccination was generally of low quality. The evidence-base is lacking from well-designed studies that account for the underlying HPV infection status, timing of infection, and have a sample size and follow-up time that are appropriate for estimation of efficacy of HPV vaccine in PLHIV. The evidence of a robust immune response generated among all PLHIV who have received HPV vaccination supports cervical cancer elimination efforts to increase HPV vaccination coverage including in high HIV prevalence settings.

Alt-text: Unlabelled box

## Introduction

The Human Papilloma Virus (HPV) is a common sexually transmitted infection. High-risk (HR) oncogenic HPV types cause cervical cancer and other anogenital cancers in women and men.^1^, ^2^, ^3^ Currently there are four licensed HPV vaccines (Cervarix, Gardasil, Gardasil-9, Cecolin),^4^^,^^5^ which protect against acquisition of HR-HPV-16 and HPV-18. Cervarix and Cecolin (recently approved) are bivalent HPV vaccines whereas Gardasil is a quadrivalent vaccine. The nonavalent vaccine (Gardasil-9) also protects against five additional HR-types (HPV31/33/45/52/58).^6^ HPV-16/18 and HPV-16/18/31/33/45/52/58 cause approximately 70% and 90% of all cervical cancer cases, respectively.^7^ In addition, HPV-16 and 18 also cause the large majority of anal cancers in men and women^8^ and HPV-6 and 11 cause over 90% of genital warts.^9^

HPV vaccination produces a long-term immune response lasting at least 8–14 years in the general population.^10^, ^11^, ^12^, ^13^ Since the first HPV vaccine was licensed in 2006, HPV vaccination programmes have been implemented in approximately 100 countries.^14^^,^^15^ Epidemiological studies have shown reductions in the prevalence of HPV vaccine types and high-grade cervical intraepithelial neoplasia (CIN2+) in young women 5 to 9 years as well as declines in anogenital warts diagnoses after the introduction of national vaccination campaigns.^14^^,^^16^ Declines in anogenital warts diagnoses in women and men following vaccination have also been observed.^14^ In 2020, the World Health Organization (WHO)’s Member States adopted the Global strategy for cervical cancer elimination as a public health problem. Reaching the WHO's elimination target requires countries to reach 90% coverage of HPV vaccination in girls, 70% coverage of cervical cancer screening, and 90% treatment and management of both precancerous lesions and invasive cancer cases.^17^

The burden of HPV-associated diseases is disproportionately concentrated in low- and middle-income countries, where approximately 80% of cervical cancer diagnoses and deaths occurred in 2020.^3^^,^^15^^,^^18^^,^^19^ Sub-Saharan Africa has the highest burden of HPV infections and cervical cancer diagnoses worldwide, with age-standardised cervical cancer incidence and mortality rates approximately three times larger than the global average.^20^ The concomitant burden of HIV contributes to the disparity.^15^, ^16^, ^17^, ^18^ It is estimated that 23% and 53% of cervical cancer cases are attributable to HIV in eastern and southern Africa, respectively, compared to 5% globally.^21^

Meta-analyses of longitudinal studies suggest that HIV amplifies HPV-associated disease burden in multiple ways.^22^ Compared to HIV-negative people, people living with HIV (PLHIV) are at increased risk of acquiring HPV infection, developing precancerous lesions and are less likely to clear their HPV infection and their precancerous lesions regress more slowly.^21^, ^22^, ^23^, ^24^ WLHIV have a six-fold risk of developing cervical cancer compared to women without HIV.^21^ Higher rates of anal cancer are also observed in PLHIV, particularly among men who have sex with men.^25^

The efficacy of HPV vaccination in individuals without HIV has been evaluated in large randomized controlled trials (RCTs) and summarized in systematic reviews.^26^, ^27^, ^28^, ^29^ A similar evidence-base does not exist for PLHIV; instead several small immunogenicity studies have been conducted.^30^^,^^31^ Few studies have assessed vaccine impact on HPV-related biological outcomes among PLHIV.^31^^,^^32^ Five systematic reviews of the bivalent or quadrivalent HPV vaccines among PLHIV based on searches conducted before 2019 have been published^33^, ^34^, ^35^, ^36^, ^37^; none included results on Gardasil-9. Three narrative reviews summarized safety and immunogenicity results^34^^,^^35^^,^^37^ and biological outcomes^37^ from seven RCTs or less. Only two reviews provided a quantitative summary^33^^,^^36^ of HPV seroconversion following vaccination from fourteen studies^33^ and differences in adverse events (AEs) or geometric mean antibody titers (GMTs) between vaccine and placebo groups from three^33^ and four RCTs.^36^ The reviews suggest the vaccines are safe and immunogenic. Despite highlighting heterogeneity across studies, none of the reviews formally assessed the influence of vaccine type, number of vaccine doses, timing of measurement or baseline serostatus.

The WHO has adopted HPV vaccine and cervical cancer-screening based strategies to accelerate cervical cancer elimination. Given the elevated risk of anogenital cancers among PLHIV, the purpose of the study is to evaluate the efficacy of HPV vaccination in PLHIV to inform clinical and public health guidelines and identify where important data gaps remain. We conducted a systematic review and meta-analysis to provide summary estimates in three domains: immunogenicity, safety, and efficacy of HPV vaccination in PLHIV taking into account baseline HPV DNA and/or HPV serostatus, when possible. We also conducted subgroup analyses by age, sex, vaccine type and dose, and HIV disease status from available data.

## Methods

This systematic review reporting follows the *Preferred Reporting Items for Systematic Reviews and Meta-Analyses* (PRISMA).^38^ The review has not been registered and the protocol has not been published.

### Search Strategy and data extraction

We searched for peer-reviewed articles in PubMed, Medline and Embase using terms for HIV, HPV, HPV-associated diseases and for any of the HPV vaccines published by February 11th, 2021 (full search terms in Supplement Table S1). We also searched clinicaltrials.gov and the WHO clinical trial database for online records of published or unpublished trial results on HPV vaccination in PLHIV.^39^^,^^40^

Peer-reviewed articles and online clinical trial records (henceforth referred to as ‘publications’) were eligible for inclusion if they reported prospectively collected longitudinal data from clinical trials with one or more arms on the safety, immunogenicity, or any relevant HPV related biological endpoints following prophylactic vaccination of PLHIV with any of the licensed HPV vaccines. Studies were included regardless of how many vaccine doses were given, but we excluded studies if they did not report results by dose (i.e. studies were excluded if the seropositivity results included a mix of people with different number of doses received). Mathematical modeling studies, qualitative studies or literature reviews, studies that did not report outcomes by HIV status, retrospective studies, and studies which did not report any immunogenicity results by HPV type were excluded. No language, date, or location restrictions were placed. We first screened publication titles and abstracts for inclusion. Full texts of selected publications were then retrieved to confirm eligibility. Finally, the references of publications were searched to identify any additional ones.

For each included publication, we extracted available estimates or data to derive estimates. Where possible, study estimates were extracted by baseline HPV status, and were categorized as “unknown HPV status” otherwise. We included the following outcomes in the study: immunogenicity (proportion seropositive, GMTs), HPV-related biological endpoints (e.g., virological, histological, clinical) and vaccine safety (e.g., AEs). We extracted data on endpoints specific to the HPV types included in the vaccines (e.g., only HPV-16/18 for the bivalent vaccine and HPV6/11/16/18 for the quadrivalent). We also extracted information on study and participant characteristics such as sex, age, use of antiretroviral therapy (ART), CD4 cell count, location, study design, follow-up time, and number of vaccine doses.

The search and screening of the publications was done independently by two reviewers (LS and MEB or NS), using Covidence software.^41^ Data were extracted by LS or NS and then reviewed by MEB, NS or LS; differences were resolved by consensus.

### Data analysis

Results were presented as forest plots (seropositivity), line graphs (GMT) or tabulations (other outcomes) by timing of the measurement after administration of the first vaccine dose (i.e., 28 weeks, 29-99 weeks, ≥100 weeks) and by baseline HPV seropositivity status, by vaccine type, and by relevant participant characteristics where possible.

When multiple publications reported on the same outcome from one trial (i.e., from the same population), we selected results, in order of priority, from the i) peer-reviewed article, ii) the publication providing the longest follow-up data, or iii) the publication with the largest sample size to use in pooled estimates. Most studies reported immunogenicity and GMT results based on the competitive Luminex immunoassay (cLIA), and cLIA results were favored in pooled estimates in studies reporting estimates from multiple assays. For publications that reported on HPV seronegative participants at baseline without reporting baseline GMT levels, we used the cLIA assay cut-off for HPV seropositivity as baseline value. We grouped results based on cLIA, LIA and ELISA assays together as they produced qualitatively comparable GMT results and presented results based on neutralization assays separately as they produced substantially higher GMT values.

We pooled independent estimates of seropositivity results following vaccination using DerSimonian-Laird random effects models,^42^ stratified by number of doses received and timing of measurement, and presented them separately for participants HPV negative at baseline and of “unknown HPV status”. For meta-analyses of seropositivity after vaccination, outcomes using proportions were transformed using arcsine transformation and 95% confidence intervals (95%CI) were calculated, with results presented on the original scale.^43^ We assessed heterogeneity across estimates using the I² statistic.^44^ We also performed subgroup analyses by participant and study characteristics (e.g., sex, age, HIV treatment status, region) using meta-regression, combining HPV seronegative and seropositive at baseline for the subgroup analyses. All analyses, and figures were created using ‘R’ version 1.2.1355, and the ‘metafor’ package was used for meta-analyses.^45^

The main analyses on seroconversion (seropositivity among those seronegative at baseline) and GMT titers focused on results from participants who were HPV seronegative for the vaccine-type at baseline and subsequently seroconverted since this represent the most reliable evidence on the immune response to vaccination. In addition, and for completion, we also analysed data for the participants who were HPV seropositive or had unknown serostatus for the vaccine type HPV at baseline for completion. We conducted subgroup analyses for the outcomes by age, sex, vaccine, and HIV disease indicators.

### Study quality

We tailored the Newcastle-Ottawa-Scale to our specific research question to evaluate study quality and risk of bias (across domains of study design, selection bias, misclassification bias, measurement error and internal validity) separately for the three main types of outcomes seropositivity and antibody titers (based on 12 criteria), biological endpoints (based on 15 criteria), allocating one point per criteria as detailed in Supplement Table S2A-B. The scale was applied independently by two researchers (LS & MMR) and differences were resolved by a third researcher (MCB).

### Role of the funding source

The study was partly funded by the World Health Organization. WHO contributed to study design, and interpretation of the results. The other funding sources had no role in this work. LS and MMR had full access to all the data used in the study and had final responsibility for the decision to submit for publication.

## Results

### Search results

**Figure 1:**
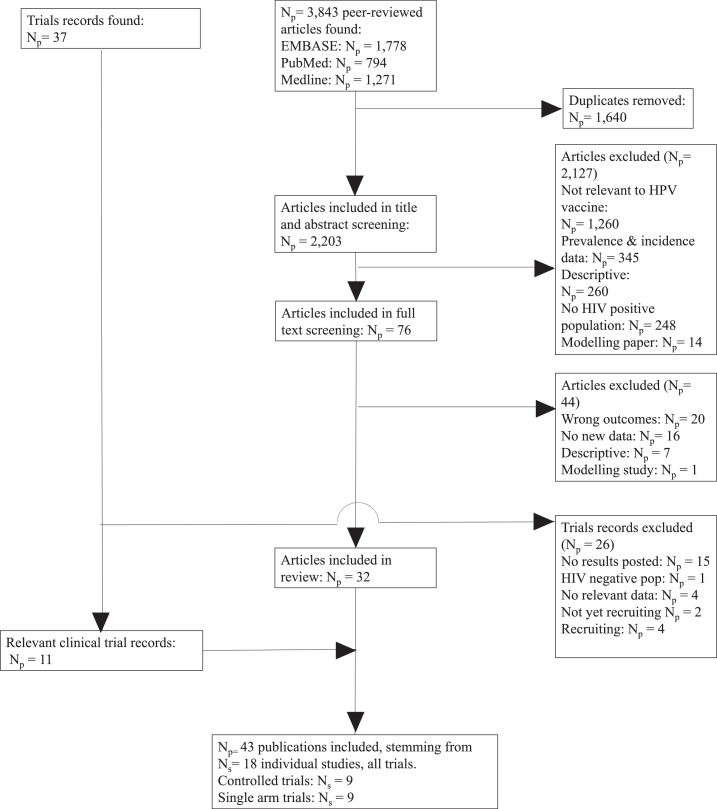
**Search results and study selection**. Abbreviations: *N_p_* = Number of publications, *N_s_*= Number of Studies.

Of the 3843 peer-reviewed articles identified through our literature searches, 76 underwent full text review and 32 met our inclusion criteria (Figure 1). We identified 37 online trial records from the trial database searches, of which 11 reported additional information to peer-reviewed articles and were included. Only one online trial record did not have a peer-reviewed article associated with it.^46^ In total, we included 43 publications (Number of publications *N_p_*=32 published articles and 11 online trial records) from 18 independent prospective longitudinal studies (Number of studies, *N_s_*=18), with approximately 3900 participants.

Table 1 summarises the study and participant characteristics of the included trials (10 controlled trials and 8 single arm trials). Supplement Table S3 presents additional details for each publication. Most studies were conducted either in the Americas (*N_s_*=8) or Europe (*N_s_*=5), evaluated the quadrivalent (*N_s_*=15) rather than the bivalent (*N_s_*=4) or nonavalent (*N_s_*=1) vaccines, and evaluated three doses (*N_s_*=18) rather than four doses (*N_s_*=3). No studies of Cecolin vaccine, nor studies evaluating one or two doses, in PLHIV were found. Controlled trials compared the quadrivalent (*N_s_*=4) or bivalent vaccines (*N_s_*=1) to a placebo, the bivalent to the quadrivalent vaccine (*N_s_*=2), three versus four doses (*N_s_*=2), or vaccination between HIV positive and HIV-negative individuals (*N_s_*=3; Table 1A). In addition, five single arm trials and two RCTs also included historical controls of (un)vaccinated HIV-negative individuals or unvaccinated PLHIV. Studies were small (median trial sample size: 97 for the controlled trials, 150 for the single arm trials) with average follow-up duration of 1-2 years and the longest follow-up of 8 years.^47^

**Table 1:** Summary of the study and participant characteristics of the *N_s_* = 18 independent trials included (Details of the *N_p_* = 43 publications included are presented in Supplement Table S3).

	Single arm trials (Longitudinal studies)		Controlled trials	
	Number (*N_s_* = 8)	References (*N_p_* = 22)	Number (*N_s_* = 10)	References (*N_p_* = 21)
**A) Study characteristics**				
***WHO region***				
Africa	1	^30^^,^^48^	1	^49^^,^^50^
America	4	^32^^,^^46^^,^^47^^,^^51^, ^52^, ^53^^,^^54^^,^^55^^,^^56^^,^^75^, ^76^, ^77^, ^78^, ^79^	4	^62^^,^^63^^,^^64^^,^^65^^,^^66^^,^^67^^,^^68^^,^^69^
Europe	1	^70^	4	^31^^,^^71^^,^^83^, ^84^, ^85^, ^86^, ^87^, ^88^, ^89^
Asia	1	^79^^,^^80^	0	−
Mix	1	^81^^,^^82^^,^^83^	1	^84^^,^^85^
***Study year (mid-point)***				
2000-2010	3	^30^^,^^32^^,^^47^^,^^52^^,^^53^^,^^79^^,^^56^^,^^57^^,^^77^, ^78^, ^79^	2	^62^^,^^66^^,^^49^^,^^69^
Post 2010	4	^46^^,^^51^^,^^54^^,^^55^^,^^48^^,^^58^^,^^69^^,^^80^	8	^31^^,^^63^^,^^71^^,^^64^^,^^65^^,^^67^^,^^68^^,^^83^, ^84^, ^85^, ^86^, ^87^, ^88^, ^89^^,^^84^^,^^85^
Not reported	1	^70^	0	−
***Vaccine type***				
Nonavalent	0	−	1	^77^^,^^78^
Quadrivalent	8	^30^^,^^32^^,^^46^, ^47^, ^48^, ^49^^,^^51^, ^52^, ^53^^,^^54^^,^^58^, ^60^, ^59^^,^^48^^,^^75^, ^76^, ^77^, ^78^, ^79^^,^^70^^,^^80^^,^^83^	7	^31^^,^^62^^,^^63^^,^^71^^,^^64^^,^^65^^,^^66^^,^^67^^,^^69^^,^^83^, ^84^, ^85^, ^86^, ^87^^,^^84^^,^^85^
Bivalent	0	−	4	^31^^,^^49^^,^^50^^,^^68^^,^^72^^,^^73^^,^^76^^,^^84^^,^^85^
***Vaccine doses***				
3 doses	8	^31^^,^^46^^,^^47^^,^^62^^,^^63^^,^^64^^,^^79^^,^^65^^,^^66^^,^^67^^,^^72^, ^73^, ^74^^,^^59^^,^^68^^,^^69^^,^^83^, ^84^, ^85^, ^86^, ^87^	10	^32^^,^^81^^,^^52^^,^^53^^,^^56^^,^^60^^,^^77^^,^^78^^,^^84^^,^^85^
4 doses	1	^57^^,^^60^	2	^63^^,^^66^^,^^67^^,^^69^
***Study design***				
Randomized controlled trials:	N/A	−	7	^31^^,^^62^^,^^63^^,^^71^^,^^64^^,^^65^^,^^66^^,^^67^^,^^49^^,^^50^^,^^69^^,^^83^, ^84^, ^85^^,^^76^^,^^84^^,^^85^
*Quadrivalent vaccine vs Placebo*	N/A	*−*	4	^62^^,^^63^^,^^71^^,^^64^^,^^65^^,^^66^^,^^67^^,^^69^^,^^74^
*Bivalent vaccine vs Placebo*	N/A	*−*	1	^49^^,^^50^
*Quadrivalent vs Bivalent vaccines*	N/A	−	2	^31^^,^^72^^,^^73^^,^^76^^,^^84^^,^^85^
*Three vs four doses of quadrivalent vaccine*	N/A		2	^63^^,^^66^^,^^67^
Non randomized controlled trials:^+^	N/A	−	3	^68^^,^^75^^,^^77^^,^^78^
Trials with historical controls (HIV positive unvaccinated, HIV negative vaccinated or HIV negative unvaccinated)	5	^30^^,^^51^^,^^52^^,^^54^^,^^55^^,^^48^^,^^61^	2	^62^^,^^63^
***Number of trial participants (at entry)***				
Median across studies (IQR)	150 (99-307)		97 (91–219)	
**Follow-up duration**				
<1 year	3	^62^^,^^68^^,^^74^	4	^30^^,^^82^^,^^51^^,^^54^^,^^55^^,^^57^^,^^61^^,^^69^^,^^70^^,^^88^, ^89^, ^90^
1-2 years	6	^31^^,^^46^^,^^79^^,^^49^^,^^50^^,^^59^^,^^72^^,^^73^^,^^75^^,^^76^	2	^58^^,^^84^^,^^85^
>2 years (maximum: 8 years)	3	^47^^,^^63^^,^^64^^,^^65^^,^^66^^,^^67^^,^^48^^,^^69^	4	^81^^,^^82^^,^^51^, ^52^, ^53^^,^^54^^,^^71^^,^^55^^,^^56^^,^^58^^,^^69^^,^^80^
**B) Participant characteristics**				
***Sex***				
Female	5	47, 48, 49^,^^51^, ^52^, ^53^^,^^54^^,^^58^, ^60^, ^59^^,^^58^^,^^59^^,^^70^^,^^80^^,^^83^	2	^49^^,^^50^^,^^84^^,^^85^
Male	2	^32^^,^^46^^,^^57^^,^^60^^,^^61^	2	^71^^,^^68^^,^^74^
Female and Male	1	^30^^,^^48^	6	^31^^,^^62^^,^^63^^,^^64^^,^^65^^,^^66^^,^^67^^,^^69^^,^^72^^,^^73^^,^^86^, ^87^, ^88^, ^89^
***Age category***^/^				
Children (≤18 years)^♯^	3	^30^^,^^54^^,^^48^^,^^70^	2	^62^^,^^63^^,^^66^^,^^67^^,^^69^
Adults (>18 years)	3	^32^^,^^46^^,^^51^^,^^57^^,^^77^, ^78^, ^79^	6	^31^^,^^71^^,^^64^^,^^65^^,^^49^^,^^50^^,^^68^^,^^83^, ^84^, ^85^^,^^87^, ^88^, ^89^
Mix of adults and children	4	47, 48, 49^,^^52^^,^^53^^,^^58^, ^60^, ^59^^,^^58^^,^^80^^,^^83^	2	^75^^,^^84^^,^^85^
***Proportion of participants on ART (Baseline)***				
100%	1	^70^	1	^76^
67-99%	3	^30^^,^^32^^,^^54^^,^^55^^,^^48^^,^^60^^,^^61^	6	^31^^,^^63^^,^^71^^,^^64^^,^^66^^,^^67^^,^^83^, ^84^, ^85^^,^^77^
25-66%	2	^81^^,^^52^^,^^53^^,^^79^^,^^56^^,^^83^	1	^85^
<25%	0	−	1	^50^
Not reported	5	^46^^,^^47^^,^^82^^,^^51^^,^^75^, ^76^, ^77^^,^^80^	7	^62^^,^^65^^,^^49^^,^^68^^,^^69^^,^^75^^,^^78^^,^^84^
***Baseline CD4 count (Mean or Median)***				
<350 cells/mm^3^	1	^81^^,^^82^^,^^83^	0	−
350–500 cells/mm^3^	1	^47^	1	^50^
>500 cells/mm^3^	4	^30^^,^^32^^,^^51^^,^^52^^,^^54^^,^^55^^,^^79^^,^^48^^,^^58^^,^^60^^,^^61^	8	^31^^,^^62^^,^^63^^,^^71^^,^^64^^,^^65^^,^^66^^,^^67^^,^^69^^,^^83^, ^84^, ^85^, ^86^, ^87^, ^88^^,^^85^
Not reported	4	^53^^,^^56^^,^^57^^,^^59^^,^^70^^,^^80^	4	^49^^,^^68^^,^^78^^,^^84^
**C) Study outcomes**				
***Immunogenicity***				
Seropositivity^a^: Stratified by*:*	5	^30^^,^^32^^,^^81^^,^^82^^,^^51^, ^52^, ^53^^,^^54^^,^^79^^,^^56^^,^^48^^,^^57^^,^^60^^,^^61^^,^^83^	9	^31^^,^^62^^,^^63^^,^^66^^,^^49^^,^^50^^,^^68^^,^^69^^,^^72^^,^^73^^,^^75^^,^^77^^,^^78^^,^^84^^,^^85^
*CD4 count/nadir*	1	^82^	1	^62^
*ART status*	1	^53^	0	−
*Viral suppression*	0	−	0	−
Geometric mean antibody titre (GMT): Stratified by:	6	^30^^,^^32^^,^^46^^,^^81^^,^^82^^,^^51^, ^52^, ^53^^,^^54^^,^^48^^,^^57^^,^^77^, ^78^, ^79^^,^^83^	6	^31^^,^^62^^,^^63^^,^^50^^,^^69^^,^^73^^,^^76^^,^^77^^,^^84^^,^^85^
*CD4 count/nadir*	1	^82^	1	^62^
*ART status*	1	^53^	0	−
*Viral suppression*	1	^54^	0	−
***Biological outcomes***				
*Genital warts*	2	^46^^,^^55^	0	−
*Anogenital DNA infection*	4	^32^^,^^46^^,^^47^^,^^82^^,^^55^^,^^79^	3	^31^^,^^71^^,^^64^^,^^73^
*Cytology (anal/cervical)*	0	−	2	^71^^,^^64^^,^^65^
*Histology (CIN2+)*	1	^55^	0	−
***Safety***				
*AEs (possibly, probably or definitely related to vaccine)*	3	^32^^,^^46^^,^^52^^,^^53^^,^^79^^,^^56^^,^^57^^,^^60^^,^^61^	2	^62^^,^^64^^,^^65^^,^^66^^,^^69^
*Any AEs*	7	^30^^,^^32^^,^^46^, ^47^, ^48^, ^49^^,^^51^, ^52^, ^53^^,^^54^^,^^58^, ^60^, ^59^^,^^48^^,^^57^^,^^58^^,^^60^^,^^61^^,^^80^^,^^83^	7	^31^^,^^62^^,^^71^^,^^64^^,^^65^^,^^66^^,^^49^^,^^50^^,^^69^^,^^73^^,^^74^^,^^87^, ^88^, ^89^^,^^84^^,^^85^
*(S)AEs*	7	30, 32, 46, 72, 52, 53, 55, 60, 75, 77, 78, 79, 59, 90, 48, 49, 91	7	^31^^,^^62^^,^^71^^,^^64^^,^^65^^,^^66^^,^^49^^,^^50^^,^^69^^,^^83^, ^84^, ^85^^,^^87^, ^88^, ^89^^,^^84^^,^^85^

a The proportion of the population that is seropositive for any of the HPV vaccine types at any point in time regardless of baseline HPV status; (S)AEs = (serious) Adverse Events, CIN = Cervical intraepithelial neoplasia; *N_p_*: Number of publications; *N_s_*: Number of independent trials; ^//^Children if participants are < 18 years old and adults if ≥ 18 years old; ^♯^ Range between 7 and 18 years old; ^❖^ Historical control group based on participants recruited in a different study, time, location; ^+^All compare vaccine in HIV positive and negative participants.

Trials included females only (*N_s_*=7), males only (*N_s_*=4) or both sexes (*N_s_*=7), and children aged between 7 and 18 years old (*N_s_*=5), adults aged >18 years old (*N_s_*=9), or a mixed age population (*N_s_*=6) (Table 1B). Most trials reported on immunogenicity results (seropositivity: *N_s_*=14; antibody titers: *N_s_*=12) (Table 1C). All eight trials that reported biological endpoints following vaccination were among adults or mixed age participants (i.e., none in only children). Fifteen trials reported varied levels of information on HIV treatment and/or CD4 cell counts at baseline but very few reported outcomes stratified by HIV disease indicators. Information on the safety of the vaccine was reported for fifteen trials.

### Study quality

**Figure 2:**
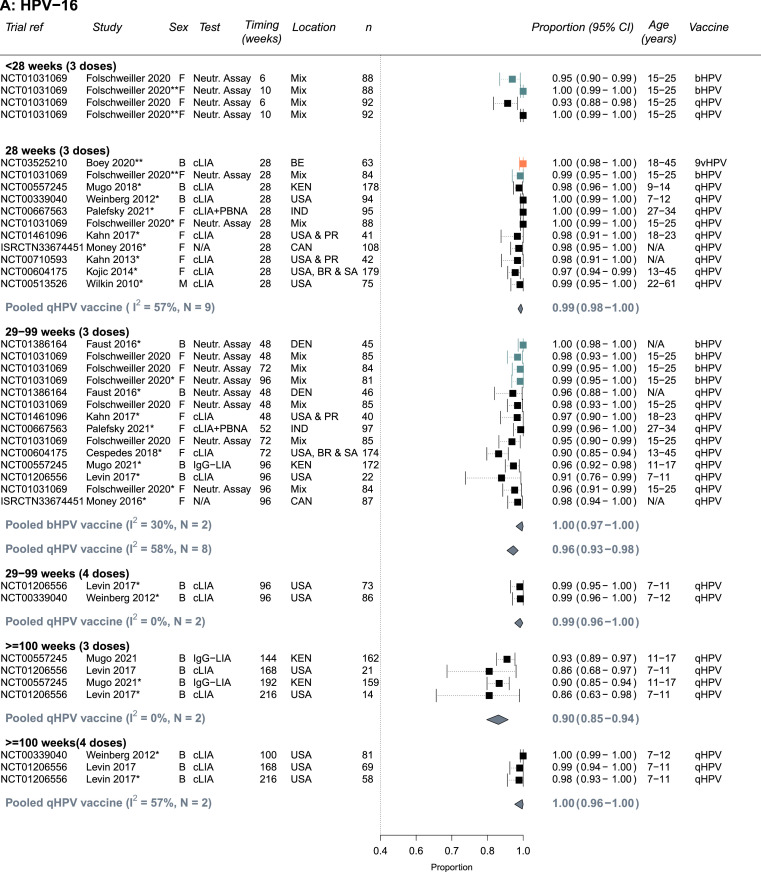

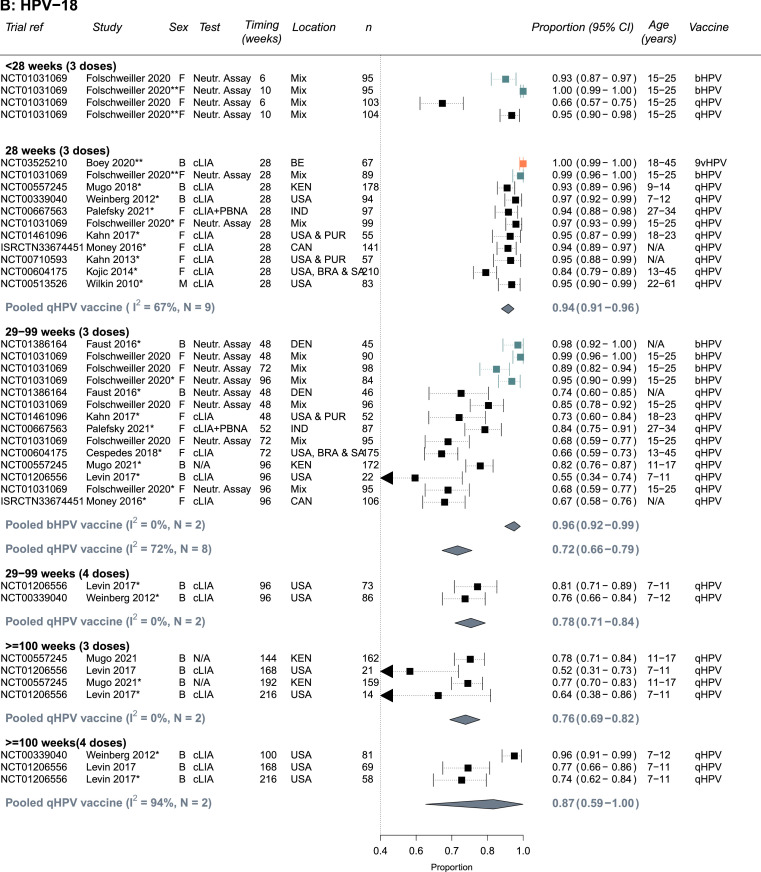

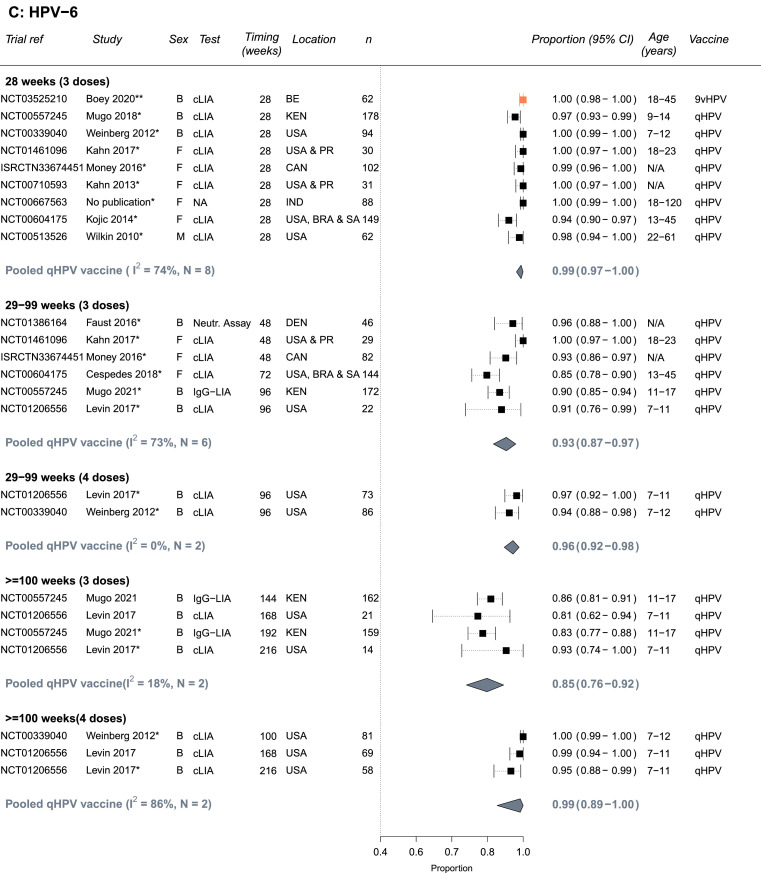

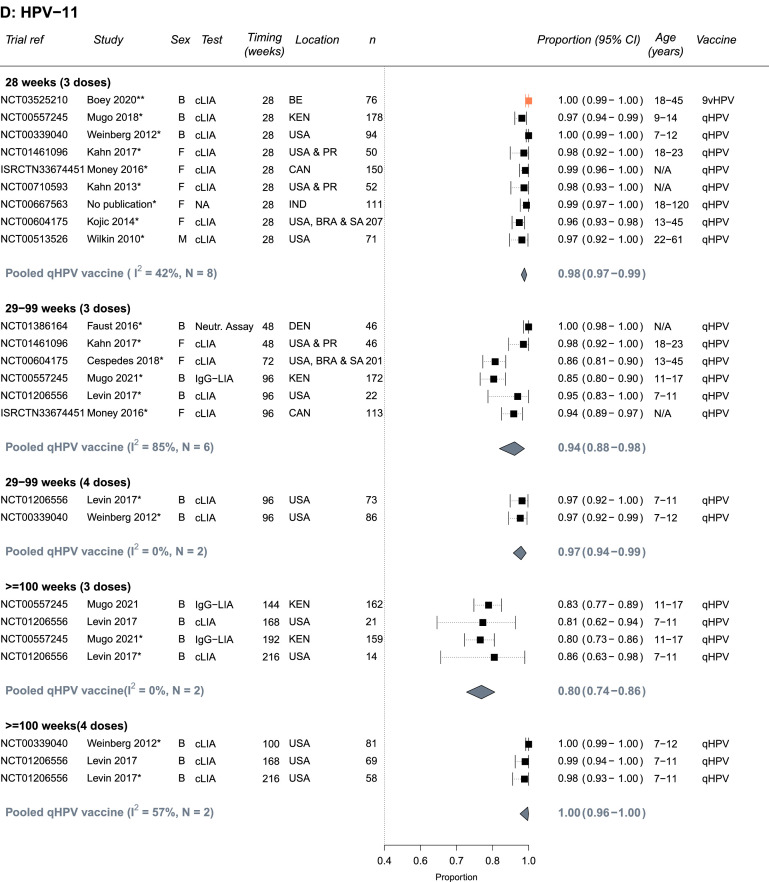
**Seropositivity to A) HPV-16, B) HPV-18, C) HPV-6 and D) HPV-11 following vaccination with the bivalent (bHPV), quadrivalent (qHPV), or nonavalent (9vHPV) vaccines among PLHIV who were seronegative for the specific HPV type at baseline** (Number of independent trials, *N_s_* =13). Estimates are stratified by number of doses and timing of measurement since the first dose in the vaccination schedule. One asterisk(*) indicates which study estimates were included in pooled estimates (one per study - the longest follow up time). Two asterisks (**) indicates there was only single estimate for a given vaccine type, which was not pooled. Vaccine types are colour-coded: black = qHPV, teal = bHPV, and orange = 9vHPV. Abbreviations: Both (B), female (F) or male (M); N= Trial sample size; BR=Brazil, CAN=Canada, Den=Denmark, IND=India, KEN=Kenya, PR= Puerto Rico, SA=South Africa, USA=United States of America; Age ranges are in years; cLIA= chemiluminescence immunoassay, Neutr. assay = neutralization assay, IgG-LIA = line immunoassay, PBNA = pseudovirion-based neutralisation assay.

The quality assessments for each outcome are presented in Supplement Tables S9-S10 based on the criteria in Table S2A-B. Most studies were conducted in relatively healthy PLHIV and/or PLHIV receiving ART; few studies included patients with low CD4 cells counts or untreated HIV limiting the representativeness of results for all PLHIV (e.g., of the studies reporting a baseline CD4 count, two studies reported a mean or median CD4 below 500 cells/μL; Table 1, Supplement Tables S3, S9-S10). Immunogenicity outcomes received higher quality scores than biological outcomes: Two seropositivity and two GMT titers outcome estimates scored less than 9 out of a maximum of 12 points whereas 14 biological outcome estimates scored less than 12 out of a maximum of 16 points. Estimates of seropositivity following vaccination among seronegative individuals received the highest scores (9-12), as the baseline status means the group is less likely to suffer from misclassification bias, and these estimates were used in our main analysis (Figure 2). For biological outcomes, scores were lost across different bias domains; most scores were lost due to the lack of a valid comparable control group (study had problems with internal validity), small sample sizes or lack of sample size calculation (measurement error), and poor specificity of the outcome (i.e., not specific to the HPV types included in the vaccine tested, measurement error), affecting the interpretability and the validity of biological results and/or power to detect differences. Given the lower quality of biological outcomes, we did not pool results for biological outcomes, and we present a summary of main results in Supplement Table S6, and full results in Table S7.

### Immunogenicity results – seropositivity

Almost all HPV seronegative PLHIV seroconverted to vaccine specific types and remained seropositive 28 weeks after the first vaccine dose (Figure 2A-D). The summary estimate of proportion of PLHIV who seroconverted by 28 weeks for the quadrivalent vaccine were 0.99 (95%CI: 0.98-1.00, *N_s_*=9, I2=57%), 0.94 (95%CI: 0.91-0.96, *N_s_*=9, I2=67%), 0.99 (95%CI: 0.97-1.00, *N_s_*=8, I2=74%), and 0.98 (95%CI: 0.97-0.99, *N_s_*=8, I2=42%), for HPV-16, HPV-18, HPV-6, and HPV-11, respectively. Similarly, high seroconversion levels for HPV-16 and HPV-18 were reported for the bivalent vaccine (0.99, 95%CI: 0.95-1.00 and 0.99, 95%CI: 0.96-1.00, *N_s_*=1, respectively) and for the nonavalent vaccine (1.00, 95%CI: 0.98-1.00 and 1.00, 95%CI: 0.99-1.00, *N_s_*=1, respectively), and for the five additional HR-HPV types included in the nonavalent vaccines (Supplement Table S3).

Seropositivity levels after the third dose remained high despite some declines over time, the decline was greater for HPV-18 and with the quadrivalent vaccine (29-99 weeks: 0.72, 95%CI: 0.66-0.79, *N_s_*=8, I2=72%) than the bivalent vaccine (29-99 weeks: 0.96, 95%CI: 0.92-0.99, *N_s_*=2, I2=0%) (Figure 2B). Consequently, increases in seropositivity after a fourth vaccine dose were more pronounced for HPV-18 with the quadrivalent vaccine, with higher seropositivity observed at ≥100 weeks (29-99 weeks: 0.78, 95%CI: 0.71-0.84, *N_s_*=2, I2=0% vs ≥100 weeks: 0.87, 95%CI: 0.59-1.00, *N_s_*=2, I2=94%) (Figure 2B). No information was available for the nonavalent vaccine after 28 weeks. Seropositivity results for PLHIV with mixed seropositive HPV status at baseline were similar to those HPV seronegative at baseline (Supplement Figure S1A-D). For the quadrivalent vaccine, the heterogeneity across studies for pooled estimates for HPV-18 and HPV-6 (I2 values) was larger than for HPV-16 and HPV-11. Heterogeneity was also higher across study estimates for pooled estimates with timing between 29 and 99 (I2=58-85%).

In subgroup analyses (combining HPV seronegative and seropositive at baseline), results were generally similar by sex, age, and region across HPV types and timing of measurements (Supplement Table S4A-D). The only statistically significant differences were that the proportion who were seropositive for HPV-18 after 3 doses was higher for participants receiving the bivalent vaccine than the quadrivalent vaccine at week 28 and weeks 29–99. Seropositivity results between adults and children were similar albeit potentially seropositivity after vaccination was a little lower in adults. However, the number of estimates pooled in the subgroup analyses pooled was low, which also reduces ability to detect differences between subgroups. It was not possible to conduct subgroup analysis by CD4 level, viral suppression or being on ART or not given the few estimates and/or the heterogeneity in exposure categories used.

### Immunogenicity results – average antibody titers over time

**Figure 3:**
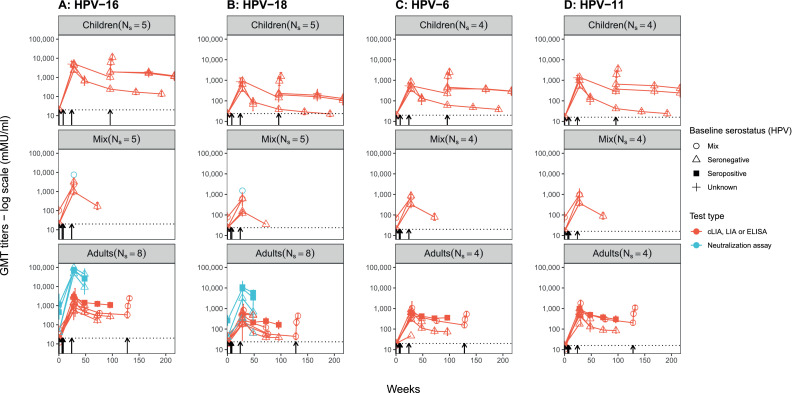
**Average geometric mean antibody titre (GMT) for A) HPV-16, B) HPV-18, C) HPV-6, D) HPV-11 over time after administration of the quadrivalent vaccine in different PLHIV study populations (children: <18, adults: ≥18, and mix: both children & adults), stratified by baseline HPV status and type of test (*N_s_* = 11 trials)**. On each panel, each line (or unique data point when only one time point was reported) is the mean from a different study. N_s_ on each panel indicate the number of studies included. Arrows indicate timing of vaccine doses. Dotted line indicates cut-off value for seropositivity for the cLIA assay. Mean (range) GMT levels (excluding neutralization assay results) at 28 weeks across studies are higher for HPV-16 (mean: 2239, range 504-5173 mMu/ml).

Antibody titers increased by week 28 and were higher for HPV-16 than HPV-18 and HPV-6/11 in those receiving the quadrivalent vaccine as shown in Figure 3. GMT levels for HPV-16 at week 28 tended to be slightly higher for children than adults (Figure 3). GMT levels declined over time after 28 weeks (with three doses) for the four HPV vaccine types, especially for HPV-18. However, none of the average GMT measures fell below the seropositivity cut-off. Two studies reported results after a fourth dose (administered after 72 or 128 weeks) of the quadrivalent vaccine showed an increase in antibody titers for all four HPV vaccine types at 96 or 128 weeks in children and adults (Figure 3). One trial evaluating the nonavalent vaccine among men reported similar increases at 28 weeks, albeit more modest than in some of the other trials on the quadrivalent vaccine (Supplement Figure S2). Finally, average GMT levels for HPV-18 were higher in participants receiving the bivalent than the quadrivalent vaccine (Supplement Figures S2, S4). Supplement Figures S2, S3, S4 present additional results for each study.

### Immunogenicity results - by HIV status or disease stage

Differences in seropositivity between HIV negative and PLHIV after three doses were small albeit statistically significant for HPV-16, HPV-18 (28 weeks, 29-99 weeks) and HPV-6 (29-99 weeks) (Supplement Figure S5A-D). The difference in the pooled proportion seropositive between HIV negative and PLHIV was largest at 29-99 weeks for HPV-18 (0.94, 95%CI: 0.90-0.98, N_s_=3, I2=68% vs 0.72, 95%CI: 0.60-0.82, N_s_=5, I2=78%, *p*-value<0.001) and remained relatively small for HPV-16 (1.00, 95%CI:0.99-1.00, N_s_=3, I2=32% vs 0.97, 95%CI: 0.93-0.99, N_s_=5, I2=39, p-value<0.001).

Antibody titers following vaccination declined for both HIV-negative participants and PLHIV but declined further among PLHIV participants over time (Supplement Figure S7, Table S5). For example, the HIV-positive versus negative ratio of GMT levels at 28 weeks across studies ranged between 0.4-0.8 for HPV-16 (*N_s_*=5) and 0.3-0.8 for HPV-18 (*N_s_*=5).

Only four studies reported seroconversion and/or GMT levels stratified by HIV disease stage (CD4-cell counts,^81^^,^^82^ CD4 cell count percentages/fraction,^62^ plasma viral suppression^51^) or ART status^52^^,^^53^ (Supplement Figure S6A-D, supplement table S11). One trial reported high GMT titers at 28 weeks for PLHIV in any CD4-cell counts subgroups although GMT titers and the proportion that seroconverted following vaccination were lower for PLHIV with CD4 ≤200 (HPV-16, HPV-18, HPV-6, HPV-11: 0.93, 0.75, 0.84, 0.92 if CD4 ≤200 cells/μL and 0.99, 0.91, 0.96, 0.98 if >350 cells/μL, respectively, *N_s_*=2, Supplement Table S3).^81^^,^^82^ A second trial reported a positive and significant correlation between high CD4% and antibody titers but did not provide stratified estimates^63^ whereas a third trial found no difference in antibody titers by CD4% nadir^62^ (Supplement Figure S6A-D). Finally, a fourth trial reported higher seropositivity rates and antibody titers in PLHIV on ART than not on ART^52^^,^^53^ while another one reported higher antibody titers for all four HPV vaccine types among PLHIV virally suppressed compared to non-suppressed.^54^

### Biological endpoints

Results on various biological outcomes among PLHIV following vaccination were available from two RCTs with placebo arms,^71^^,^^64^ one RCT comparing the bivalent and quadrivalent vaccine,^31^ two trials with historical controls^46^^,^^55^ and three single arm trials without control,^32^^,^^82^^,^^79^ Studies were conducted among women (*N_s_*=2), men (*N_s_*=3), or both (*N_s_*=3), and among study participants HPV negative at baseline (*N_s_*=7) or with unknown baseline HPV status (*N_s_*=3). (Supplement Table S6; additional details in S7). No results were available for children. No results by HIV disease stage or ART status were available. Estimates of biological outcomes were generally deemed of low quality following our study quality assessment. Main study limitations included failure to specify the HPV vaccine type present in abnormal cytology samples, define baseline HPV DNA status, not reporting timing of measurement, and/or poor comparability of what with the control group. Furthermore, it was unclear if the analyses accounted for the time lag required to mount an effective immune response against infection following vaccination, and follow-up tended to be short compared to the development of most outcomes. Thus, it was unclear whether results reflected incident infection acquired after having developed a full vaccine immune response, an infection acquired before developing an immune response, or persisting prevalent infections present at baseline. Given the study quality limitations highlighted above and the variety of anogenital endpoints reported (e.g., infections, cytology results, and genital warts) at different time points following vaccination and the mixed, or lack of, information on past and current exposure status to HPV of study participant at baseline, it was not possible to pool results, and individual study results were difficult to interpret.

### Safety of vaccine(s)

Trials reported on the occurrence of AEs (*N_s_*=14), serious AEs (SAEs) (*N_s_*=14), and AEs that were possibly, probably, or definitely related to the vaccine (*N_s_*=5) (Supplement Table S7). Most results were for the quadrivalent vaccine (*N_s_*=12) and from trials conducted in the Americas (*N_s_*=6). AEs are events such as diarrhoea, injection site reactions or coughing, that are not categorized as ‘serious’ whereas SAEs are commonly defined as events that are life-threatening, require/extend hospitalization, or result in death.

All publications concluded that the bivalent, quadrivalent, or nonavalent vaccines were as well tolerated and safe for PLHIV, as for HIV-negative populations. Four out of five trials reported the occurrence of AEs that were possibly, probably, or definitely related to the vaccine in <0.5% of participants, whereas the fifth trial reported at least one of these AE in 49% (34/69) of participants.^56^ The difference was likely due to difference in definition as the first 4 trials only reported ≥grade 3 AEs whereas the latter included any AE.

Most trials reported between 0-7% (*N_s_*=13) of PLHIV receiving either the bivalent, quadrivalent or nonavalent vaccine experienced SAEs (Supplement Table S7B). In addition, one placebo-controlled RCT of the quadrivalent vaccine reported 12% (33/288) of SAEs in the vaccine arm but similar frequency 16% (46/287) in the placebo arm.^65^

## Discussion

Our review demonstrates that PLHIV develop a robust initial immune response following HPV vaccination and that these vaccines are safe. Seroconversion rates were high 28 weeks after receiving the first vaccine dose, exceeding 94% for HPV-16/18/6/11 for all three vaccines. Similar to HIV-negative people,^28^ seropositivity levels and antibody titers in PLHIV declined gradually over time, especially for HPV-18; a decline that was less marked for the bivalent than the quadrivalent vaccine. Administration of a fourth vaccine dose of the quadrivalent vaccine boosted antibody levels especially for HPV-18, but only 2 publications had long term results available.^63^^,^^66^ Although antibody levels tended to be slightly lower for adults, seroconversion rates were similar by sex or age groups in subgroup analyses. However, there was evidence of lower seropositivity levels and antibody levels in PLHIV than in HIV-negative people, and among PLHIV with low CD4 cell counts and/or not receiving ART,^82^^,^^53^^,^^54^ although the evidence was limited. Evidence of vaccine efficacy on biological outcomes following vaccination was generally of low quality, inconclusive and difficult to interpret even for the clinical trial with placebo since study limitations around HPV distribution, prior HPV exposure and cytological abnormalities present already at baseline in some instances.

Current WHO guidance on HPV vaccination recommends the administration of a 2-dose schedule of HPV vaccines in females under the age of 15 years.^86^ A 3-dose schedule is recommended for those ≥15 years, people who are immunocompromised and/or PLHIV.^86^ None of the studies included explored the effectiveness of a 2-dose schedule in PLHIV. Studies comparing a third and fourth dose schedule suggest that a fourth dose may help sustain a longer immune response among PLHIV.^63^^,^^67^ Although average antibody titers in PLHIV appeared slightly lower compared to HIV-negative people, they remained above the seropositivity cut-off level. The significance of these lower antibody levels remains ambiguous as levels required for clinical protection are currently unknown.^87^ The lack of a formal correlate of protection also hinders evaluation of the duration of protection and the possible need for a booster dose. One study in HIV-negative people^87^ has shown that declining antibody levels for HPV-18 over time do not necessarily result in HPV-18 break-through infections, which could also be true for PLHIV or PLHIV treated sufficiently early with sufficiently reconstituted immunity and functionality.^88^ The nonavalent vaccine has a higher antigen concentration than the quadrivalent vaccine, and it was found non-inferior to the quadrivalent vaccine in the general population with a higher GMT titer achieved for HPV-18.^89^ Given the GMT titers in PLHIV decline slightly faster than in HIV-negative people, there may be added benefits achieved from the nonavalent vaccine in this population, which warrant further research.

Our review has some limitations, predominantly due to the low quality of studies available and the heterogeneity of outcomes. Few studies reported on the bivalent and nonavalent vaccines and no studies were included on the recently approved bivalent Cecolin vaccine, thus the evidence is strongest for the quadrivalent vaccine. The results may not be representative of all PLHIV since studies enrolled relatively healthy PLHIV. Although one study did not find any association between antibody titers after HPV vaccination and HIV disease stages determined by CD4 cell counts, possibly due to the extent of immune reconstitution in all CD4 groups over the course of the study,^62^ other immunogenicity studies reported higher seroconversion and antibody titers in PLHIV with higher CD4 cell counts,^51^, ^52^, ^53^, ^50^, ^54^, ^48^, ^49^ on ART,^52^^,^^53^ or virally supressed.^51^^,^^54^ These results are consistent with a recent study that reported a 19% reduction in CIN2+ rates due to vaccination in women aged 18-26 years without immunosuppression history but a smaller reduction of 4% in women with an immunosuppression history.^90^ More research is needed on the influence of prior HPV exposure and the role of immune suppression since both could reduce the effectiveness/efficacy and duration of protection with different dosing of the vaccine. Additional data on the short- and long-term immune response by HIV disease stages, timing of ART initiation, duration of HIV treatment, and viral suppression would be required to investigate this question and determine the extent to which PLHIV diagnosed and/or treated at different HIV disease stages can benefit from vaccination and for how long.^91^ The pooled seropositivity results, varied in the level of heterogeneity present. Our subgroup analyses did not find any evidence of a difference of immune response by age, sex, region, vaccine type and timing of measurement, and suggest that HPV vaccines produce a strong immune response across the strata analyzed. However, we also found that heterogeneity remained high in the subgroup analyses indicating unexplained variation. Residual heterogeneity could also be due to type of assays used, variation in unmeasured participant characteristics, variation in the underlying HIV disease stage (ART status, CD4 cell count) and remaining differences in the timing of measurement since broad categories were considered. As the study was done over several years, we did not initially consider registering the protocol it was rarely requested when we started the review. Although it may be deemed preferable to register a review protocol, we believe that this does not affect the quality of our review since we have described the methods and different stages of the review and conducted the review according to PRISMA reporting guidelines.

No strong conclusions on vaccine effectiveness in PLHIV could be reached; trials reporting on biological outcomes were scarce, reporting diverse and estimates of low quality. These trials were not designed to estimate efficacy as their primary outcome; few trials were designed as a multi-arm-controlled trial, had sufficient follow-up for some of the biological outcomes (e.g., CIN2+) to occur, and/or had sufficient sample size to assess an efficacy outcome. Most studies were conducted in the Americas or Europe, with only two trials conducted in Africa, where some of the highest prevalence of both HPV and HIV infections and most cervical cancer cases occur, highlighting important data gaps, which limit the interpretation of results for Sub-Saharan Africa.

Our systematic review and meta-analysis have several strengths. Our review included 43 publications on 18 independent trials updates and extends previous work by including 20 new publications and 2 new trials compared to a previous systematic review.^36^ We evaluated available evidence on several outcomes for all vaccine types. We reported updated pooled estimates of seropositivity and conducted subgroup and meta-regression analyses to assess heterogeneity by vaccination, participant, and study characteristics. Previous meta-analysis suggested HPV antibody seroconversion above 90% across HPV vaccine types. However, they did not differentiate between the vaccine used, number of doses, or the timing of the measurements.^33^^,^^36^ We stratified for these factors, which reduced heterogeneity across pooled studies, and allowed for an in-depth quantitative evaluation of the immune response of the HPV vaccines in PLHIV. We followed best practices in summarizing evidence reducing the risk of publication bias: we included unpublished results from trials registers, we derived measures ourselves based on the reported estimates when possible, and we evaluated sources of bias by estimating study quality.

Our results have implications for public health policy. Current evidence does not support the need for HIV testing prior to vaccination since the vaccine appears safe and immunogenic across age, sex and risk populations. PLHIV could benefit from vaccination, since PLHIV can elicit a robust immune response following vaccination, PLHIV have a high HPV disease burden, and that 90% of cervical and anal cancers are estimated to be due to HPV-16/18/33/45/52/58.^7^^,^^92^ Though more (higher quality) data is needed on the long-term efficacy of HPV vaccination in PLHIV, especially on HPV infection and diseases, current results suggest that PLHIV could benefit from being vaccinated against HPV even after acquiring HIV if they have not previously been offered the vaccine. More research is additionally needed to assess the extent to which existing HPV infections prior to vaccination may reduce the long-term efficacy of HPV vaccines among PLHIV who are highly burdened by HPV infection. A previous mathematical modeling study suggested that the nonavalent vaccine may reduce CIN2+ risk by 50-60% over four years in a cohort of WLHIV in South Africa.^93^ However more research is needed to evaluate the potential population-level impact of vaccinating PLHIV and determine the potential contribution of vaccinating PLHIV towards cervical cancer elimination and to determine the best age, disease stage, and HIV treatment status for vaccinating PLHIV. Furthermore, the best model of vaccine delivery (e.g., at ART clinics, or at the time of HIV diagnosis) needs to be explored to minimize the risk of HPV related cancer among PLHIV and to the resulting population-level impact in the wider population. PLHIV can develop a robust humoral immune response following HPV vaccination. The vaccine was found to be safe and well tolerated, with few serious adverse events. We identified important data gaps on vaccine effectiveness against HPV infections or related disease. Antibody titers and seropositivity declined over time, especially for HPV-18, and for the quadrivalent compared to bivalent vaccine, and more so for PLHIV than HIV-negative people. Given the lack of a formalized correlate of protection, additional data are needed to determine the effectiveness and duration of protection on clinical outcomes among PLHIV as well as the potential contribution of vaccinating PLHIV on the way towards cervical elimination goals. Though quality of studies reporting biological and clinical outcomes was limited, our results suggest that PLHIV who have not been vaccinated against HPV prior to acquiring HIV can still benefit from receiving the vaccine. This study adds to the evidence base that PLHIV who were not vaccinated before they acquired HIV infection generate an immune response to the vaccine and could benefit from HPV vaccination. However, the high prevalence of HPV in adult PLHIV, may mean the benefits of HPV vaccination could be lower in PLHIV but more evidence is needed to confirm this. Similar to recommendations for HPV vaccination of the general population, vaccination before sexual debut is important.

## Contributors

MCB and MMR conceptualised this review. MCB, MMR, and LS planned the analyses. LS, MEB and NS did the search and independently did the screening, extracted data and assessed study quality. LS, MEB and NS resolved any discrepancies in screening and data extraction with input from MCB and MMR. LS, MCB, and MMR designed the modified quality assessment, and LS and MMR independently conducted the quality assessment with input from MCB to resolve conflicts. LS performed statistical analyses and created figures with input from MCB and MMR. LS, MCB, and MMR interpreted the results and conceptualised the first draft of the review, written by LS, with further input from SD and PB. All authors (LS, MMR, NS, MEB, PB, MB, MMG, RVB, MD, PM, SD, MMC) substantially contributed to the interpretation of the results, drafting of the discussion and editing of the different drafts. All authors (LS, MMR, NS, MEB, PB, MB, MMG, RVB, MD, PM, SD, MMC) reviewed and approved the final version of the manuscript.

## Data sharing statement

Data extracted for this study are presented in the supplementary material. Further information on the data are available upon request to the shared first co-authors (LS, MMR).

## Declaration of interests

LS has received funding from Sanofi Pasteur/AstraZeneca outside of the submitted work. MMR reports funding from Charles A King Trust Postdoctoral Fellowship and Harvard Data Science Institute, and travel support to attend meetings for cervical cancer elimination from the WHO and Canadian Institute of Health Research, all outside of the submitted work. RVB received abstract and manuscript writing support from Regeneron Pharmaceuticals, outside the submitted work. MMG reports an investigator-sponsored research grant from Gilead Sciences Inc., and contractual arrangements from the *Institut national de santé publique du Québec* (INSPQ), the *Institut d'excellence en santé et services sociaux* (INESSS), the *World Health Organization*, and the *Joint United Nations Programme on HIV/AIDS* (UNAIDS), all outside of the submitted work. MB reports funding from Bill & Melinda Gates Foundation (grant number OPP48979), outside the submitted work.

## Appendix Supplementary materials

Image, application 1
