# Supplementary material for: Immunogenicity, safety, and efficacy of the HPV vaccines among people living with HIV: A systematic review and meta-analysis
Source: eClinicalMedicine. 2022 Aug 3;52:101585. doi: 10.1016/j.eclinm.2022.101585 (PMC9350866; doi:10.1016/j.eclinm.2022.101585)
Supplement: Supplementary file 1 [file mmc1.docx]

**Supplement material**

# Supplement table S1: Search terms. *“HIV” and “vaccination”* were used as index terms wherever available.

| OR | AND | OR | AND | *OR* |
| --- | --- | --- | --- | --- |
| *HIV*  *Human Immunodeficiency virus*  *“HIV”* |  | *HPV*  *Human Papilloma Virus*  *Cervical Cancer Precursors*  *Cervical Cancer*  *Cervical lesions*  *Cervical Intraepithelial Neoplasm*  *High grade cervical abnormalities* |  | *Quadrivalent*  *Bivalent*  *Nonavalent*  *Gardasil*  *Cervarix*  *Vaccin** |

**Supplement table S2A: Criteria used for the independent quality assessment of each immunogenicity outcome in each publication^*^ (seropositivity or antibody titer results)**

| **Indicators of potential study quality and sources of biases** | | | |
| --- | --- | --- | --- |
| *Study design* | | | |
| **1** | Objective | Was the study specifically designed to estimate the outcome of interest?  (YES if it was the primary outcome of the main study and NO if it was not the primary outcome or if not specified in publications or registered clinical trial website) | 1 star if YES  0 star if NO |
| **2** | Population | Were the inclusion criteria clearly defining which specific PLHIV population the authors were trying to recruit?  (YES if specify if risk group, sex, age group such as adults or children) | 1 star if YES  0 star if NO |
| *Representativeness of the sample* | | | |
| **3** | Study participants | Were the characteristics of study participants at baseline reported?  (Yes if the characteristics of study participants detailed, e.g sex, risk group, age, HIV/treatment status, and NO if one is missing) | 1 star if YES  0 star if NO |
| **4** | Recruitment | Were the HIV stages and treatment characteristics of the PLHIV recruited reported and representative of all PLHIV?  (NO if characteristics of PLHIV participants too narrow, e.g. if mainly included healthy PLHIV defined as >90% on ART OR 90% with high CD4 cell count (>350) OR >90% virally suppressed, or if HIV health status was not reported) | 1 star if YES  0 star if NO |
| **5** | Enrollment rate and loss-to-follow up | Was the enrollment and FUP rate adequate to prevent concerns about selection bias?  (YES if enrollment rate in the study AND the follow-up rate was at least 80% and NO if <80% enrollment rate OR <80% follow-up OR numbers not reported) | 1 star if YES  0 star if NO |
| *Misclassification bias* | | | |
| **6** | Exposure to intervention | Was previous exposure to HPV vaccination of participants assessed?  (YES if participants had never been vaccinated before and no if some were vaccinated or if vaccination status was not reported) | 1 star if YES  0 star if NO |
| **7** | Baseline assessment of outcome | Was exposure to the outcome of interest assessed at baseline?  (YES if HPV serostatus or GMT level at baseline was reported, no if not assessed or reported) | 1 star if YES  0 star if NO |
| **8** | Analysis by previous exposure to outcome | Was baseline exposure to the outcome of interest taken accounted in the analysis?  (YES if the results are reported by serostatus and include results for baseline seronegative participants , and NO if results only included people previously exposed or if status is unclear/not reported) | 1 star if YES  0 star if NO |
| *Measurement error* | | | |
| **9** | Sample size | Was the sample size sufficient to precisely measure the outcome?  (YES if sample size justified or if it included at least 100 participants in total) | 1 star if YES  0 star if NO |
| **10** | Measurement methods | Was the study using methods to adequately measure outcomes?  (YES if the biological assay used was specified) | 1 star if YES  0 star if NO |
| **11** | Specificity of measurement | Was the outcome of interest specifically measured?  (Yes if the outcome measured was specific to the HPV types included in the vaccine evaluated, e.g. antibody to individual HPV DNA types included in vaccine tested) | 1 star if YES  0 star if NO |
| **12** | Reporting | Was the reporting of the main outcome unambiguous?  (NO if cut-off for seropositivity was not reported (only for seropositivity results), timing of measurement not mentioned or numerator/denominator was not available) | 1 star if YES  0 star if NO |
| **Maximum score:** 12 stars | | | |

* if the publication did not contain the required information the trial website was consulted.

**Supplement table S2B: Criteria used for the independent quality assessment of each biological endpoint in each publication^*^(e.g. anogenital DNA or cytology)**

| **Indicators of potential study quality and sources of biases** | | | |
| --- | --- | --- | --- |
| *Study design* | | | |
| **1** | Objective | Was the study specifically designed to estimate the outcome of interest?  (YES if the study was designed to estimate vaccine efficacy/effectiveness on biological outcomes) | 1 star if YES  0 star if NO |
| **2** | Population | Were the inclusion criteria clearly defining which specific PLHIV population the authors were trying to recruit?  (YES if specify if risk group, sex, age group) | 1 star if YES  0 star if NO |
| *Representativeness of the sample* | | | |
| **3** | Study participants | Were the characteristics of study participants at baseline reported?  (Yes if the characteristics of study participants detailed, e.g sex, risk group, age, HIV/treatment status, and NO if one is missing) | 1 star if YES  0 star if NO |
| **4** | Recruitment | Was the participant recruited representative of all PLHIV?  (NO if characteristics of PLHIV participants too narrow, e.g. if mainly included healthy PLHIV defined as >90% on ART OR 90% with high CD4 cell count (>350) OR >90% virally suppressed, or if HIV health status was not reported) | 1 star if YES  0 star if NO |
| **5** | Enrollment rate and loss-to-follow up | Was the enrollemnt and FUP rate adequate to prevent concerns about selection bias?  (YES if enrollment rate in the study AND the follow-up rate was at least 80% and NO if <80% enrollment rate OR <80% follow-up OR numbers not reported) | 1 star if YES  0 star if NO |
| *Misclassification bias* | | | |
| **6** | Exposure to intervention | Was previous exposure to HPV vaccination of participants assessed?  (YES if participants had never been vaccinated before and no if some were or if it was not reported) | 1 star if YES  0 star if NO |
| **7** | Baseline assessment of outcome | Was exposure to the outcome of interest assessed at baseline?  (YES if baseline DNA HPV status and any other outcome included (e.g. cytology) were reported) | 1 star if YES  0 star if NO |
| **8** | Analysis by previous exposure to outcome | Was baseline exposure to the outcome (HPV DNA) of interest taken accounted in the analysis?  (YES if results are presented among negative population at baseline only, and NO if results include people previous exposed or if not reported) | 1 star if YES  0 star if NO |
| *Measurement error* | | | |
| **9** | Sample size | Was the sample size sufficient to precisely measure the outcome?  (YES if sample size calculation was justified or sample size was at least 500 in total, including intervention and control) | 1 star if YES  0 star if NO |
| **10** | Measurement methods | Was the study using methods to adequately measure outcomes?  (YES if the biological or clinical methods used to confirm the endpoint was specified) | 1 star if YES  0 star if NO |
| **11** | Specificity of measurement | Was the biological outcome of interest clearly and specifically measured?  (YES if the outcome measured was specific to the HPV types included in the vaccine evaluated, e.g. vt-HPV DNA presents) | 1 star if YES  0 star if NO |
| **12** | Follow-up duration | Was the follow-up duration sufficient long for the biological outcomes to occur and be observed?  (YES if follow up of at least one year for cytology results and warts and 28 weeks following the last vaccine dose for anogenital HPV DNA detection). | 1 star if YES  0 star if NO |
| *Internal validity* | | | |
| **13** | Comparison group | Did the study include a contemporaneous control group of unvaccinated PLHIV?  (NO if single arm trial or if a historical control group was included but sampled in a different year, country or study population, or risk group only and YES if the control group or the HC was sampled from the same country or study population/risk group and same year, or risk group) | 1 star if YES  0 star if NO |
| **14** | Comparability | Was the trial a randomized control trial?  (YES for randomized control trials and no otherwise) | 1 star if YES  0 star if NO |
| **15** | Confounding bias | Were the study participants between arms comparable?  YES if RCT or if they demonstrated that the characteristics of study participants (risk, sex and HIV clinical stage) were similar between groups and/or they performed an adjusted analysis and NO otherwise) | 1 star if YES  0 star if NO |
| **16** | Reporting | Was the reporting of the main outcome unambiguous?  (NO if cut-off for seropositivity was not reported (only for seropositivity results), timing of measurement not mentioned or numerator/denominator was not available) | 1 star if YES  0 star if NO |
| **Maximum score:** 16 stars | | | |

* if the publication did not contain the required information the trial website was consulted

**Summary of publications**

Number of publications (N_p_) (Supplement table S3):

Of these publications 22 emerged from the Americas and 10 from Europe, they evaluated the quadrivalent (N_p_=38), bivalent (N_p_=9) or nonavalent (N_p_=2) vaccines, including a three dose (N_p_=42) and/or four dose regimen(N_p_=6). These publications were among women only (N_p_=19), men only (N_p_=8) or both (N_p_=16), and on children (N_p_=9), adults (N_p_=20) or a mix (N_p_=140) The majority of publications reported immunogenicity results (seropositivity: N_p_=30; antibody titers: N_p_=23) and safety results (N_p_=36) with a few reporting on biological endpoints following vaccination (N_p_=11)

Of the controlled trials (N_p_= 21), 17 were RCTs and 4 were non-RCT. These publications compared the quadrivalent vaccine (N_p_=7) or the bivalent vaccine (N_p_=2) to a placebo, comparing the bivalent and quadrivalent vaccines (N_p_=6), or 3 and 4 doses (N_p_=2). Four of the RCTs included one or more non-randomized arms by including a HIV negative population^1–4^. The four publication on non-RCTs are all comparing a contemporary HIV negative and HIV positive population, one using the quadrivalent, one using the bivalent and two using the nonavalent vaccine. Two publication on RCTs included a historical control which was an HIV negative population. The remaining publications were from single arm trials (N_p_=22), of these publications 8 included a historical control of an HIV- population.

**Supplement table S3: Summary of all included publications on HPV type specific outcomes (N_p_= 43) by region and trials (N_s_=18); 8 of which were controlled trials and 10 were single arm trials (longitudinal studies).** Abbreviations: ART = antiretroviral, BC = baseline criteria, CIN = cervical intraepithelial neoplasia, (C)LIA = (chemi) luminescent immunoassay, EC = eligibility criteria, GMTs = geometric mean antibody titer, HAART = highly active antiretroviral theraphy, (HG)AIN = (high grade) anal intraepithelial neoplasia, HIV = human immunodeficiency virus, HPV = human papillomavirus, HSIL = high grade squamous intraepithelial lesion, MSM = men who have sex with men, NA = not available, RCT = randomized control trials, SAE = serious adverse events, SOT = solid organ transplant and qHPV = quadrivalent HPV vaccine, bHPV = bivalent HPV vaccine, 9vHPV = nonavalent vaccine.

| **Study design** | **Reference** | **Location**  **(study date)** | **Vaccine** | **Sample size**  **(duration of follow-up)** | **Study population**  **- HIV and treatment status*** | **Study arms** | **Stratification/comparison** | **Outcomes** | **HPV seropositivity results following vaccination**  **(in vaccinated group)** | **Stratified seropositivity results following vaccination**  **(in vaccinated group)** | **Conclusion** | |
| --- | --- | --- | --- | --- | --- | --- | --- | --- | --- | --- | --- | --- |
| **Americas** | | | | | | | | | | | |  |
| **Trial: NCT00339040 (NIAID)/ IMPAACT P1047** | | | | | | | | | | | |  |
| RCT | 2012^5^ | United States & Puerto Rico (2006 -2009) | qHPV | 126 | Boys and girls, children living with HIV, 7-12 years old  EC*: Baseline CD4% ≥ 15  BC*: 2 participants not on HAART; Mean CD4 cell count: 868 cells/μl; Mean CD4%: 34; 68% HIV RNA≤400 | 2 arms:  Placebo, qHPV | By study arm | Immunological (Seropositivity, GMTs) | *Seronegative at baseline^*^:*  HPV-6:  100% (94/94) at 28 weeks  94% (81/86) at 96 weeks  100% (81/81) at 100 weeks  HPV-11:  100% (94/94) at 28 weeks  96% (83/86) at 96 weeks  100% (81/81) at 100 weeks  HPV-16:  100% (94/94) at 28 weeks  99% (85/86) at 96 weeks  100% (81/81) at 100 weeks  HPV-18:  97% (91/94) at 28 weeks  76% (65/86) at 96 weeks  96% (78/81) at 100 weeks | N/A | The vaccine generated robust immune response that lasted for at least 72 weeks. Antibody responses for HPV 18 were lower compared to other qHPV types at 72 weeks. | |
|  | Levin 2010^6^ | United States & Puerto Rico (2006 -2009) | qHPV | 84 | Boys and girls, children living with HIV, 7-12 years old  EC*: Baseline CD4% ≥ 15 & ≥3 months of  HAART for  subjects with CD4% <25  BC*:  1% CD4<200 cells/μl;  32% HIV RNA≤400 and 16% >5000 copies/ml;Mean CD4%: 34; Mean CD4 cells counts: 868 | 2 arms: Placebo,  qHPV | By study arm,  by CD4 count  also  compared to historical controls (HIV uninfected, HPV vaccinated children) | Safety (Adverse events, grade events) Immunological (Seropositivity, GMTs) | N/A | *Seronegative at baseline:*  ***At* 28 weeks:**  **CD4% Nadir < 15 and CD4% ≥ 15:**  HPV-6:  100% (29/29)  HPV-11:  100% (30/30)  HPV-16:  100% (29/29),  HPV-18:  90% (27/30)  **CD4% Nadir ≥ 15 and CD4% ≥ 15 and <25:**  HPV-6:  100% (29/29)  HPV-11:  100% (30/30)  HPV-16:  100% (30/30)  HPV-18:  100% (30/30).  **CD4% Nadir ≥ 25 and CD4% ≥ 25:**  HPV-6:  100% (29/29)  HPV-18:  100% (30/30)  HPV-16:  100% (31/31)  HPV-18:  100% (30/30) | The vaccine was safe. It did not alter the participants' CD4 status or HIV viral load. Though most seroconverted, GMTs were lower compared to historical controls (uninfected), especially for HPV 18 and 6. | |
|  | Trial data published online^7^ | United States & Puerto Rico (2006 -2009) | qHPV | 130 | Boys and girls, children living with HIV, 7-12 years old  EC*:  CD4%≥15, for strata A and B use of HAART.  BC*: CD4 count strata A, B & C; 868, 1013 & 903. | 2 arms:  Placebo, qHPV | By study arm | Immunological (Seropositivity, GMTs) | *Seronegative at baseline^*^:*  **At 28 weeks:**  HPV-6:  100% (87/87),  HPV-11:  100% (90/90),  HPV-16:  100% (90/90),  HPV-18:  97% (87/90). | (Same as above) | N/A | |
| **Trial:** **NCT01206556 / IMPAACT P1085 (follow up: IMPAACT P1047)** | | | | | | | | | | | |  |
| RCT | Weinberg 2018^8^ | United States & Puerto Rico (2010 – 2013) | qHPV | 97 | Boys and girls, children living with HIV, 7-12 years old  EC*: Baseline CD4% ≥ 15 and had been on ART for ≥  3 months  BC*: 99% on ART;  1% CD4<200 cells/μl;  12% HIV RNA<400 and 15% >5000 copies/ml; Median CD4%: 35 | 2 arms:  3 and 4 doses of qHPV | By study arm | Immunological (IFN-y T-cell responses, IL2 T-cell responses) | N/A | N/A | IgG memory B and T-cell responses can be detected for up to 5 years post vaccination in HIV infected children. Whereas the memory responses remain stable in HIV uninfected women, they decrease in HIV infected populations between 2 and 5 years after the last dose of qHPV. No significant difference was found in B-cell memory between 3 and 4 dose immunization regimens. | |
|  | Levin 2017^9^ | United States & Puerto Rico (2010 – 2013) | qHPV | 97 | Boys and girls, children living with HIV, 7-12 years old  EC*: Baseline CD4% ≥ 15 and had been on ART for ≥  3 months  BC*: 99% on ART;  1% CD4<200 cells/μl;  12% HIV RNA<400 and 15% >5000 copies/ml; Median CD4%: 35 | 2 arms:  3 and 4 doses of qHPV | By study arm, by type of assay (cLIA/LIA) also  compared to  historical controls (HIV negative, HPV vaccinated children) | Immunological (GMTs, seropositivity) | *Seronegative at baseline^*^:*  **3 doses:**  HPV-6:  91% (20/22) at 96 weeks,  81% (17/21) at 168 weeks,  93% (13/14) at 216 weeks.  HPV-11:  95% (21/22) at 28 weeks,  81% (17/21) at 168 weeks,  86% (12/14) at 216 weeks.  HPV-16:  91% (20/22) at 96 weeks,  86% (18/21) at 168 weeks,  86% (12/14) at 216 weeks.  HPV-18:  55% (12/22) at 96 weeks,  52% (11/21) at 168 weeks,  64% (9/14) at 216 weeks.  **4 doses:**  HPV-6:  97% (71/73) at 96 weeks,  99% (68/69) at 168 weeks,  95% (55/58) at 216 weeks.  HPV-11:  97% (71/73) at 28 weeks,  99% (68/69) at 168 weeks,  98% (57/58) at 216 weeks.  HPV-16:  99% (72/73) at 96 weeks,  99% (68/69) at 168 weeks,  98% (57/58) at 216 weeks.  HPV-18:  81% (51/73) at 96 weeks,  77% (53/69) at 168 weeks,  74% (43/58) at 216 weeks.  **HIV-negative:**  **At 216 weeks:**  HPV-6:  94% (226/240),  HPV-11:  97% (233/240),  HPV-16:  99% (238/240),  HPV-18:  77% (185/240). | N/A | The group with 4 doses had significantly higher antibody titers at all study points (up to 5 years). Children with well-controlled HIV who receive 3 doses of qHPV remain seropositivity and antibody levels that are similar to children of the same age who are uninfected with HIV. Antibody titer was strongly correlated with low log HIV RNA, low CD8% and high CD4%. | |
| **Trial: NCT01461096**(NIAID) | | | | | | | | | | | |  |
| RCT | Wilkin 2018^10^ | United States, Brazil & Puerto Rico (2012 – 2013) | qHPV | 574 | Men and women, adults living with HIV, ≥  27 years old  BC*: 98% on ART; 86% HIV; RNA<200 and 6% ≥1000; Median CD4 598 copies/mL | 2 arms:  (qHPV and Placebo) | By study arm | Biological (Abnormal anal cytology, persistent oral or anal infection), Safety (serious) adverse events, safety grades) | N/A | N/A | HPV vaccination was safe and highly immunogenic. No evidence for the efficacy of HPV vaccination in preventing anal infection in HIV infected adults aged >= 27. | |
|  | Trial data published online^11^ | United States, Brazil & Puerto Rico (2012 – 2013) | qHPV | 575 | Men and women, Adults living with HIV, ≥  27 years old  BC*: 87% HIV RNA <200 and 6% ≥1000; Median CD4 602 copies/mL | 2 arms  (qHPV and Placebo) | By study arm | Biological (years to persistent anal qHPV infection, abnormal cytology) Safety (grade events and (S)AEs) | N/A | N/A | N/A | |
| **Trial****: NCT02236234 (University of Sao Paulo)** | | | | | | | | | | | |  |
| Controlled trial | Fontes 2016^12^ | Brazil (2012 – 2014) | bHPV | 30 | Men, adults living with HIV and HIV negative, 18-45 years old  BC*: 9/25 (16/25) CD4 cells > 500 (<500) cells/mL | 2 arms  : HIV negative and positive) | By study arm, by CD4 cell count (>500, <500)) | Immunological (Seropositivity) | No type specific outcome | N/A | The vaccine was effective and safe. Satisfactory response of HIV positive population to the vaccine was independent of the CD4 count group they were in. | |
| **Trial:** **NCT01209325** | | | | | | | | | | | |  |
| Single arm trial | Trial data published online^13^ (2011 – 2017) | USA & Puerto Rico | qHPV | 149 | Men, adults living with HIV, 18-27 years old  EC*: If receiving ART: at least for 3 months, no change in ART within 30 days prior to entry. Patients not on ART: CD4-cell count ≥ 350 cells/mm3 within 90 days prior to entry, no plans to start ART prior to Week 28 | 1 arm | N/A | Biological (Incidence of AIN or (peri)anal Condyloma or anogenital DNA infection) Immunological (GMTs), Safety (SEAs) | N/A | N/A | N/A | |
| **Trial: ISRCTN33674451** | | | | | | | | | | | |  |
| Single arm trial | McClymont 2020a^14^  (2008 - 2012) | Canada | qHPV | 284 | Women, children and adults living with HIV, ≥ 9 years old  BC*: CD4 count 499 cells/mL, HIV viral suppression (VL<50 copies/mL) 71%. | 1 arm | N/A | Biological (Incident persistent HPV infection) | N/A | N/A | Of the top 5 persistent HPV infections – 2 (52/45) are covered by the 9vHPV vaccine. Implying that the nonavalent vaccine would offer additional protection but gaps in protection would remain. | |
|  | McClymont 2020b^15^ (2008 - 2012) | Canada | qHPV | 307 | Women, children, and adults living with HIV, ≥ 9 years old  BC*: CD4 nadir 240 cells/mL; HIV viral load undetectable (<50 copies/mL) 66% | 1 arm | By dose spacing /time to complete the three doses (within 7 months,  within 7-12 months,  within 1-2 years) | Immunological (Peak median GMT titer) | N/A | N/A | Vaccine dose spacing did not appear to be associated with the peak antibody titer. | |
|  | McClymonth 2019^16^ (2008 - 2012) | Canada | qHPV | 279 | Women, children and adults living with HIV, 13-66 years old  BC*: 75% on ART; 69 % HIV viral load suppression (<50 copies/mL) 69%; Median CD4 500 copies/mL | 1 arm | Compared to historical controls (HIV negative vaccinated population, HIV negative placebo population, HIV positive placebo population) | Biological (Rates of persistent HPV, genital warts and CIN2+) | N/A | N/A | Vaccinated WLHIV may be at higher risk for vaccine failure than those that are HIV negative. However overall rates of vaccine failure were low, and rates of persistent qHPV were lower than in unvaccinated WLHIV. | |
|  | Brophy 2018^17^ (2008 - 2012) | Canada | qHPV | 35 | Girls, children living with HIV, 9-13 years old  BC*: 75% on ART; 59% viral load <50 copies/ml; Median CD4  692 cells/mm | 1 arm | Compared to historical controls (HIV negative girls, HPV vaccinated: 9-13 years old) | Immunological (Seropositivity, GMTs) Safety (adverse events) | *Seronegative at baseline^*^:*  **HIV-positive:**  HPV-6:  100% (32/32) at 28 weeks  88% (23/26) at 96 weeks  HPV-11:  100% (32/32) at 28 weeks  85% (22/26) at 96 weeks  HPV-16:  100% (32/32) at 28 weeks  100% (26/26) at 96 weeks  HPV-18:  100% (31/31) at 28 weeks  73% (19/26) at 96 weeks  **Historical controls (HIV-negative):**  HPV-6:  100% at 28 weeks  100% (187/187) at 96 weeks  HPV-11:  100% at 28 weeks  100% (187/187) at 96 weeks  HPV-16:  100% at 28 weeks  100% (187/187) at 96 weeks  HPV-18:  100% at 28 weeks,  94% (175/187) at 96 weeks. | N/A | Antibody response was lower in girls living with HIV relative to girls living without HIV. Virologic suppression predicted higher antibody titers (GMT titers not available) in multivariable regression models ( p<0.05 for all qHPV types). | |
|  | Money 2016^18^ (2008 - 2012) | Canada | qHPV | 296 | Women, children, and adults living with HIV, 15-66 years old  BC*: 72%  VL < 40 c/ml); Median CD4: 510 cells/mm3 | 1 arm | By HIV virological level (suppressed, unsuppressed)  Also compared to historical controls (HIV negative, HPV vaccinated women) | Immunological (Seropositivity, GMTs) Safety (adverse events) | *Seronegative at baseline^*^:*  HPV-6:  99% (101/102) at 28 weeks  93% (76/82) at 96 weeks  HPV-11:  99% (148/150) at 28 weeks  94% (106/113) at 96 weeks  HPV-16:  98% (106/108) at 28 weeks  98% (85/87) at 96 weeks  HPV-18:  94% (132/141) at 28 weeks  67% (71/106) at 96 weeks | N/A | Vaccine was well tolerated, and highly immunogenic. Suppressed viral load led to greater vaccine response. | |
|  | Trial data published online^19^ (2008 - 2012) | Canada | qHPV | 372 | Women, children, and adults living with HIV, ≥ 9 years old | 1 arm | N/A | Immunological (Seropositivity), Safety (adverse events) | (Same results as above) | N/A | N/A | |
| **Trial: NCT00513526 (AIDS Malignancy Consortium)** | | | | | | | | | | | |  |
| Single arm trial | Pinto 2019^20^ (2007-2011) | United States | qHPV | 225 | Men, adults,  Living with HIV, 22-61 years old  BC*: 84% on ART; Median CD4+: 498 cells/mm3, 81% with plasma  HIV-1 RNA <200 copies/mL | 1 arm | Also compared to historical controls (HIV negative, HPV vaccinated male: 27-45 years) | Immunological (GMTs, seropositivity) | *Mixed seropositivity at baseline^*^*  **HIV-positive:**  HPV-16:  39% (29/75) at 0 weeks  100% (75/75) at 28 weeks  96% (71/74) at 72 weeks  HPV-18:  40% (30/75) at 0 weeks  100% (75/75) at 28 weeks  84% (62/74) at 72 weeks  **HIV-negative:**  HPV-16:  19% (24/126) at 0 weeks  100% (126/126) at 28 weeks  99% (103/104) at 72 weeks  HPV-18:  21% (26/126) at 0 weeks  100% (126/126) at 28 weeks  90% (94/104) at 72 weeks | N/A | A high percentage of both arms seroconverted. Albeit antibody levels being lower among HIV positives compared to HIV negative controls, GMT plateau levels at 18 months were comparable. Those that were seropositive at baseline achieve higher antibody titers. | |
|  | Ellsworth 2018^21^ (2007-2011) | United States | qHPV | 103 | Men, adults living with HIV, 22-61 years old  BC*: 84% on ART; Median CD4+: 564 cells/mm^3^, 83% with plasma  HIV-1 RNA <200 copies/mL | 1 arm | N/A | Immunological (GMTs, seropositivity) | *Mixed seropositivity at baseline^*^*  HPV-6:  39% (39/101) at 0 weeks  97% (99/102) at 28 weeks  99% (89/90) at 76 weeks  95% (69/73) at 128 weeks  96% (64/67) at 129 weeks  100% (69/69) at 132 weeks  HPV-11:  30% (30/101) at 0 weeks  98% (100/102) at 28 weeks  98% (88/90) at 76 weeks  96% (70/73) at 128 weeks  100% (67/67) at 129 weeks  100% (69/69) at 132 weeks  HPV-16:  26% (26/101) at 0 weeks  99% (101/102) at 28 weeks  97% (87/90) at 76 weeks  95% (69/73) at 128 weeks  99% (66/67) at 129 weeks  100% (69/69) at 132 weeks  HPV-18:  18% (18/101) at 0 weeks  96% (98/102) at 28 weeks  67% (60/90) at 76 weeks  63% (46/73) at 128 weeks  91% (61/67) at 129 weeks  94% (65/69) at 132 weeks | N/A | Titer levels plateau at lower levels than seen in previous HIV uninfected population (3 doses). Rapid increase in titers 4 weeks after 4th dose. GMT for HPV 18 is lower, and is more likely to decline compared to other types. | |
|  | Wilkin 2010^22^ (2007-2011) | United States | qHPV | 112 | Men, adults living with HIV, 22-61 years old  EC*: If on ART: 6 months on ART+ HIV-1 RNA< 200 copies/mL+ CD4>350 cells/mL; If not on ART: CD4>350 cells/mL and staying off ART during study  BC*: 85% on ART; Median CD4+: 517 cells/mL, 83% with plasma  HIV-1 RNA <200 and 8% with> 10,000 copies/mL | 1 arm | By baseline HPV status (Anal HPV DNA)  By baseline HPV  serological status | Biological (Persistent & incident HPV infection, HGAIN or HSIL) Safety ( grade safety events), immunological (seropositivity, GMTs) | *Seronegative at baseline^*^:*  **At 28 weeks:**  HPV-6:  98% (61/62)  HPV-11:  97% (69/71)  HPV-16:  99% (74/75)  HPV-18:  95% (79/83) | N/A | The vaccine was safe and highly immunogenic in HIV-1 infected men. No serious adverse events were attributable to the vaccine. | |
|  | Trial data published online ^23^ (2007-2011) | United States | qHPV | 112 | Men, adults living with HIV, 22-61 years old  EC*: If on ART: 6 months on ART+ HIV-1 RNA< 200 copies/mL+ CD4>350 cells/mL; If not on ART: CD4>350 cells/mL and staying off ART during study | 1 arm | By baseline HPV status (Anal HPV DNA)  By baseline HPV  serological status | Immunological (Seropositivity, GMTs) | *Seronegative at baseline^*^:*  **At 28 weeks:**  HPV-6:  98% (59/60)  HPV-11:  99% (67/68)  HPV-16:  100% (62/62)  HPV-18:  95% (73/77) | N/A | N/A | |
| **Trial: NCT00710593 (University of North Carolina)** | | | | | | | | | | | |  |
| Single arm trial | Kahn 2013^24^ (2008-2011) | United States & Puerto Rico | qHPV | 99 | Women, adults living with HIV, 16-23 years old  EC*: ART naïve or for less than 6 months or on ART for more than 6 months, RNA plasma viral load< 400 copies/mL  BC*: 30% on ART.  CD4+: Median of 613, 86% ≥ 350 CD4 and 1% < 200 CD4 cells/mL;  40% (11.1%) with plasma  HIV-1 RNA <400 (9,999) copies/mL | 1 arm | By HIV treatment status (ART, no ART)  Also compared to historical controls (HIV negative vaccinated women: 16-23 years old) | Immunological (GMTs, Seropositivity), Safety (symptoms and adverse events) | *Seronegative at baseline^*^:*  **At 28 weeks:**  HPV-6:  100% (31/31)  HPV-11:  98% (51/52)  HPV-16:  98% (41/42)  HPV-18:  95% (54/57) | *Seronegative at baseline^*^:*  **On ART**  HPV-6:  53% (16/30) at 0 weeks,  100% at 28 weeks,  100% at 48 weeks.  HPV-11:  33% (10/30) at 0 weeks,  100% at 28 weeks,  100% at 48 weeks.  HPV-16:  43% (13/30) at 0 weeks,  100% at 28 weeks,  100% at 48 weeks.  HPV-18:  33% (10/30) at 0 weeks,  100% at 28 weeks,  87% at 48 weeks.  **Not on ART**  HPV-6:  58% (40/60) at 0 weeks,  100% at 28 weeks,  100% at 48 weeks.  HPV-11:  32% (22/69) at 0 weeks,  97% at 28 weeks,  97% at 48 weeks.  HPV-16:  41% (28/69) at 0 weeks,  96% at 28 weeks,  96% at 48 weeks.  HPV-18:  17% (12/69) at 0 weeks,  92% at 28 weeks,  68% at 48 weeks.  **HIV-negative controls:**  **At 28 weeks:**  HPV-6:  100% (276/276).  HPV-11:  100% (276/276).  HPV-16:  100% (276/276).  HPV-18:  100% (276/276). | Immune responses were robust for those that were HPV negative at baseline. GMT and seroconversion of HIV infected women on ART did not differ significantly from HIV uninfected women (historical control). | |
|  | Kahn 2017^25^ (2008-2011) | United States & Puerto Rico | qHPV | 76 | Women, adults living with HIV, 18-23 years old  EC*: ART naïve or for less than 6 months or on ART for more than 6 months, RNA plasma viral load< 400 copies/mL  BC*: CD4+: 99%<350 cells/mm^3^; 38% HIV viral load  <400 copies/mL | 1 arm | By HIV treatment status (ART, no ART)  By type of assay (LIA, cLIA) | Immunological (Seropositivity, GMTs) | *Seronegative at baseline^*^:*  HPV-6:  100% (30/30) at 28 weeks  100% (29/29) at 48 weeks  HPV-11:  98% (49/50) at 28 weeks  98% (45/46) at 48 weeks  HPV-16:  98% (40/41) at 28 weeks  98% (39/40) at 48 weeks  HPV-18:  95% (52/55) at 28 weeks  73% (38/52) at 48 weeks | *Seronegative at baseline^*^:*  **On ART**  HPV-6:  100% (10/10) at 28 weeks  100% (9/9) at 48 weeks  HPV-11:  100% (16/16) at 28 weeks  94% (16/17) at 48 weeks  HPV-16:  100% (13/13) at 28 weeks  100% (14/14) at 48 weeks  HPV-18:  100% (16/16) at 28 weeks  74% (14/19) at 48 weeks  **Not on ART**  HPV-6:  100% (20/20) at 28 weeks  100% (20/20) at 48 weeks  HPV-11:  97% (33/34) at 28 weeks  100% (29/29) at 48 weeks  HPV-16:  96% (27/28) at 28 weeks  96% (25/26) at 48 weeks  HPV-18:  92% (36/39) at 28 weeks  73% (24/33) at 48 weeks | cLIA measures lower seropositivity rates than LIA for HPV18 after 24 weeks. Seropositivity rates were higher in women with less advanced HIV disease. | |
|  | Trial data published online^26^ (2008-2011) | United States & Puerto Rico | qHPV | 99 | Women and children living with HIV, 16-23 years old  EC*: ART naïve or for less than 6 months or on ART for more than 6 months, RNA plasma viral load< 400 copies/mL | 1 arm | By HIV treatment status (ART, no ART)  Also compared to historical controls (HIV negative vaccinated women: 16-23 years old) | Immunological (GMTs, seroconversion) Safety (symptoms and adverse events) | *Seronegative at baseline^*^:*  **At 28 weeks:**  HPV-6:  98% (39/40)  HPV-11:  97% (61/63)  HPV-16:  96% (49/51)  HPV-18:  93% (62/67) | N/A | N/A | |
| **Region: Europe** | | | | | | | | | | | |  |
| **Trial: NCT01386164 (University of Aarhus)** | | | | | | | | | | | |  |
| RCT | Munk-Madsen  2018^27^ (2011-2013) | Denmark  (2011 - 2012) | bHPV & qHPV | 30 | Men or women, ART treated adults living with HIV, ≥ 18 years old  EC*: Include those with HIV RNA <200 copies/mL and exclude those on ART with >200 copies/mL  BC*: 100% on ART; Median CD4: 615 cells/μL; median HIV RNA level:19 copies/mL | 2 arms - bHPV & qHPV | By study arm, By baseline HPV status (Anal HPV DNA)  By baseline HPV  serological status | Immunological (GMTs, IL-2 & IFNy T cell response) | N/A | N/A | For both bHPV and qHPV, T cells increase significantly after vaccination in HIV infected adults. Similarly, as in HIV negative adults bHPV vaccine appears to induce higher anti HPV 18 antibodies. This was not the case for HPV 16. | |
|  | Faust 2016^28^ (2011-2013) | Denmark | bHPV & qHPV | 91 | Men or women living with HIV, ≥ 18 years old  EC*: As in Toft 2014  or BC*: NA | 2 arms: bHPV & qHPV | By study arm, By sex, by site (Genital, non-genital)  By assay type (pseudo virion Luminex, neutralization assays) | Immunological (Seropositivity) | *Seronegative at baseline^*^:*  **At 48 weeks**:  **qHPV:**  HPV-6:  100% (46/46)  HPV-11:  94% (43/46)  HPV-16:  96% (44/46)  HPV-18:  73% (34/46)  **bHPV:**  HPV-16:  100% (45/45)  HPV-18:  100% (45/45) | N/A | This study shows how immunogenicity for different HPV types could be used as an endpoint in HIV vaccine research. | |
|  | Toft 2014a^29^ (2011-2013) | Denmark | bHPV & qHPV | 91 | Men or women living with HIV, ≥ 18 years old  EC*: Excluded those HIV RNA level  >200copies/mL if on ART  BC*: 88% on ART; Median CD4: 593 cells/μL; Median HIV RNA levels= 19 if on ART & 60, 255 cells/mL if off ART | 2 arms:  bHPV & qHPV | By study arm, By baseline type specific HPV DNA status (anogenital) | Biological (HPV DNA detection (persistent/incident) for HPV 16 & 18), Safety (adverse events and specific symptoms) | N/A | N/A | bHPV vaccine is more immunogenic than the qHPV in HIV infected women. This difference was less pronounced in men. | |
|  | Toft 2014b^30^ (2011-2013) | Denmark | bHPV & qHPV | 91 | Men or women living with HIV, ≥ 18 years old  NA | 2 arms: bHPV & qHPV | By study arm, By baseline HPV serostatus (for 31/33/45) | GMTs, seropositivity (all for 31/33/45), HPV DNA. | N/A | N/A | Both vaccines induced anti HPV 31,33 and 45 neutralizing antibodies in participants who were HPV negative. | |
| **Trial: ISRCTN14732216** | | | | | | | | | | | |  |
| RCT | Hidalgo-Tenorio 2021^31^ (2011 – 2017) | Spain | qHPv | 129 | MSM, adults living with HIV, ≥ 18 years old  BC*: 92% on ART; Mean CD4 722 cells/ μL, 3.3%  virological failure | 2 arms: qHPV & Placebo | By study arms | Anal cytology & HPV DNA infection | N/A | N/A | No statistically significant differences were found in the onset of => HSIL or condylomas or the acquisition of anal HPV infection. Exception: HPV 6 at 12 months. | |
|  | Hidalgo-Tenorio 2017^32^ (2011 – 2017) | Spain | qHPV | 162 | MSM, adults living with HIV, ≥ 18 years old  BC*: 91% on ART; Mean CD4: 688 cells/μL; Mean RNA level: 5495 cells/ μL, 3.3%  virological failure | 2 arms: qHPV & Placebo | N/A | Safety (Adverse events & specific symptoms) | N/A | N/A | Significantly higher high risk antibody titers in vaccinated individuals. No serious adverse events. | |
| **Trial: NCT01512784** | | | | | | | | | | | |  |
| Controlled trial | Giacomet 2014^33^ (2011 – 2013) | Italy | qHPV | 92 | Men or women, children living with HIV and without HIV, 13–27 years old  EC*: Clinically asymptomatic, CD4+ count ≥350 cells/mm^3^, good HAART compliance & ≥2 HIV-RNA <37 copies/ml 6 months before enrolment  BC*: 100% on ART, Mean CD4 count: 715, CD4%: 34; 94%<37 copies/ml | 2 arms:  - HIV positive & HIV negative. | By study arm | Immunological (IgG titers, seroconversion) and safety (specific symptoms) | N/A | N/A | HPV antibody titers were slightly lower in the HIV positive group compared to the HIV negative one. Vaccine was safe and well tolerated. | |
| **Trial:** **NCT03525210** | | | | | | | | | | | |  |
| Controlled trial | Boey 2020^34^ (2018 – 2019) | Belgium | 9vHPV | 271 | Men, adults living with HIV or Solid Organ transplant (SOT) patients, 18-55 years old  EC*: CD4+ > 200 cells/μL at last check-up (<16 months ago)  BC*: 98% on ART, Median CD4: 737 cells/ cells/μL; RNA levels: 99% < 40 copies/mL | 2 arms:  HIV+  SOT patients | By study arm | Immunological (Seroconversion, GMTs), Safety (safety grades, adverse events & symptoms) | *Seronegative at baseline^*^:*  **At 28 weeks**:  **HIV-positive:**  HPV-6:  100% (62/62)  HPV-11:  100% (76/76)  HPV-16:  100% (63/63)  HPV-18:  100% (67/67)  HPV-31:  100% (70/70)  HPV-33:  100% (73/73)  HPV- 45:  100% (77/77)  HPV-52:  100% (85/85)  HPV-58:  100% (70/70)  **SOT patients:**  HPV-6:  64% (90/140)  HPV-11:  71% (106/150)  HPV-16:  69% (103/149)  HPV-18:  52% (74/143)  HPV-31:  56% (85/152)  HPV-33:  67% (101/151)  HPV-45:  46% (69/150)  HPV-52:  65% (98/150)  HPV-58:  72% (108/150) | N/A | All HIV positive participants seroconverted, seroconversion was lower for SOT patients. The vaccine was safe and well tolerated. | |
| Trial data published online^35^ | Belgium | 9vHPV | 310 | Men, adults living with HIV or SOT |  | 2 arms: HIV+  SOT patients | By study arm | Immunological (Seroconversion), Safety | Same as above. | N/A | N/A | |
| **Trial : N/A** | | | | | | | | | | | |  |
| Single arm trial | Oldakowska 2012^36^ (NA) | Poland | qHPV | 17 | Girls, children living with HIV, 10-18 years old  BC*: 100% on ART | 1 arm | N/A | Immunological (Seropositivity) | *HPV baseline seropositivity not specified.*  **At 72 weeks**:  HPV-6:  100% (17/17)  HPV-11:  100% (17/17)  HPV-16:  100% (17/17)  HPV-18:  100% (17/17) | N/A | Vaccine provided robust immune response. | |
| **Region: Africa** | | | | | | | | | | | | |
| **NCT00586339 (GlaxoSmithKline)** | | | | | | | | | | | | |
| Partially RCT | Denny 2013^37^ (2008 – 2011) | South Africa | bHPV | 150 | Women, living with and without HIV, adults, 18-25 years old  EC*: In WHO Clinical Stage 1, women on ART if compliant and viral load≤400 copies/mm^3^ for >6 months  BC: 2% on ART; 100% in Stage 1; Median CD4= 455 cells/mm^3^; Median HIV RNA levels: 23205 copies/ml | 3 arms: 2 randomised arms (HIV positive: bHPV, placebo) Non randomised arm (HIV negative: bHPV) | By study arms | Immunological (GMT, seropositivity) | **HIV-positive:**  HPV-16:  85% (52/61) at 0 weeks  100% (56/56) at 8 weeks  100% (42/42) at 48 weeks  HPV-18:  64% (39/61) at 0 weeks  100% (56/56) at 8 weeks  100% (42/42) at 48 weeks  **HIV-negative:**  HPV-16:  63% (19/30) at 0 weeks  100% (24/24) at 8 weeks  100% (22/22) at 48 weeks  HPV-18:  50% (15/30) at 0 weeks  100% (24/24) at 8 weeks  100% (22/22) at 48 weeks | N/A | Vaccine is well tolerated and immunogenic. The vaccine caused substantial increases in HPV16/18 antibodies which were sustained up to 12 months after vaccination. | |
|  | Trial data published online^4^ (2008 – 2011) | South Africa | bHPV | 150 | Women, adults living with HIV, 18-25 years old  EC*: subjects currently on ART must be compliant and have undetectable viral load. | 3- bHPV HIV positive & bHPV HIV negative & Placebo HPV positive | By study arms | Immunological (GMT, seropositivity), Safety (serious adverse events, specific symptoms) HIV viral load over time) | **HIV-negative:**  HPV-16:  78% (31/40) at 8 weeks  78% (31/40) at 28 weeks  76% (28/37) at 40 weeks  76% (28/37) at 48 weeks  HPV-18:  65% (26/40) at 8 weeks  63% (25/40) at 28 weeks  65% (24/37) at 40 weeks  65% (24/37) at 48 weeks | N/A | N/A | |
| **Trial: NCT00557245 (University of Washington)** | | | | | | | | | | | | |
| Single arm trial | Mugo 2021^38^ (2013 – 2015) | Kenya  (2013) | qHPV | 176 | Girls or boys, children living with HIV , 11-17 years old  complete | 1 arm | Compared to historical controls (HIV negative, HPV vaccinated girls, 9-15 years old) | Immunological (Seropositivity and GMTs) | *Seronegative at baseline^*^:*  HPV-6:  90% (155/172) at 96 weeks  86% (140/162) at 144 weeks  83% (132/159) at 192 weeks  HPV-11:  85% (147/172) at 96 weeks  83% (135/162) at 144 weeks  80% (127/159) at 192 weeks  HPV-16:  96% (165/172) at 96 weeks  93% (151/162) at 144 weeks  90% (143/159) at 192 weeks  HPV-18:  82% (141/172) at 96 weeks  78% (126/162) at 144 weeks  77% (122/159) at 96 weeks | N/A | For the majority of participants the antibody seropositivity remained above the cut off 48 months after the 1^st^ vaccine dose and is comparable to HIV negative control. Detectable HIV viral load appears to result in a less well-sustained antibody response. | |
|  | Mugo 2018^39^ (2013 – 2015) | Kenya | qHPV | 180 | Girls or boys, children living with HIV, 9-14 years old  EC*: Any CD4 counts or HIV treatment status  BC*: 86% on ART; Median CD4: 684 cell counts; RNA> 400c/ml: 28% if on ART; median RNA level 27,679 c/ml if not on ART | 1 arm | Compared to 3 historical controls (1: HIV positive, HPV vaccinated girls age 9-12 years, 2: HIV negative, HPV vaccinated girls age 9-15 in the USA,  3: HIV negative, HPV vaccinated women and girls 9–26-year-old in Sub-Saharan Africa) | Safety (Safety grades), Immunological (seropositivity and GMTs) | *Seronegative at baseline^*^:*  **At 28 weeks:**  **HIV-positive:**  HPV-6:  97% (172/178)  HPV-11:  97% (173/178)  HPV-16:  98% (175/178)  HPV-18:  93% (166/178)  **Control 1 (HIV-negative):**  HPV-6:  100% (87/87)  HPV-11:  100% (90/90)  HPV-16:  100% (90/90)  HPV-18:  97% (87/90).  **Control 2 (HIV-negative):**  HPV-6:  100% (147/147)  HPV-11:  100% (147/147)  HPV-16:  100% (160/160)  HPV-18:  100% (156/156)  **Control 3 (HIV-negative):**  HPV-6:  100% (916/917)  HPV-11:  100% (916/917)  HPV-16:  100% (914/915)  HPV-18:  100% (920/922) | N/A | Regardless of immune status all children experienced a substantial immune response with few AEs. | |
| **Region: Others** | | | | | | | | | | | | |
| **Trial: NCT01031069** | | | | | | | | | | | | |
| Partially RCT | Folschweiller 2020^2^ (2010 – 2015) | Brazil, Estoni, India & Thailand | bHPV & qHPV | 873 | Women and children living with and without HIV, 15-25-year-old  BC*: ART use 62%; Not detectable viral load (≤400 copies/mL), median CD4 count 587 cells/mm^3^ | 4 arms:  bHPV, qHPV in HIV positive and negative women | By study arms  B Baseline CD4 cells count (350-500,  >500) | Immunological (Seropositivity, GMTs), Safety (safety grades and symptoms) | *Seronegative at baseline^*^:*  **HIV-positive bHPV:**  HPV-16:  96% (84/88) at 6 weeks,  100% (88/88) at 10 weeks,  99% (83/84) at 28 weeks,  98% (83/85) at 48 weeks,  99% (83/84) at 72 weeks,  99% (80/81) at 96 weeks.  HPV-18:  96% (88/95) at 6 weeks,  100% (95/95) at 10 weeks,  99% (88/89) at 28 weeks,  99% (89/90) at 48 weeks,  89% (87/98) at 72 weeks,  95% (80/84) at 96 weeks.  **HIV-positive qHPV:**  HPV-16:  94% (86/92) at 6 weeks  100% (92/92) at 10 weeks  100% (88/88) at 28 weeks  98% (83/85) at 48 weeks  95% (81/85) at 72 weeks  96% (81/84) at 96 weeks  HPV-18:  66% (68/103) at 6 weeks  95% (99/104) at 10 weeks  97% (96/99) at 28 weeks  85% (82/96) at 48 weeks  68% (65/95) at 72 weeks  68% (65/95) at 96 weeks  **HIV-negative bHPV:**  HPV-16:  100% (118/118) at 6 weeks  100% (116/116) at 10 weeks  100% (98/98) at 28 weeks  100% (89/89) at 48 weeks  100% (93/93) at 72 weeks  100% (96/96) at 96 weeks  HPV-18:  100% (122/122) at 6 weeks  100% (120/120) at 10 weeks  100% (100/100) at 28 weeks  100% (90/90) at 48 weeks  100% (95/95) at 72 weeks  100% (88/88) at 96 weeks  **HIV-negative qHPV:**  HPV-16:  100% (125/125) at 6 weeks  100% (121/121) at 10 weeks  99% (105/106) at 28 weeks  99% (95/96) at 48 weeks  100% (101/101) at 72 weeks  100% (96/96) at 96 weeks  HPV-18:  90% (113/126) at 6 weeks  98% (121/123) at 10 weeks  98% (107/109) at 28 weeks  99% (98/99) at 48 weeks  98% (101/103) at 72 weeks  98% (96/98) at 96 weeks | *Seronegative at baseline^*^:*  **CD4 350-500, bHPV:**  HPV-16:  96% at 6 weeks,  100% at 10 weeks,  96% at 28 weeks,  96% at 48 weeks,  96% at 72 weeks,  96% at 96 weeks.  HPV-18:  93% at 6 weeks,  100% at 10 weeks,  97% at 28 weeks,  97% at 48 weeks,  100% at 72 weeks,  100% at 96 weeks.  **CD4 >500, bHPV:**  HPV-16:  95% at 6 weeks  100% at 10 weeks  100% at 28 weeks  98% at 48 weeks  96% at 72 weeks  96% at 96 weeks  HPV-18:  92% at 6 weeks,  100% at 10 weeks  100% at 28 weeks  96% at 48 weeks  98% at 72 weeks  96% at 96 weeks  **CD4 350-500, qHPV:**  HPV-16:  90% at 6 weeks  100% at 10 weeks  96% at 28 weeks  96% at 48 weeks  93% at 72 weeks  93% at 96 weeks  HPV-18:  55% at 6 weeks  90% at 10 weeks  97% at 28 weeks  76% at 48 weeks  46% at 72 weeks  48% at 96 weeks  **CD4 >500, qHPV:**  HPV-16:  95% at 6 weeks  100% at 10 weeks  100% at 28 weeks  98% at 48 weeks  97% at 72 weeks  98% at 96 weeks  HPV-18:  71% at 6 weeks  97% at 10 weeks  97% at 28 weeks  90% at 48 weeks  78% at 72 weeks  77% at 96 weeks | Though all participants seroconverted initially, bHPV proved immunological superiority to qHPV in WLHIV at 7 months – especially for HPV16 & 18. Antibody responses were lower in WLHIV compared to HIV negative women. Immunogenicity levels achieved for WLHIV receiving the bHPV vaccine were comparable to women without HIV receiving the qHPV vaccine. | |
|  | Trial data published online^40^ (2010 – 2015) | Brazil, Estonia, India & Thailand | bHPV & qHPV | 873 | Women and children living with and without HIV, 15–25-year-old  EC*: for HIV seropositive subjects – must be asymptomatic, CD4 count > 350 cells/mm3, if taking ART must be HAART and have undetectable viral load on two previous clinical visits. | 4 arms:  bHPV, qHPV in HIV positive and negative women | By study arms  B Baseline CD4 cells count (350-500,  >500) | Safety (Grade 3 solicited/unsolicited symptoms), Immunological (GMTs) | N/A | N/A | N/A | |
| **Trial:** **NCT00667563** | | | | | | | | | | | | |
| Single arm trial | Palefsky 2021^41^ (2009 – 2012) | India | qHPV | 150  BC*: median CD4 count 505 cells/mm^3^; median HIV copy 5600 copies/mL | Women, adults living with HIV, 21-41 years old  complete | 1 arm | N/A | Immunological (Seropositivity), Safety (safety grades) | *Seronegative at baseline.*  HPV-16:  100% (95/95) at 28 weeks  99% (96/97) at 52 weeks  HPV-18:  94% (91/97) at 28 weeks  84% (73/87) at 52 weeks | N/A | High seroconversion rates – as Indian women have relatively few sexual partners compared to other countries it might be beneficial to also vaccinate women above the age of 26. | |
|  | Trial data published online^42^ (2009 – 2012) | India | qHPV | 150  EC*: if receiving ART; Nadir CD4 ≤350 cells/mm^3^, if not receiving ART; ≥350 cells/mm^3^ | Women, adults living with HIV, 21-41 years old  complete | 1 arm | N/A | Safety (HIV viral load over time), Immunological (Seropositivity, (S)EAs) | *Seronegative at baseline^*^:*  **At 28 weeks:**  HPV-6:  100% (88/88)  HPV-11:  99% (110/111)  HPV-16:  99% (95/96)  HPV-18:  90% (94/104) | N/A | N/A | |
| **Trial: NCT00604175/ A5240 (NIAID)** | | | | | | | | | | | | |
| Single arm trial | Cespedes 2018^43^ (2008 – 2011) | United States, Brazil & South Africa  (March 2008 to July 2011) | qHPV | 315 | Women, children and adults living with HIV, 13–45-year-old  BC*: Median CD4 in strata A, B, C:  519, 287, 154  cells/mm^3^; undetectable HIV-1 RNA viral load (< 20 copies)  38%, 45%, and 38% | 1 arm | By CD4 cell count ( > 350, >200, ≤350, ≤200)  by baseline type specific HPV DNA status (cervix, anal) | Immunological (GMTs, seropositivity), biological (persistent HPV DNA detection) | *Seronegative at baseline^*^:*  **At 72 weeks:**  HPV-6:  85% (122/144)  HPV-11:  86% (173/201)  HPV-16:  90% (156/174)  HPV-18:  66% (115/175) | *Seronegative at baseline^*^:*  *.*  **CD4>350:**  **At 72 weeks:**  HPV-6:  94% (49/52)  HPV-11:  95% (76/80)  HPV-16:  95% (59/62)  HPV-18:  69% (47/68).  **CD4 201-350:**  **At 72 weeks:**  HPV-6:  85% (44/52)  HPV-11:  83% (52/63)  HPV-16:  88% (50/57)  HPV-18:  72% (43/60).  **CD4 <200:**  **At 72 weeks:**  HPV-6:  73% (29/40)  HPV-11:  78% (45/58)  HPV-16:  86% (47/55)  HPV-18:  53% (25/47) | Sustained antibody response one year post vaccination, decline over time is similar as that in healthy individuals. Sustained titer response was lower in people with detectable viral loads at baseline in the lowest CD4 stratum. | |
|  | Kojic 2014^44^ (2008 – 2011) | United States, Brazil & South Africa | qHPV | 319 | Women, children and adults living with HIV, 13–45-year-old  BC*: Median CD4: 310 cells/μL; 40% undetectable plasma HIV RNA (<25 copies/mL); HIV-1 RNA levels: 54%≤10,000, 50% ≤400 copies mL, 50% > 400 copies/mL | 1 arm | By CD4 cell count (> 350, >200, ≤350, ≤200)  by baseline type specific HPV DNA status (cervix, anal) | Immunological (Seropositivity and GMTs) | *Seronegative at baseline^*^:*  **At 28 weeks:**  HPV-6:  94% (140/149)  HPV-11:  96% (199/207)  HPV-16:  97% (173/179)  HPV-18:  84% (177/210) | *Seronegative at baseline^*^:*  **CD4>350:**  **At 28 weeks:**  HPV-6:  96% (52/54)  HPV-11:  98% (84/86)  HPV-16:  99% (66/67)  HPV-18:  91% (71/78).  **CD4 201-350:**  **At 28 weeks:**  HPV-6:  100% (50/50)  HPV-11:  98% (58/59)  HPV-16:  98% (55/56)  HPV-18:  85% (60/71)  **CD4 <200:**  **At 28 weeks:**  HPV-6:  84% (38/45)  HPV-11:  92% (57/62)  HPV-16:  93% (52/56)  HPV-18:  75% (46/61) | The vaccine was safe and immunogenic. Women with HIV RNA load > 10,000 and/or CD4 count <200 cells/ul had lower rates of seroconversion rates. | |
|  | Trial data published online^45^ (2008 – 2011) | United States, Brazil & South Africa | qHPV | 319 | Women, children and adults, 13–45-year-old | 1 arm | By CD4 cell count (> 350, >200, ≤350, ≤200)  by baseline type specific HPV DNA status (cervix, anal) | Immunological (Seropositivity, GMTs), Safety (serious adverse events and symptoms) | (Same as above) | (Same as above) | N/A | |

*Based on eligibility criteria (EC) or participant characteristics at baseline (BC) or during the study (SC) depending on information provided.

Supplement table S4A: HPV 16 – Subgroup analysis (age, sex, region and vaccine) of HPV seropositivity results among PLHIV who were HPV negative or positive at baseline– Shown are the pooled estimates of the proportion of participants that were seropositive and 95% confidence interval (95%CI). I2 displays the heterogeneity across estimates. Timing indicates the number of weeks since receiving the 1^st^ dose in the vaccination schedule. Adult = participants were ≥18 years old, child = participants were <18 years old, mix = a combination of adults and children. N_s_ = number of independent trial estimates included in the pooled estimate, bHPV = bivalent vaccine, qHPV = quadrivalent vaccine, 9vHPV = nonavalent vaccine. Pooled results for age, sex and region only included results for participants receiving the qHPV vaccine*.*

| Subgroup | Timing measurement (weeks) | Doses | Stratification | Pooled estimate | 95%CI | I^2^ | N_s_ | p-value |
| --- | --- | --- | --- | --- | --- | --- | --- | --- |
| Age group | 28 | 3 | Adult | 99% | 96% - 100% | 65% | 4 | 0.84 |
|  |  |  | Child | 100% | 96% - 100% | 76% | 2 |  |
|  |  |  | Mix | 99% | 96% - 100% | 52% | 3 |  |
|  | 29-99 | 3 | Adult | 96% | 90% - 99% | 80% | 4 | 0.72 |
|  |  |  | Child | 91% | 76% - 99% | NA | 1 |  |
|  |  |  | Mix | 96% | 93% - 99% | 0% | 2 |  |
|  |  | 4 | Child | 99% | 96% - 100% | 0% | 2 | - |
|  | ≥100 | 3 | Child | 86% | 68% - 97% | NA | 1 | - |
|  |  | 4 | Child | 100% | 97% - 100% | 54% | 2 | - |
| Sex | 28 | 3 | Both | 100% | 97% - 100% | 76% | 2 | 0.88 |
|  |  |  | Female | 99% | 97% - 100% | 64% | 6 |  |
|  |  |  | Male | 99% | 95% - 100% | NA | 1 |  |
|  | 29-99 | 3 | Both | 98% | 95% - 100% | 13% | 3 | 0.72 |
|  |  |  | Female | 97% | 95% - 99% | 57% | 5 |  |
|  |  | 4 | Both | 99% | 96% - 100% | 0% | 2 | - |
|  | ≥100 | 3 | Both | 86% | 68% - 97% | NA | 1 | - |
|  |  | 4 | Both | 100% | 97% - 100% | 54% | 2 | - |
| Region | 28 | 3 | Americas | 95% | 93% - 97% | 0% | 5 | 0.70 |
|  |  |  | Africa | 98% | 96% - 100% | NA | 1 |  |
|  |  |  | Asia | 99% | 96% - 100% | NA | 1 |  |
|  |  |  | Mix | 99% | 93% - 100% | 88% | 2 |  |
|  | 29-99 | 3 | Americas | 97% | 94% - 99% | 0% | 3 | 0.37 |
|  |  |  | Asia | 99% | 96% - 100% | NA | 1 |  |
|  |  |  | Europe | 96% | 88% - 100% | NA | 1 |  |
|  |  |  | Mix | 93% | 85% - 98% | 76% | 2 |  |
|  |  | 4 | Americas | 99% | 96% - 100% | 0% | 2 | - |
|  | ≥100 | 3 | Americas | 86% | 68% - 97% | NA | 1 | - |
|  |  | 4 | Americas | 100% | 97% - 100% | 54% | 2 | - |
| Vaccine | 28 | 3 | qHPV | 99% | 98% - 100% | 57% | 9 | 0.54 |
|  |  |  | bHPV | 99% | 95% - 100% | NA | 1 |  |
|  |  |  | 9vHPV | 100% | 98% - 100% | NA | 1 |  |
|  | 29-99 | 3 | qHPV | 96% | 92% - 98% | 64% | 7 | 0.06 |
|  |  |  | bHPV | 100% | 97% - 100% | 30% | 2 |  |
|  |  | 4 | qHPV | 99% | 96% - 100% | 0% | 2 | - |
|  | ≥100 | 3 | qHPV | 86% | 63% - 98% | NA | 1 | - |
|  |  | 4 | qHPV | 100% | 97% - 100% | 57% | 2 | - |

**Supplement t**able S4B: HPV 18 – Subgroup analysis (age, sex, region and vaccine) of HPV seropositivity results among PLHIV who were HPV negative or positive at baseline – Shown are the pooled estimates of the proportion of participants that were seropositive and 95% confidence interval (95%CI). I2 displays the heterogeneity across estimates. Timing indicates the number of weeks since receiving the 1^st^ dose in the vaccination schedule. Adult = participants were ≥18 years old, child = participants were <18 years old, mix = a combination of adults and children. N_s_ = number of independent trial estimates included in the pooled estimate, bHPV = bivalent vaccine, qHPV = quadrivalent vaccine, 9vHPV = nonavalent vaccine. Pooled results for age, sex and region only included results for participants receiving the qHPV vaccine.

| Subgroup | Timing measurement (weeks) | Doses | Stratification | Pooled estimate | 95%CI | I^2^ | N_s_ | p-value |
| --- | --- | --- | --- | --- | --- | --- | --- | --- |
| Age group | 28 | 3 | Adult | 99% | 97% - 100% | 54% | 5 | 0.81 |
|  |  |  | Child | 100% | 96% - 100% | 76% | 2 |  |
|  |  |  | Mix | 99% | 96% - 100% | 52% | 3 |  |
|  | 29-99 | 3 | Adult | 76% | 57% - 96% | 0.96 | 4 | 0.72 |
|  |  |  | Child | 67% | 47% - 87% | 0.70 | 2 |  |
|  |  |  | Mix | 73% | 61% - 85% | NA | 1 |  |
|  |  | 4 | Child | 78% | 71% - 84% | 0% | 2 | - |
|  | ≥100 | 3 | Child | 64% | 38% - 86% | NA | 1 | - |
|  |  | 4 | Child | 87% | 59% - 100% | 94% | 2 | - |
| Sex | 28 | 3 | Both | 95% | 91% - 98% | 41% | 2 | 0.80 |
|  |  |  | Female | 93% | 88% - 97% | 74% | 6 |  |
|  |  |  | Male | 95% | 90% - 99% | NA | 1 |  |
|  | 29-99 | 3 | Both | 65% | 46% - 82% | 59% | 2 | 0.35 |
|  |  |  | Female | 71% | 64% - 78% | 66% | 5 |  |
|  |  | 4 | Both | 78% | 71% - 84% | 0% | 2 | - |
|  | ≥100 | 3 | Both | 64% | 38% - 86% | NA | 1 | - |
|  |  | 4 | Both | 87% | 59% - 100% | 94% | 2 | - |
| Region | 28 | 3 | Americas | 95% | 93% - 97% | 0% | 5 | 0.79 |
|  |  |  | Africa | 93% | 89% - 96% | NA | 1 |  |
|  |  |  | Asia | 94% | 88% - 98% | NA | 1 |  |
|  |  |  | Mix | 92% | 75% - 100% | 93% | 2 |  |
|  | **29-99** | **3** | **Americas** | **67%** | **59% - 75%** | **15%** | **3** | **0.01** |
|  |  |  | **Asia** | **84%** | **76% - 91%** | **NA** | **1** |  |
|  |  |  | **Europe** | **74%** | **60% - 85%** | **NA** | **1** |  |
|  |  |  | **Mix** | **67%** | **61% - 72%** | **0%** | **2** |  |
|  |  | 4 | Americas | 78% | 71% - 84% | 0% | 2 | - |
|  | ≥100 | 3 | Americas | 64% | 38% - 86% | NA | 1 | - |
|  |  | 4 | Americas | 87% | 59% - 100% | 94% | 2 | - |
| Vaccine | **28** | **3** | **qHPV** | **94%** | **91% - 96%** | **67%** | **9** | **0.01** |
|  |  |  | **bHPV** | **99%** | **96% - 100%** | **NA** | **1** |  |
|  |  |  | **9vHPV** | **100%** | **99% - 100%** | **NA** | **1** |  |
|  | **29-99** | **3** | **qHPV** | **71%** | **64% - 76%** | **59%** | **7** | **<0.001** |
|  |  |  | **bHPV** | **96%** | **92% - 99%** | **0%** | **2** |  |
|  |  | 4 | qHPV | 78% | 71% - 84% | 0% | 2 | - |
|  | ≥100 | 3 | qHPV | 64% | 38% - 86% | NA | 1 | - |
|  |  | 4 | qHPV | 87% | 59% - 100% | 94% | 2 | - |

**Supplement** table S4C: HPV 6 – Subgroup analysis (age, sex, region and vaccine) of HPV seropositivity results among PLHIV who were HPV negative or positive at baseline – Shown are the pooled estimates of the proportion of participants that were seropositive and 95% confidence interval (95%CI). I2 displays the heterogeneity across estimates. Timing indicates the number of weeks since receiving the 1^st^ dose in the vaccination schedule. Adult = participants were ≥18 years old, child = participants were <18 years old, mix = a combination of adults and children. N_s_ = number of independent trial estimates included in the pooled estimate, bHPV = bivalent vaccine, qHPV = quadrivalent vaccine, 9vHPV = nonavalent vaccine. Pooled results for age, sex and region only included results for participants receiving the qHPV vaccine.

| Subgroup | Timing measurement (weeks) | Doses | Stratification | Pooled estimate | 95%CI | I^2^ | N_s_ | p-value |
| --- | --- | --- | --- | --- | --- | --- | --- | --- |
| Age group | 28 | 3 | Adult | 99% | 95% - 100% | 80% | 5 | 0.55 |
|  |  |  | Child | 99% | 93% - 100% | 88% | 2 |  |
|  |  |  | Mix | 100% | 98%-100% | 0% | 2 |  |
|  | 29-99 | 3 | Adult | 91% | 83% - 96% | 70% | 3 | 0.08 |
|  |  |  | Child | 91% | 76% - 99% | NA | 1 |  |
|  |  |  | Mix | 100% | 97% - 100% | NA | 1 |  |
|  |  | 4 | Child | 96% | 92% - 98% | 0% | 2 | - |
|  | ≥100 | 3 | Child | 81% | 62% - 94% | NA | 1 | - |
|  |  | 4 | Child | 100% | 97% - 100% | 54% | 2 | - |
| Sex | 28 | 3 | Both | 100% | 96% - 100% | 82% | 3 | 0.91 |
|  |  |  | Female | 99% | 96% - 100% | 78% | 5 |  |
|  |  |  | Male | 98% | 95% - 100% | NA | 1 |  |
|  | 29-99 | 3 | Both | 97% | 82% - 100% | 82% | 2 | 0.56 |
|  |  |  | Female | 94% | 82% - 100% | 88% | 3 |  |
|  |  | 4 | Both | 96% | 92% - 98% | 0% | 2 | - |
|  | ≥100 | 3 | Both | 81% | 62% - 94% | NA | 1 | - |
|  |  | 4 | Both | 100% | 97% - 100% | 54% | 2 | - |
| Region | **28** | **3** | **Americas** | **100%** | **99% - 100%** | **0%** | **5** | **<0.001** |
|  |  |  | **Africa** | **97%** | **93% - 99%** | **NA** | **1** |  |
|  |  |  | **Asia** | **100%** | **99% - 100%** | **NA** | **1** |  |
|  |  |  | **Mix** | **94%** | **90% - 97%** | **NA** | **1** |  |
|  | 29-99 | 3 | Americas | 96% | 86%- 100% | 72% | 3 | 0.15 |
|  |  |  | Europe | 100% | 99% - 100% | NA | 1 |  |
|  |  |  | Mix | 85% | 78% - 90% | NA | 1 |  |
|  |  |  | bHPV | 0% | 0% - 0% | NA | 1 |  |
|  |  | 4 | Americas | 96% | 92% - 98% | 0% | 2 | - |
|  | ≥100 | 3 | Americas | 81% | 62% - 94% | NA | 1 | - |
|  |  | 4 | Americas | 100% | 97% - 100% | 54% | 2 | - |
| Vaccine | 28 | 3 | qHPV | 99% | 97% - 99% | 42% | 8 | 0.42 |
|  |  |  | 9vHPV | 100% | 98% - 100% | NA | 1 |  |
|  | **29-99** | **3** | **qHPV** | **94%** | **86% - 99%** | **78%** | **5** | **< 0.001** |
|  |  |  | **bHPV** | **0%** | **0% - 0%** | **NA** | **1** |  |
|  |  | 4 | qHPV | 96% | 92% - 98% | 0% | 2 | - |
|  | ≥100 | 3 | qHPV | 93% | 74% - 100% | NA | 1 | - |
|  |  | 4 | qHPV | 99% | 89% - 100% | 86% | 2 | - |

**Supplement t**able S4D: HPV 11 – Subgroup analysis (age, sex, region and vaccine) of HPV seropositivity results among PLHIV who were HPV negative or positive at baseline – Shown are the pooled estimates of the proportion of participants that were seropositive and 95% confidence interval (95%CI). I2 displays the heterogeneity across estimates. Timing indicates the number of weeks since receiving the 1^st^ dose in the vaccination schedule. Adult = participants were ≥18 years old, child = participants were <18 years old, mix = a combination of adults and children. N_s_ = number of independent trial estimates included in the pooled estimate, bHPV = bivalent vaccine, qHPV = quadrivalent vaccine, 9vHPV = nonavalent vaccine. Pooled results for age, sex and region only included results for participants receiving the qHPV vaccine.

| Subgroup | Timing measurement (weeks) | Doses | Stratification | Pooled estimate | 95%CI | I^2^ | N_s_ | p-value |
| --- | --- | --- | --- | --- | --- | --- | --- | --- |
| Age group | 28 | 3 | Adult | 97% | 96% - 99% | 5% | 4 | 0.90 |
|  |  |  | Child | 97% | 93% - 99% | 0% | 2 |  |
|  |  |  | Mix | 98% | 87% - 100% | 92% | 2 |  |
|  | 29-99 | 3 | Adult | 99% | 96% - 100% | 56% | 3 | 0.90 |
|  |  |  | Child | 98% | 93% - 100% | NA | 1 |  |
|  |  |  | Mix | 98% | 24% - 100% | NA | 1 |  |
|  |  | 4 | Child | 96% | 71% - 100% | 97% | 2 | - |
|  | ≥100 | 3 | Child | 81% | 62% - 94% | NA | 1 | - |
|  |  | 4 | Child | 99% | 97% - 100% | 0% | 2 | - |
| Sex | 28 | 3 | Both | 100% | 97% - 100% | 80% | 3 | 0.33 |
|  |  |  | Female | 98% | 97% - 99% | 0% | 5 |  |
|  |  |  | Male | 97% | 93% - 100% | NA | 1 |  |
|  | 29-99 | 3 | Both | 99% | 91% - 100% | 64% | 2 | 0.13 |
|  |  |  | Female | 93% | 85% - 98% | 81% | 3 |  |
|  |  | 4 | Both | 96% | 71%-100% | 97% | 2 | - |
|  | ≥100 | 3 | Both | 81% | 62% - 94% | NA | 1 | - |
|  |  | 4 | Both | 99% | 97% - 100% | 0% | 2 | - |
| Region | 28 | 3 | Americas | 98% | 94% - 100% | 71% | 5 | 0.90 |
|  |  |  | Africa | 98% | 92% - 100% | NA | 1 |  |
|  |  |  | Asia | 99% | 97% - 100% | NA | 1 |  |
|  |  |  | Mix | 96% | 93% - 98% | NA | 1 |  |
|  | 29-99 | 3 | Americas | 99% | 97% - 100% | 0% | 3 | 0.12 |
|  |  |  | Europe | 100% | 98% - 100% | NA | 1 |  |
|  |  |  | Mix | 86% | 81% - 91% | NA | 1 |  |
|  |  | 4 | Americas | 96% | 71%-100% | 97% | 2 | - |
|  | ≥100 | 3 | Americas | 81% | 62% - 94% | NA | 1 | - |
|  |  | 4 | Americas | 99% | 97% - 100% | 0% | 2 | - |
| Vaccine | 28 | 3 | qHPV | 98% | 97% - 99% | 42% | 8 | 0.07 |
|  |  |  | 9vHPV | 100% | 99% - 100% | NA | 1 |  |
|  | **29-99** | **3** | **qHPV** | **96%** | **89% - 99%** | **85%** | **5** | **<0.001** |
|  |  |  | **bHPV** | **0%** | **0% - 0%** | **NA** | **1** |  |
|  |  | 4 | qHPV | 97% | 94% - 99% | 0% | 2 | - |
|  | ≥100 | 3 | qHPV | 86% | 63% - 98% | NA | 1 | - |
|  |  | 4 | qHPV | 100% | 96% - 100% | 57% | 2 | - |

**Supplement table S5: Ratio of geometric mean antibody titres 28 weeks after the first HPV vaccine dose was administered for studies comparing HIV positive populations to HIV negative populations**. All studies used a cLIA assay to measure antibody titers with the exception of Pinto 2019 using a VLP Elisa.

| **Reference** | **GMT in mMu/ml (range)** | | | | **Ratio**  **HIV+ vs HIV-** |
| --- | --- | --- | --- | --- | --- |
|  | **HIV positive** | | **HIV negative** | |  |
| **HPV-16** | | | | | |
| **Brophy 2018^c^** | 4,382 (3,045 – 6,305) | 7,640 (6,558 – 8,900) | | 0.6: 1 | |
| **Denny 2013** | 3,558 (2,724- 4,649) | 8,169 (6,341 – 10,524) | | 0.4: 1 | |
| **Mugo 2018** | 2,322 (1,912 – 2,820) | 4,919 (4,557 – 5,309)^a^ | | 0.5: 1 | |
| **Mugo 2018** | 2,322 (1,912 – 2,820) | 3,786 (3,360 – 4,265) ^b^ | | 0.6: 1 | |
| **Kahn 2013^c^** | 3,197 (1,581 – 4,813) | 3,892 (3,324 – 4,558) | | 0.8: 1 | |
| **Pinto 2019^c^** | 570 (422 – 770) | 2,072 (1,796 – 2,391) | | 0.4: 1 | |
| **HPV-18** | | | | | |
| **Brophy 2018^c^** | 640 (376 – 1,092) | 1,703 (1,479 – 1,960) | | 0.4: 1 | |
| **Denny 2013** | 1,946 (1,451 – 2609) | 3,703 (2503 – 5479) | | 0.5: 1 | |
| **Mugo 2018** | 364 (289 – 458) | 1,043 (968 – 1,123)^a^ | | 0.3: 1 | |
| **Mugo 2018** | 364 (289 – 458) | 811 (708 – 928) ^b^ | | 0.4: 1 | |
| **Kahn 2013^c^** | 619 (264 – 975) | 801 (694 – 925) | | 0.8: 1 | |
| **Pinto 2019^c^** | 173 (129 – 233) | 598 (504 – 710) | | 0.3: 1 | |
| **HPV-6** | | | | | |
| **Brophy 2018^c^** | 830 (567 – 1,214) | | 1,856 (1,582 – 2,178) | | 0.4: 1 |
| **Mugo 2018** | 356 (298 – 425) | | 929 (875 – 987)^a^ | | 0.4: 1 |
| **Mugo 2018** | 356 (298 – 425) | | 602 (526 – 689)^b^ | | 0.6: 1 |
| **Kahn 2013 ^c^** | 851 (319 – 1,382) | | 582 (527 – 643) | | 0.7: 1 |
| **HPV-11** | | | | | |
| **Brophy 2018^c^** | 977 (680 – 1,405) | 2,096 (1,870 – 2,349) | | 0.4: 1 | |
| **Mugo 2018** | 521 (433 – 625) | 1,305 (1,225 – 1,387)^a^ | | 0.4: 1 | |
| **Mugo 2018** | 521 (433 – 625) | 626 (545 – 718) ^b^ | | 0.8: 1 | |
| **Kahn 2013^c^** | 968 (497 – 1,439) | 697 (618 – 785) | | 1.4: 1 | |

**^a^** Mugo 2018 had two HIV negative historical controls; this one was conducted in Kenya among 9–15-year-old boys and girls

^b^ Mugo 2018 had two HIV negative historical controls; this one was conducted in Africa among 9–26-year-old boys, girls, men and women.

^c^ Both arms were HPV seronegative at baseline; for others baseline HPV status was unknown or participants were a mix of seropositive and seronegative.

**Table S6*:* Summary of results on biological outcomes amongst HIV positive populations vaccinated against HPV by trial type.** Additional details are presented in supplement table S6)*.*

| **Trial and reference** | **Study design (Study year)** | **Country** | **Population (baseline status)** | **Sample size**  **(N)** | **Biological outcomes**  **(timing)** | **Vaccine**  **arm** | **Estimates**  **Risk (%, n/N) or**  **Rate (per PY, 95%CI)** | **Risk/Rate ratio**  **(95%CI)** | **QS^\|\|^** | |
| --- | --- | --- | --- | --- | --- | --- | --- | --- | --- | --- |
| **A) Randomized controlled trials (N_s_= 3)** | | | | | | | | | |  |
| Toft 2014A & Toft 2014B  (NCT01386164)^29,30^ | RCT – 2 arms:  qHPV vs bHPV vaccines  (2011-2013) | Denmark | Both sex, adults  >18 years old (DNA: negative) | 91 (45 qHPV, 46 bHPV) | Anogenital HPV DNA  (at 28 weeks) | qHPV  bHPV | HPV-16 = 3.1% (1/32)  HPV-16 = 5.7% (2/35) | 0.6^ɣ^ (0.1 – 5.8)^##^  Ref: bHPV | 11 | |
|  |  |  |  |  |  | qHPV  bHPV | HPV-18 = 2.8% (1/36)  HPV-18 = 5.3% (2/38) | 0.5^ɣ^ (0.1 – 5.6)^##^  Ref: bHPV | 11 | |
| Wilkin 2018  (NCT01461096)^10^ | RCT – 2 arms:  qHPV vaccine vs placebo  (2012-2016) | United States & Brazil | Both sex, adults  >26 years old (DNA: negative, cytology: unkown; 30% of men  and 50% of women were required to have HSIL on histologic analysis of anal biopsies (bHSIL))) | 574 (288 qHPV, 286 Placebo) | Anal HPV DNA  (unknown) | qHPV  Placebo | Any qHPV HPV: 9.7% (28/288)  Any qHPV HPV: 14.3% (41/286) | 0.7^ɣ^ (0.4 – 1.1)^##,itt^  Ref: Placebo | 14 | |
|  |  |  |  |  | Oral HPV DNA (unknown) | qHPV  Placebo | Any qHPV HPV: 2.1 % (6/288)  Any qHPV HPV: 4.9% (14/286) | 0.4^ɣ^ (0.2 – 1.1)^##,itt^  Ref: Placebo | 13 | |
|  |  |  |  |  | Abnormal Anal Cytology  (at 52 weeks) | qHPV  Placebo | 53.2% (123/231)  52.8% (121/229) | 1.00 ^ɣ^ (0.82 – 1.23)^##,itt^  Ref: Placebo | 9 | |
|  |  |  |  |  | Abnormal Anal Cytology  (at 104 weeks) | qHPV  Placebo | 49.2% (98/199)  54.5% (108/198) | 0.9 ^ɣ^ (0.8 – 1.2)^##,itt^  Ref: Placebo | 9 | |
|  |  |  |  |  | Abnormal Anal Cytology  (at 156 weeks) | qHPV  Placebo | 44.6% (58/130)  54.5% (72/132) | 0.82 ^ɣ^ (0.66 – 1.16)^##,itt^  Ref: Placebo | 9 | |
| Hidalgo-Tenorio 2021  (ISRCTN14732216)^31^ | RCT – 2 arms:  qHPV vaccine vs placebo  (2011–2017) | Spain | MSM, adults, >26 years old (DNA: unknown) | 129 (66 qHPV, 63 Placebo) | Anal HPV DNA  (at 48 weeks) | qHPV  Placebo | HPV-6 = 7.5% (4/53)  HPV-6 = 23.4% (11/47) | 0.3^ɣ^ (0.1-0.9)***^##^  Ref: Placebo | 9 | |
|  |  |  |  |  |  | qHPV  Placebo | HPV-11 = 7.5% (4/53)  HPV-11 = 6.4% (3/47) | 1.2^ɣ^ (0.3 – 5.0)^##^  Ref: Placebo | 9 | |
|  |  |  |  |  |  | qHPV  Placebo | HPV-16 = 22.8% (12/53)  HPV-16 = 21.3% (10/47) | 1.1^ɣ^ (0.5 – 2.2)^##^  Ref: Placebo | 9 | |
|  |  |  |  |  |  | qHPV  Placebo | HPV-18 = 17.0% (9/53)  HPV-18 = 12.8% (6/47) | 1.3^ɣ^ (0.5 – 3.5)^##^  Ref: Placebo | 9 | |
|  |  |  |  |  | Anal HPV DNA  (at 96 weeks) | qHPV  Placebo | HPV-6 = 8.3% (4/48)  HPV-6 = 4.7% (2/43) | 1.8^ɣ^ (0.3 – 9.3)^##^  Ref: Placebo | 9 | |
|  |  |  |  |  |  | qHPV  Placebo | HPV-11 = 12.5% (6/48)  HPV-11 = 2.3% (1/43) | 5.4^ɣ^ (0.7 – 42.9)^##^  Ref: Placebo | 9 | |
|  |  |  |  |  |  | qHPV  Placebo | HPV-16 = 6.3% (3/48)  HPV-16 = 9.3% (4/43) | 0.7^ɣ^ (0.2 – 2.8)^##^  Ref: Placebo | 9 | |
|  |  |  |  |  |  | qHPV  Placebo | HPV-18 = 6.1% (3/48)  HPV-18 = 2.3% (1/43) | 2.7^ɣ^ (0.2-24.9) ^##^  Ref: Placebo | 9 | |
|  |  |  |  |  | Anal HPV DNA  (at 144 weeks) | qHPV  Placebo | HPV-6 = 2.7% (1/37)  HPV-6 = 8.8% (3/34) | 0.3^ɣ^ (0.0 – 2.8)^##^  Ref: Placebo | 9 | |
|  |  |  |  |  |  | qHPV  Placebo | HPV-11 = 5.4% (2/37)  HPV-11 = 0% (0/34) | -  - | 9 | |
|  |  |  |  |  |  | qHPV  Placebo | HPV-16 = 10.8% (4/37)  HPV-16 = 2.9% (1/34) | 3.7^ɣ^ (0.4 – 31.3)^##^  Ref: Placebo | 9 | |
|  |  |  |  |  |  | qHPV  Placebo | HPV-18 = 5.4% (2/37)  HPV-18 = 0% (0/34) | -  - | 9 | |
|  |  |  |  |  | Anal HPV DNA  (at 192 weeks) | qHPV  Placebo | HPV-6 = 10% (3/30)  HPV-6 = 3.7% (1/27) | 2.7^ɣ^  (0.3 – 24.4)^##^  Ref: Placebo | 9 | |
|  |  |  |  |  |  | qHPV  Placebo | HPV-11 = 0% (0/30)  HPV-11 = 0% (0/27) | -  - | 9 | |
|  |  |  |  |  |  | qHPV  Placebo | HPV-16 = 6.7% (2/30)  HPV-16 = 3.7% (1/27) | 1.8^ɣ^ (0.2 – 18.8)^##^  Ref: Placebo | 9 | |
|  |  |  |  |  |  | qHPV  Placebo | HPV-18 = 6.7% (2/30)  HPV-18 = 0% (0/27) | -  - | 9 | |
|  |  |  |  |  | Anal cytology  (at 48 weeks) | qHPV  Placebo | HSIL = 11.1% (7/63)  HSIL = 11.4% (7/61) | 1.0^ɣ^ (0.4 – 2.6)^##^  Ref: Placebo | 9 | |
|  |  |  |  |  | Anal cytology  (at 96 weeks) | qHPV  Placebo | HSIL = 0% (N/A)  HSIL = 2% (1/50) | 0.00 | 9 | |
|  |  |  |  |  | Anal cytology  (at 144 weeks) | qHPV  Placebo | HSIL = 1.9% (1/53)  HSIL = 0% (N/A) | -  - | 9 | |
|  |  |  |  |  | Anal cytology  (at 192 weeks) | qHPV  Placebo | HSIL = 2% (1/50)  HSIL = 0% (N/A) | -  - | 9 | |
|  |  |  |  |  | EAGL/Condyloma  (at 48 weeks) | qHPV  Placebo | 4.6% (3/65)  1.7% (1/63) | 2.9^ɣ^ (0.3 – 27.2)^##^  Ref: Placebo | 9 | |
|  |  |  |  |  | EAGL/Condyloma  (at 96 weeks) | qHPV  Placebo | 3.7% (2/54)  5.9% (3/51) | 0.6^ɣ^ (0.1 – 3.6)^##^  Ref: Placebo | 9 | |
|  |  |  |  |  | EAGL/Condyloma  (at 144 weeks) | qHPV  Placebo | 3.8% (2/52)  0% (0/N/A) | -  - | 9 | |
|  |  |  |  |  | EAGL/Condyloma  (at 192 weeks) | qHPV  Placebo | 0% (0/N/A)  0% (0/N/A) | -  - | 9 | |
| **B) Single arm trials – with historical controls (N_s_= 2)** | | | | | | | | | |  |
| McClymont 2019 & McClymont 2020a  (ISRCTN33674451)^14,16^ | Single arm trial (2008-2017)  Compared to 3 historical control groups  HC1: 2004 -2009  HC2-3: 1993-2002 | Canada | Trial: Women, children & adults, >9 years old (N/A)  HC1: HIV positive women (15–44 years), unvaccinated from Canada | Trial: 279  HC1= 750 | Newly acquired persistent qHPV DNA infection^a^  (timing unknown) | qHPV | 4.1% (11/268) | - | 9 | |
|  |  |  |  |  |  | qHPV  HC1 | 2.3 (95%CI: 1.1-4.1) per 100,000 PY  6.0 (95%CI: 4.6 – 7.7) per 100,000 PY | 0.38^o^ (N/A)^†##^  Ref: HC1 | 9 | |
|  |  |  |  |  | Genital warts  (timing unknown) | qHPV | 4% (11/264) | - | 7 | |
|  |  |  |  |  |  | qHPV  HC1 | 2.3 (95%CI: 1.2 – 4.1) per 100,000 PY  2.9 (95%CI: 2.1 – 3.9) per 100,000 PY | 0.79^o^ (N/A)^†##^  Ref: HC1 | 7 | |
|  |  |  |  |  | CIN2+  (timing unknown) | qHPV | 0.0% (0/217) | - | 7 | |
|  |  |  |  |  |  | qHPV  HC1 | 0.0 (95%CI: 0.0 – 0.9) per 100,000 PY  1.0 (95%CI: 0.5 – 1.9) per 100,000 PY | 0.0 (N/A)^†##^  Ref: HC1 | 7 | |
|  |  | HC2 & HC3: Colombia, France, Germany, Philippines, Spain, Thailand & USA | Trial: Women, adults, 24-45 years (negative)  HC2: qHPV  HC3: HIV negative women (24-55 years old) ,placebo | HC2 & HC3~1885 | Vaccine failure  [occurrence of either persistent qHPV infection, development of CIN2+ or genital wart]  (timing unknown)^a^ | qHPV  HC2  HC3 | 3.0 (95%CI: 1.4 -5.7) per 100,000 PY  2.7 (95%CI: 2.2-3.3) per 100,000 PY  (as above)  3.9 (95%CI: 3.3 – 4.6) per 100,000 PY | 1.1 (0.6-2.2)  Ref: HC2  0.8 (0.4-1.5)  Ref: HC3  0.7 (N/A)  Ref: HC2 vs HC3 | 7 | |
| Online trial results  (NCT01209325)^13^ | Single arm trial (2011-2017) vs 1 historical control group (HC, 2004-2009) | United States & Puerto Rico  HC: Australia, Brazil, Canada, Costa Rica, Croatia, Finland, Germany, Mexico, Netherlands, Norway, Peru, Philippines, Portugal, South Africa, Spain, Sweden, Taiwan, USA | MSM, adult, 18-27 years old (negative)  HC: HIV negative, boys and men (16-26), placebo | 144 | Incidence of AIN or Anal/Perianal Condyloma (between 28-48 weeks) | qHPV  HC | HPV-6 = 0 per 100 PY  HPV-6 = 11.1 per 100 PY | 0.00  Ref: HC | 7 | |
|  |  |  |  |  |  | qHPV  HC | HPV-11 = 0 per 100 PY  HPV-11 = 2.0 per 100 PY | 0.00  Ref: HC | 7 | |
|  |  |  |  |  |  | qHPV  HC | HPV-16 = 0 per 100 PY  HPV 16 = 1.8 per 100 PY | 0.00  Ref: HC | 7 | |
|  |  |  |  |  |  | qHPV  HC | HPV-18 = 0 per 100 PY  HPV-18 = 2.8 per 100 PY | 0.00  Ref: HC | 7 | |
|  |  |  |  |  | Incident persistent anogenital infection at 96 weeks | qHPV | HPV 6 = 1.8 per 100 PY  HPV 11 = 0 per 100 PY  HPV 16 = 2.9 per 100 PY  HPV 18 = 0.7 per 100 PY | - | 7 | |
|  |  |  |  |  | Incidence of penile/scrotal warts  (between 28-48 weeks) | qHPV | HPV-6 = 1.8 per 100 PY  HPV-11 = 0.9 per 100 PY  HPV-16 = 1.8 per 100 PY  HPV-18 = 0.7 per 100 PY | - | 7 | |
| **C) Single arm trials - without historical controls (N_s_= 3)** | | | | | | | | | |  |
| Cespedes 2018  (NCT00604175)^43^ | Single arm trial: qHPV vaccine (2008-2012) | United States, Brazil & South Africa | Both sex, adults and children, 13-45 years old (negative) | 315 | Cervical DNA  (at 28 weeks) | qHPV | HPV-6/11= 0.0% (0/224) | - | 10 | |
|  |  |  |  |  |  |  | HPV-16 = 1.8% (4/221) |  |  |  |
|  |  |  |  |  |  |  | HPV-18 = 0.4% (1/220) |  |  |  |
|  |  |  |  |  | Cervical DNA  (at 52 weeks) | qHPV | HPV-6/11 = 0.9% (2/224) | - | 10 | |
|  |  |  |  |  |  |  | HPV-16 = 2.3% (5/221) |  |  |  |
|  |  |  |  |  |  |  | HPV-18 = 0.0% (0/220) |  |  |  |
|  |  |  |  |  | Anal DNA  (at 28 weeks) | qHPV | HPV-6/11 = 1.8% (5/281) | - | 10 | |
|  |  |  |  |  |  |  | HPV-16 = 2.4% (5/210) |  |  |  |
|  |  |  |  |  |  |  | HPV-18 = 1.4% (3/218) |  |  |  |
|  |  |  |  |  | Anal DNA  (at 52 weeks) | qHPV | HPV-6/11 = 1.0% (3/281) | - | 10 | |
|  |  |  |  |  |  |  | HPV-16 = 1.9% (4/210) |  |  |  |
|  |  |  |  |  |  |  | HPV-18 = 2.3% (5/218) |  |  |  |
| Wilkin 2010  (NCT00513526)^22^ | Single arm trial:  qHPV vaccine (2007 – 2011) | United States | Men, adults, 22-61 years  old (negative) | 112 | Anal DNA  (at 28 weeks) | qHPV | HPV-6 = 5.5% (5/91)  HPV-11 = 3.3% (3/92)  HPV-16 = 2.5% (2/81)  HPV-18 = 1.1% (1/93) | - | 10 | |
|  |  |  |  |  | Anal Cytology  (at 28 weeks) | qHPV | ASCUS = 31.4% (33/105)  LSIL = 15.2% (16/105)  HSIL = 2.9% (3/105)  HGAIN = 11.4% (12/105) | - | 10 | |
| Palefsky 2021  (NCT00667563)^41^ | Single arm trial:  qHPV vaccine (2008-2016) | India | Women, adults, 27-38 years old (negative) | 150 | Cervical HPV DNA  (at 28 weeks) | qHPV | HPV-16 = 2% (2/95)  HPV-18 = 1% (1/97) | - | 9 | |
|  |  |  |  |  | Cervical HPV DNA  (at 52 weeks) | qHPV | HPV-16 = 0% (0/97)  HPV-18 = 2% (2/99) | - | 9 | |
| ^a^ Newly acquired persistent qHPV infection was defined as the detection of the same qHPV type in samples collected at 2 or more consecutive visits (>6 months apart) or detection of qHPV at the last available visit; ^##^ risk/rate ratios and 95%CIs were derived from available information; ^ɣ^ Risk ratio, ^o^ Rate Ratio; ^itt^ Intention to treat analysis; * test for significance, p-value < 0.05, † p-values were not calculated as no PY were available.  Abbreviations: HPV = Human Papillomavirus, HC = Historical control, qHPV = quadrivalent vaccine; bHPV = bivalent vaccine, NA = Not available, RCT = Randomized controlled trial, CIN = Cervical Intraepithelial Neoplasia, PY = person years; AIN = anal intraepithelial neoplasia; HSIL = high grade squamous intraepithelial lesion; EAGL = external ano-genital lesions; QS: quality score (number of points) based on tables S9. | | | | | | | | | |  |

**Supplement table S7: Full results (N_p_=6) on biological endpoints (e.g. anogenital HPV DNA or cytology) by baseline HPV DNA status. The difference tables is that S6 includes all arms presented in the publication.**

| Trial and reference | Study design (Study year) | Country | Population (baseline status) | Sample size  (N) | Biological outcomes  (timing) | Vaccine  arm | Estimates  Risk (%, n/N) or  Rate (per PY, 95%CI) | Risk/Rate ratio  (95%CI) | QS^\|\|^ |
| --- | --- | --- | --- | --- | --- | --- | --- | --- | --- |
| A) Randomized controlled trials (N_s_= 3) | | | | | | | | |  |
| Toft 2014A & Toft 2014B  (NCT01386164)^29,30^ | RCT – 2 arms:  qHPV vs bHPV vaccines  (2011-2013) | Denmark | Both sex, adults  >18 years old  (DNA: negative) | 91 (45 qHPV, 46 bHPV) | Anogenital HPV DNA  (at 28 weeks) | Per protocol:  bHPV  qHPV | HPV-16 = 5.7% (2/35)  HPV-18 = 5.3% (2/38)  HPV-16 = 3.1% (1/32)  HPV-18 =2.8% (1/36) | HPV 16 = 0.6^ɣ^ (0.1 – 5.8)^##^  HPV 18 = 0.5^ɣ^ (0.1 – 5.6)^##^  Ref: bHPV | 11 |
|  |  |  | (DNA: positive or negative) |  | Anogenital HPV DNA  (at 28 weeks) | Per protocol:  bHPV  qHPV | HPV-16 = 10.3% (4/39)  HPV-18 = 7.7% (3/39)  HPV-16 = 19.5% (8/41)  HPV-18 =9.8% (4/41) | HPV 16 = 1.9^ɣ^ (0.6 – 5.8)^##^  HPV 18 = 1.2^ɣ^ (0.3 – 5.3)^##^  Ref: bHPV | 11 |
|  |  |  | (DNA: positive) |  | Anogenital HPV DNA  (at 28 weeks) | Per protocol:  bHPV  qHPV | HPV-16 = 50.0% (2/4)  HPV-18 =50.0% (1/2)  HPV-16 = 77.8% (7/9)  HPV-18 =60.0% (3/5) | HPV 16 = 1.5^ɣ^ (0.5 – 4.4)^##^  HPV 18 = 1.2^ɣ^ (0.3 – 5.7)^##^  Ref: bHPV | 11 |
| Wilkin 2018  (NCT01461096)^10^ | RCT – 2 arms:  qHPV vaccine vs placebo  (2012-2016) | United States & Brazil | Both sex, adults  >26 years old ; 30% of men  and 50% of women were required to have HSIL on histologic analysis of anal biopsies (bHSIL)))  (DNA: negative, cytology: unkow) | 574 (288 qHPV, 286 Placebo) | Anal HPV DNA  (unknown) | Full ITT^a^:  Placebo  qHPV | Persistent infections^c^:  all qHPV=14.3% (41/286)  all qHPV=9.7% (28/288) | 0.7^ɣ^ (0.4 – 1.1)^##,itt^  Ref: placebo | 14 |
|  |  |  |  |  |  | Modified- ITT^b^:  Placebo  qHPV | Persistent infections^c^:  all qHPV = 6.0% (17/283)  all qHPV = 4.9% (14/286) | 0.8^ɣ^ (0.4 – 1.6)^##,itt^  Ref: placebo | 14 |
|  |  |  |  |  |  | Modified- ITT^b^:  Placebo  qHPV | Persistent infection^c^, including single detection at final visit:  all qHPV = 11.7% (33/283)  all qHPV = 9.4% (27/286) | 0.8^ɣ^ (0.5 -1.3)^##,itt^  Ref: placebo | 14 |
|  |  |  | (DNA: negative) |  | Anal HPV DNA  (unknown) | Per protocol^d^:  Placebo  qHPV | Persistent infections^c^:  all qHPV = 4% (10/277)  all qHPV = 3% (7/276) | 0.7 (0.3 – 1.8)  Ref: placebo | 14 |
|  |  |  | (DNA: negative) |  | Oral HPV DNA  (unknown) | Full ITT:  qHPV  Placebo | Persistent infections^c^:  all qHPV = 2.1 % (6/288)  all qHPV = 4.9% (14/286) | 0.44^ɣ^ (0.17–1.12)  Ref: placebo | 14 |
|  |  |  |  |  |  | Modified ITT^b^: qHPV  Placebo | Persistent infections^c^:  all qHPV = 0.3% (1/288)  all qHPV = 2.8% (8/286) | - 1. (0.0 – 1.0)*   Ref: placebo | 14 |
|  |  |  |  |  |  | Modified ITT^b^:  Placebo  qHPV | Persistent infection^c^, including single detection at final visit:  all qHPV = 3.5% (10/286)  all qHPV = 2.4% (7/288) | 0.7 (0.3 – 1.8)  Ref: placebo | 14 |
|  |  |  |  |  |  | Per protocol^d^:  Placebo  qHPV | Persistent infections^c^:  all qHPV = 1.1% (3/280)  all qHPV = 0.4% (1/278) | 0.3 (0.0 – 3.2)  Ref: placebo | 14 |
|  |  |  | (DNA: unknown) |  | Abnormal Anal Cytology  (52 weeks) | NA  qHPV  Placebo | Abnormal = 53.2% (123/231)  Abnormal = 52.8% (121/229) | 1.00 (0.82–1.23)  Ref | 9 |
|  |  |  |  |  | Abnormal Anal Cytology  (104 weeks) | NA  qHPV  Placebo | Abnormal = 49.2% (98/199)  Abnormal = 54.5% (108/198) | 0.9 (0.7 – 1.1)  Ref | 9 |
|  |  |  |  |  | Abnormal Anal Cytology  (156 weeks) | NA  qHPV  Placebo | Abnormal = 44.6% (58/130)  Abnormal = 54.5% (72/132) | 0.8 (0.6 – 1.0)  Ref | 9 |
| Hidalgo-Tenorio 2021  (ISRCTN14732216)^31^ | RCT – 2 arms:  qHPV vaccine vs placebo  (2011–2017) | Spain | MSM, adults, >26 years old (DNA: unknown) | 129 (66 qHPV, 63 Placebo) | Anal HPV DNA  (at 48 weeks) | qHPV  Placebo | HPV-6 = 7.5% (4/53)  HPV-6 = 23.4% (11/47) | 0.3^ɣ^ (0.1-0.9)***^##^  Ref: Placebo | 9 |
|  |  |  |  |  |  | qHPV  Placebo | HPV-11 = 7.5% (4/53)  HPV-11 = 6.4% (3/47) | 1.2^ɣ^ (0.3 – 5.0)^##^  Ref: Placebo | 9 |
|  |  |  |  |  |  | qHPV  Placebo | HPV-16 = 22.8% (12/53)  HPV-16 = 21.3% (10/47) | 1.1^ɣ^ (0.5 – 2.2)^##^  Ref: Placebo | 9 |
|  |  |  |  |  |  | qHPV  Placebo | HPV-18 = 17.0% (9/53)  HPV-18 = 12.8% (6/47) | 1.3^ɣ^ (0.5 – 3.5)^##^  Ref: Placebo | 9 |
|  |  |  |  |  | Anal HPV DNA  (at 96 weeks) | qHPV  Placebo | HPV-6 = 8.3% (4/48)  HPV-6 = 4.7% (2/43) | 1.8^ɣ^ (0.3 – 9.3)^##^  Ref: Placebo | 9 |
|  |  |  |  |  |  | qHPV  Placebo | HPV-11 = 12.5% (6/48)  HPV-11 = 2.3% (1/43) | 5.4^ɣ^ (0.7 – 42.9)^##^  Ref: Placebo | 9 |
|  |  |  |  |  |  | qHPV  Placebo | HPV-16 = 6.3% (3/48)  HPV-16 = 9.3% (4/43) | 0.7^ɣ^ (0.2 – 2.8)^##^  Ref: Placebo | 9 |
|  |  |  |  |  |  | qHPV  Placebo | HPV-18 = 6.1% (3/48)  HPV-18 = 2.3% (1/43) | 2.7^ɣ^ (0.2-24.9) ^##^  Ref: Placebo | 9 |
|  |  |  |  |  | Anal HPV DNA  (at 144 weeks) | qHPV  Placebo | HPV-6 = 2.7% (1/37)  HPV-6 = 8.8% (3/34) | 0.3^ɣ^ (0.0 – 2.8)^##^  Ref: Placebo | 9 |
|  |  |  |  |  |  | qHPV  Placebo | HPV-11 = 5.4% (2/37)  HPV-11 = 0% (0/34) | -  - | 9 |
|  |  |  |  |  |  | qHPV  Placebo | HPV-16 = 10.8% (4/37)  HPV-16 = 2.9% (1/34) | 3.7^ɣ^ (0.4 – 31.3)^##^  Ref: Placebo | 9 |
|  |  |  |  |  |  | qHPV  Placebo | HPV-18 = 5.4% (2/37)  HPV-18 = 0% (0/34) | -  - | 9 |
|  |  |  |  |  | Anal HPV DNA  (at 192 weeks) | qHPV  Placebo | HPV-6 = 10% (3/30)  HPV-6 = 3.7% (1/27) | 2.7^ɣ^  (0.3 – 24.4)^##^  Ref: Placebo | 9 |
|  |  |  |  |  |  | qHPV  Placebo | HPV-11 = 0% (0/30)  HPV-11 = 0% (0/27) | -  - | 9 |
|  |  |  |  |  |  | qHPV  Placebo | HPV-16 = 6.7% (2/30)  HPV-16 = 3.7% (1/27) | 1.8^ɣ^ (0.2 – 18.8)^##^  Ref: Placebo | 9 |
|  |  |  |  |  |  | qHPV  Placebo | HPV-18 = 6.7% (2/30)  HPV-18 = 0% (0/27) | -  - | 9 |
|  |  |  |  |  | Anal cytology  (at 48 weeks) | qHPV  Placebo | HSIL = 11.1% (7/63)  HSIL = 11.4% (7/61) | 1.0^ɣ^ (0.4 – 2.6)^##^  Ref: Placebo | 9 |
|  |  |  |  |  | Anal cytology  (at 96 weeks) | qHPV  Placebo | HSIL = 0% (N/A)  HSIL = 2% (1/50) | 0.00 | 9 |
|  |  |  |  |  | Anal cytology  (at 144 weeks) | qHPV  Placebo | HSIL = 1.9% (1/53)  HSIL = 0% (N/A) | -  - | 9 |
|  |  |  |  |  | Anal cytology  (at 192 weeks) | qHPV  Placebo | HSIL = 2% (1/50)  HSIL = 0% (N/A) | -  - | 9 |
|  |  |  |  |  | EAGL/Condyloma  (at 48 weeks) | qHPV  Placebo | 4.6% (3/65)  1.7% (1/63) | 2.9^ɣ^ (0.3 – 27.2)^##^  Ref: Placebo | 9 |
|  |  |  |  |  | EAGL/Condyloma  (at 96 weeks) | qHPV  Placebo | 3.7% (2/54)  5.9% (3/51) | 0.6^ɣ^ (0.1 – 3.6)^##^  Ref: Placebo | 9 |
|  |  |  |  |  | EAGL/Condyloma  (at 144 weeks) | qHPV  Placebo | 3.8% (2/52)  0% (0/N/A) | -  - | 9 |
|  |  |  |  |  | EAGL/Condyloma  (at 192 weeks) | qHPV  Placebo | 0% (0/N/A)  0% (0/N/A) | -  - | 9 |
| B) Single arm trials – with historical controls (N_s_= 2) | | | | | | | | |  |
| McClymont 2019 & McClymont 2020a  (ISRCTN33674451)^16^ | Single arm trial (2008-2017)  Compared to 3 historical control groups  HC1: 2004 -2009  HC2-3: 1993-2002 | Canada | Trial: Women, children & adults, >9 years old (N/A)  HC1: HIV positive women (15–44 years), unvaccinated from Canada | Trial: 279  HC1= 750 | Newly acquired persistent qHPV DNA infection^a^  (unknown) | NA` | Any qHPV = 1.9% (4/212) | - | 9 |
|  |  |  |  |  |  | NRT: qHPV | Any qHPV = 1.9% (5/260) | - | 9 |
|  |  |  |  |  |  | ITT: qHPV | Any qHPV = 4.1% (11/268) | - | 9 |
|  |  |  |  |  |  | qHPV  HC1 | 2.3 (1.1 – 4.1) per 100 PY  6.0 (4.6 – 7.7) per 100 PY | 0.38^o^ (NA)^##^  Ref: HC1 | 9 |
|  |  |  |  |  | Genital warts,  (unknown) | PPE: qHPV | 1.9% (4/211) | - | 7 |
|  |  |  |  |  |  | NRT: qHPV | 3.9% (10/258) | - | 7 |
|  |  |  |  |  |  | ITT: qHPV | 4.2%(11/264) | - | 7 |
|  |  |  |  |  |  | qHPV  HC1 | 2.3 (1.2 -4.1) per 100 PY  2.9 (2.1 – 3.9) per 100 PY | 0.79^o^ (NA)^##^  Ref: HC1 | 7 |
|  |  |  |  |  | CIN 2+  (unknown) | PPE: qHPV | 0.0% (0/177) | - | 7 |
|  |  |  |  |  |  | NRT: qHPV | 0.0% (0/210) | - | 7 |
|  |  |  |  |  |  | ITT: qHPV | 0.0% (0/217) | - | 7 |
|  |  |  |  |  |  | qHPV  HC1 | 0.0 (0.0 – 0.9) per 100 PY  1.0 (0.5 – 1.9) per 100 PY | 0.0 (NA)^##^  Ref: HC1 | 7 |
|  |  | HC2 & HC3: Colombia, France, Germany, Philippines, Spain, Thailand & USA | Trial: Women, adults, 24-45 years (DNA: negative)  HC2: qHPV  HC3: HIV negative women (24-55 years old) ,placebo | HC2 & HC3~1885 | Vaccine failure*^#^*  [occurrence of either persistent qHPV infection, development of CIN2+ or genital wart]  (timing unknown) | PPE: qHPV  HC2  qHPV  HC3 | 1.2 (0.2-3.4) per 100 PY  0.1 (0.02-0.03) per 100 PY  1.2 (0.2-3.4) per 100 PY  1.5 (1.1 -2.0) per 100 PY | 11.7 (2.6 - 52.1)  Ref: HC2  0.8 (0.2 - 2.5)  Ref: HC3 | 7 |
|  |  |  |  |  |  | NRT: qHPV  HC2  qHPV HC3 | 2.0 (0.7-4.5) per 100 PY  0.5 (0.3 -0.9) per 100 PY  2.0 (0.7-4.5) per 100 PY  2.0 (1.6-2.5) per 100 PY | 4.1 (1.6 - 10.2)  Ref: HC2  1.0 (0.4 -2.3)  Ref: HC3 | 7 |
|  |  |  |  |  |  | ITT: qHPV  HC2  qHPV  HC3 | Rate: 3.0 (1.4 -5.7) per 100 PY  Rate: 2.7 (2.2 - 3.3) per 100 PY  Rate: 3.0 (1.4 -5.7) per 100 PY  Rate: 3.9 (3.3 - 4.6) per 100 PY | 1.1 (0.6 - 2.2)  Ref: HC2  0.8 (0.4 -1.5)  Ref: HC3 | 7 |
| Online trial results  (NCT01209325)^13^ | Single arm trial (2011-2017) vs 1 historical control group (HC, 2004-2009) | United States & Puerto Rico  HC: Australia, Brazil, Canada, Costa Rica, Croatia, Finland, Germany, Mexico, Netherlands, Norway, Peru, Philippines, Portugal, South Africa, Spain, Sweden, Taiwan, USA | MSM, adult, 18-27 years old (negative)  HC: HIV negative, boys and men (16-26), placebo | 144 | Incidence of AIN or Anal/Perianal Condyloma (between 28-48 weeks) | qHPV  HC | HPV-6 = 0 per 100 PY  HPV-6 = 11.1 per 100 PY | 0.00  Ref: HC | 7 |
|  |  |  |  |  |  | qHPV  HC | HPV-11 = 0 per 100 PY  HPV-11 = 2.0 per 100 PY | 0.00  Ref: HC | 7 |
|  |  |  |  |  |  | qHPV  HC | HPV-16 = 0 per 100 PY  HPV 16 = 1.8 per 100 PY | 0.00  Ref: HC | 7 |
|  |  |  |  |  |  | qHPV  HC | HPV-18 = 0 per 100 PY  HPV-18 = 2.8 per 100 PY | 0.00  Ref: HC | 7 |
|  |  |  |  |  | Incident persistent anogenital infection at 96 weeks | qHPV | HPV 6 = 1.8 per 100 PY  HPV 11 = 0 per 100 PY  HPV 16 = 2.9 per 100 PY  HPV 18 = 0.7 per 100 PY | - | 7 |
|  |  |  |  |  | Incidence of penile/scrotal warts  (between 28-48 weeks) | qHPV | HPV-6 = 1.8 per 100 PY  HPV-11 = 0.9 per 100 PY  HPV-16 = 1.8 per 100 PY  HPV-18 = 0.7 per 100 PY | - | 7 |
| C) Single arm trials - without historical controls (N_s_= 3) | | | | | | | | |  |
| Cespedes 2018  (NCT00604175)^43^ | Single arm trial: qHPV vaccine (2008-2012) | United States, Brazil & South Africa | Both sex,  Adults & Children, 13-45 years old  (DNA: negative) | 315 | Cervical DNA  (at 28 weeks) | qHPV | HPV 6/11 = 0.0% (0/224)  HPV 16 = 1.8% (4/221)  HPV 18 = 0.4% (1/220) | -  -  - | 10 |
|  |  |  |  |  | Cervical DNA  (at 52 weeks) | qHPV | HPV 6/11 = 0.9% (2/224)  HPV 16 = 2.3% (5/221)  HPV 18 = 0.0% (0/220) | -  -  - | 10 |
|  |  |  |  |  | Anal DNA  (at 28 weeks) | qHPV | HPV 6/11 = 1.8% (5/281)  HPV 16 = 2.4% (5/210)  HPV 18 = 1.4% (3/218) | -  -  - | 10 |
|  |  |  |  |  | Anal DNA  (at 52 weeks) | qHPV | HPV 6/11 = 1.0% (3/281)  HPV 16 = 1.9% (4/210)  HPV 18 = 2.3% (5/218) | -  -  - | 10 |
|  |  |  | Adolescents & Adults, 13-45 years old  (DNA: positive) |  | Cervical DNA  (at 28 weeks) | qHPV | HPV 6/11 = 25.0% (1/4)  HPV 16 = 57.1 % (4/7)  HPV 18 = 75.0 %(6/8) | -  -  - | 10 |
|  |  |  |  |  | Cervical DNA  (at 52 weeks) | qHPV | HPV 6/11 = 25.0 % (1/4)  HPV 16 = 42.9 % (3/7)  HPV 18 = 75.0 %(6/8) | -  -  - | 10 |
|  |  |  |  |  | Anal DNA  (at 28 weeks) | qHPV | HPV 6/11 = 12.3 % (8/65)  HPV 16 = 52.4 % (11/21)  HPV 18 = 61.5 % (8/13) | -  -  - | 10 |
|  |  |  |  |  | Anal DNA  (at 52 weeks) | qHPV | HPV 6/11 = 9.2 % (6/65)  HPV 16 = 52.4 % (11/21)  HPV 18 = 53.8 % (7/13) | -  -  - | 10 |
| Wilkin 2010  (NCT00513526)^22^ | Single arm trial:  qHPV vaccine (2007 – 2011) | United States | Men, Adults, 22-61 years old (DNA: negative) | 112 | Anal DNA  (at 28 weeks) | qHPV | HPV 6 = 5.5% (5/91)  HPV 11 = 3.3% (3/92)  HPV 16 = 2.5% (2/81)  HPV 18 = 1.1% (1/93) | -  -  -  - | 10 |
|  |  |  | (DNA: positive or negative) |  | Anal DNA  (at 28 weeks) | qHPV | HPV 6 = 9.0% (9/100)  HPV 11 = 9.0% (9/100)  HPV 16 = 13.0% (13/100)  HPV 18 = 3.0% (3/100) | -  -  -  - | 10 |
|  |  |  | (DNA: positive) |  | Anal DNA  (at 28 weeks) | qHPV | HPV 6 = 44.4% (4/9)  HPV 11 = 75.0% (6/8)  HPV 16 = 57.9% (11/19)  HPV 18 = 28.6% (2/7) | -  -  -  - | 10 |
|  |  |  | (DNA: negative) |  | Anal cytology  (at 28 weeks) | qHPV | Normal = 47.6% (50/105)  ASCUS = 31.4% (33/105)  LSIL = 15.2% (16/105)  HSIL = 2.9% (3/105)  HGAIN = 11.4% (12/105) | -  -  -  -  - | 8 |
| Palefsky 2021  (NCT00667563)^41^ | Single arm trial:  qHPV vaccine (2008-2016) | India | Women, adults, 27-38 years old (DNA: negative) | 150 | Cervical HPV DNA  (at 28 weeks) | qHPV | HPV-16 = 2% (2/95)  HPV-18 = 1% (1/97) | - | 9 |
|  |  |  |  |  | Cervical HPV DNA  (at 52 weeks) | qHPV | HPV-16 = 0% (0/97)  HPV-18 = 2% (2/99) | - | 9 |

^a^ Newly acquired persistent qHPV infection was defined as the detection of the same qHPV type in samples collected at 2 or more consecutive visits (>6 months apart) or detection of qHPV at the last available visit; ^##^ risk/rate ratios and 95%CIs were derived from available information; ^ɣ^ Risk ratio, ^o^ Rate Ratio; ^itt^ Intention to treat analysis; * test for significance, p-value < 0.05, † p-values were not calculated as no PY were available.

Abbreviations: HPV = Human Papillomavirus, HC = Historical control, qHPV = quadrivalent vaccine; bHPV = bivalent vaccine, NA = Not available, RCT = Randomized controlled trial, CIN = Cervical Intraepithelial Neoplasia, PY = person years; AIN = anal intraepithelial neoplasia; HSIL = high grade squamous intraepithelial lesion; EAGL = external ano-genital lesions; QS: quality score (number of stars) based on tables S9.

**Supplement table S8: Frequency of (serious) adverse events per region and by study.** Abbreviations: (S)AE = (Serious) Adverse Events, bHPV = Bivalent HPV, qHPV = Quadrivalent HPV, 9vHPV = Nonavalent HPV, N_s_= Number of studies, SOT = solid organ transplant.

| **A) Summary of safety results available from the 18 trials included in the review – vaccine arm during the trial** | | | | | | | | | | |
| --- | --- | --- | --- | --- | --- | --- | --- | --- | --- | --- |
| **Region** | **HIV status** | **Vaccine** | **Number of trials with safety results**  **(n_s_)** | **Frequency of Adverse events* (AEs)**  **(possibly, probably or definitely related to vaccine)**  **Frequency** | | | **Frequency of other AEs****  **(not serious)**  **Frequency** | | **Frequency of Serious Adverse events*** (SAEs)**  **Frequency** | |
| Americas | Positive | qHPV | 6 | <0.5%  0.5%-45%  46-50%  51%-100% | | N_s_= 4  N_s_=0  N_s_=1  N_s_= 0 | <0.5%  0.5%-45%  46-50%  51%-100% | N_s_= 0  N_s_=3  N_s_=2  N_s_= 1 | <0.5%  0.5%-15%  16-50%  51%-100% | N_s_= 2  N_s_=4  N_s_=0  N_s_= 0 |
| Europe | Positive | bHPV, qHPV or 9vHPV | 3 | <0.5%  0.5%-45%  46-50%  51%-100% | | N_s_= 0  N_s_=0  N_s_=0  N_s_= 0 | <0.5%  0.5%-45%  46-50%  51%-100% | N_s_= 0  N_s_=0  N_s_=0  N_s_= 3 | <0.5%  0.5%-15%  16-50%  51%-100% | N_s_= 3  N_s_=0  N_s_=0  N_s_= 0 |
| Africa | Positive | bHPV or qHPV | 2 | <0.5%  0.5%-45%  46-50%  51%-100% | | N_s_= 0  N_s_=0  N_s_=0  N_s_= 0 | <0.5%  0.5%-45%  46-50%  51%-100% | N_s_= 0  N_s_=0  N_s_=0  N_s_= 2 | <0.5%  0.5%-15%  16-50%  51%-100% | N_s_= 0  N_s_=2  N_s_=0  N_s_= 0 |
| Elsewhere | Positive | bHPV or qHPV | 3 | <0.5%  0.5%-45%  46-50%  51%-100% | | N_s_= 0  N_s_=0  N_s_=0  N_s_= 0 | <0.5%  0.5%-45%  46-50%  51%-100% | N_s_= 0  N_s_=0  N_s_=0  N_s_= 3 | <0.5%  0.5%-15%  16-50%  51%-100% | N_s_= 0  N_s_=3  N_s_=0  N_s_= 0 |
| Total | Positive | bHPV | 3 | <0.5%  0.5%-45%  46-50%  51%-100% | | N_s_= 0  N_s_=0  N_s_=0  N_s_= 0 | <0.5%  0.5%-45%  46-50%  51%-100% | N_s_= 0  N_s_=0  N_s_=0  N_s_= 3 | <0.5%  0.5%-15%  16-50%  51%-100% | N_s_= 1  N_s_=2  N_s_=0  N_s_= 0 |
|  |  | qHPV | 12 | <0.5%  0.5%-45%  46-50%  51%-100% | | N_s_= 4  N_s_=0  N_s_=1  N_s_= 0 | <0.5%  0.5%-45%  46-50%  51%-100% | N_s_= 0  N_s_=3  N_s_=1  N_s_= 8 | <0.5%  0.5%-15%  16-50%  51%-100% | N_s_= 4  N_s_=8  N_s_=0  N_s_= 0 |
|  |  | 9vHPV | 1 | <0.5%  0.5%-45%  46-50%  51%-100% | | N_s_= 0  N_s_=0  N_s_=0  N_s_= 0 | <0.5%  0.5%-45%  46-50%  51%-100% | N_s_= 0  N_s_=0  N_s_=0  N_s_= 1 | <0.5%  0.5%-15%  16-50%  51%-100% | N_s_= 0  N_s_=1  N_s_=0  N_s_= 0 |
|  |  | Any | 14 | <0.5%  0.5%-45%  46-50%  51%-100% | | N_s_= 4  N_s_=0  N_s_=1  N_s_= 0 | <0.5%  0.5%-45%  46-50%  51%-100% | N_s_= 0  N_s_=3  N_s_=1  N_s_= 10 | <0.5%  0.5%-15%  16-50%  51%-100% | N_s_= 4  N_s_=10  N_s_=0  N_s_= 0 |
| **B) Detailed safety results from each study** | | | | | | | | | | |
| **Trial**  **(Location)** | **HIV status** | **Vaccine arm** | **Timing** | **Adverse events (AEs)**  **(possibly, probably or definitely related to vaccine)** | | | **Other AEs**  **(not serious)** | | **Serious Adverse events (SAEs)** | |
|  |  |  |  | **Outcome** | **Estimate** | | **Outcome** | **Estimate** | **Outcome** | **Estimate** |
| **Region: Americas** |  |  |  |  |  | |  |  |  |  |
| 1) NCT00339040^7^ (NIAID)/ IMPAACT P1047  United States & Puerto Rico | Positive | qHPV | Within 14 days after 1st dose | Grade*** 3 or 4 AEs | 0%  (0/96) | | Grade 3 or 4 AE | 7.3% (7/96) | NA | NA |
|  |  |  | 1st dose - study completion | NA | NA | | Adverse events | 96.9% (93/96) | Serious adverse events | 5.2% (5/96) |
|  |  | Placebo | Within 14 days after 1st dose | Grade 3 or 4 AEs | 0%  (0/30) | | Grade 3 or 4 AE | 6.7%  (2/30) | NA | NA |
|  |  |  | 1st dose - study completion | NA | NA | | Adverse events | 100% (30/30) | Serious adverse events | 6.7% (2/30) |
| 2) NCT01206556^9^ / IMPAACT P1085 (follow up: IMPAACT P1047)  United States & Puerto Rico | NA | NA | NA | NA | NA | | NA | NA | NA | NA |
| 3) NCT01461096^11^ (NIAID)  United States, Brazil & Puerto Rico | Positive | qHPV | From baseline - to last visit (up to 4 years) | Grade 3 or 4 AEs possibly, probably or definitely related to the vaccine | 0.3% (1/288) | | Signs/symptoms >=Grade 3, laboratory results >=Grade 3 and events that led to a change in treatment, regardless of grade | 30.2% (87/288) | Signs/symptoms >=Grade 3, laboratory results >=Grade 3 and events that led to a change in treatment, regardless of grade | 11.5% (33/288) |
|  |  | Placebo | From baseline - to last visit (up to 4 years) | Grade 3 or 4 AEs possibly, probably or definitely related to the vaccine | 0.0% (0/286) | | Signs/symptoms >=Grade 3, laboratory results >=Grade 3 and events that led to a change in treatment, regardless of grade | 33.1% (95/287) | Signs/symptoms >=Grade 3, laboratory results >=Grade 3 and events that led to a change in treatment, regardless of grade | 16.0% (46/287) |
| 4) NCT02236234^12^  (University of Sao Paulo) Brazil | Positive | bHPV | NA | NA | NA | | NA | NA | NA | NA |
| 5) NCT01209325^13^ (AIDS Malignancy Consortium) USA and Puerto Rico | Positive | qHPV | Enrollment - 2 years after first injection | Occurrence of Grade ≥ 3 Adverse Events (AEs) possibly, probably, or definitely related to the vaccine | 0% (0/144) | | All Grade ≥ 2 laboratory values (except for CD4 cell counts) were recorded as AEs. After Week 32, only SAEs and AEs or symptoms > grade 3 or symptoms that were possibly, probably, or definitely related to the study vaccine were recorded. | 41.0% (49/144) | All Grade ≥ 2 laboratory values (except for CD4 cell counts) were recorded as AEs. After Week 32, only SAEs and AEs or symptoms > grade 3 or symptoms that were possibly, probably, or definitely related to the study vaccine were recorded. | 3.5% (5/144) |
| 6) ISRCTN33674451^18,19^  Canada | Positive | qHPV | Up to 30 days after vaccination | NA | NA | | Adverse events | 35.8% (111/310) | Serious adverse events possibly associated with vaccination | 0.3% (1/310) |
|  |  |  | After Dose 1 | NA | NA | | Adverse events | 26.5% (82/310) | NA | NA |
|  |  |  | After Dose 2 | NA | NA | | Adverse events | 19.2% (56/291) | NA | NA |
|  |  |  | After Dose 3 | NA | NA | | Adverse events | 22.0% (61/277) | NA | NA |
| 7) NCT00513526^23^ (AIDS Malignancy Consortium) United States | Positive | qHPV | NA | At least Grade 3 AEs probably or definitely related to the vaccine | 0% (0/109) | | Adverse events (at least grade 2) | 51.4% (56/109) | Serious adverse events | 4.6% (5/109) |
| 8) NCT00710593^26^  (University of North Carolina) United States & Puerto Rico | Positive | qHPV: All | Enrollment - Week 24 | At Least One AEs possibly, Probably, or Definitely Related to Vaccine | 48.5% (48/99) | | NA | NA | Serious adverse events related to the vaccine | 0% (0/99) |
|  |  | qHPV: ART Naïve or no ART for 6 months before study entry | Enrollment - Week 24 | At Least One AEs possibly, Probably, or Definitely Related to Vaccine | 49.3% (34/69) | | NA | NA | Serious adverse events related to the vaccine | 0% (0/69) |
|  |  | qHPV: On ART for at least 6 months | Enrollment - Week 24 | At Least One AEs possibly, Probably, or Definitely Related to Vaccine | 46.7% (14/30) | | NA | NA | Serious adverse events related to the vaccine | 0% (0/30) |
|  |  | qHPV | After dose 1 | NA | NA | | Adverse events (grade<= 3) | 24.2% (24/99) | NA | NA |
|  |  |  | After dose 2 | NA | NA | | Adverse events (grade<= 3) | 26.3% (26/99) | NA | NA |
|  |  |  | After dose 3 | NA | NA | | Adverse events (grade<= 3) | 48.5% (48/99) | NA | NA |
| **Region: Europe** |  |  |  |  |  | |  |  |  |  |
| 9) NCT01386164^46^ (University of Aarhus)  Denmark | Positive | bHPV or qHPV | Enrollment - 15 days post vaccination | NA | NA | | NA | NA | Serious adverse events | 0% (0/91) |
|  |  | bHPV | Enrollment - 15 days post vaccination | NA | NA | | NA | NA | Serious adverse events | 0% (0/45) |
|  |  |  | During the first 4 days after dose 1 | NA | NA | | Adverse events | 84.4% (38/45) | NA | NA |
|  |  |  | During the first 4 days after dose 2 | NA | NA | | Adverse events | 52.3%  (23/45) | NA | NA |
|  |  |  | During the first 4 days after dose 3 | NA | NA | | Adverse events | 61.4% (27/44) | NA | NA |
|  |  | qHPV | Enrollment - 15 days post vaccination | NA | NA | | NA | NA | Serious adverse events | 0% (0/46) |
|  |  |  | During the first 4 days after dose 1 | NA | NA | | Adverse events | 54.3% (25/46) | NA | NA |
|  |  |  | During the first 4 days after dose 2 | NA | NA | | Adverse events | 28.2% (13/46) | NA | NA |
|  |  |  | During the first 4 days after dose 3 | NA | NA | | Adverse events | 39.1% (18/46) | NA | NA |
| 10) ISRCTN14732216^32^  Spain | Positive | qHPV | After Dose 1 | NA | NA | | Adverse events (1-4) | 54.4% (36/66) | NA | NA |
|  |  |  | After Dose 2 | NA | NA | | Adverse events (1-4) | 89.4% (59/66) | NA | NA |
|  |  |  | After Dose 3 | NA | NA | | Adverse events (1-4) | 66.7% (44/66) | NA | NA |
|  |  |  | After dose 1/2 or 3 | NA | NA | | NA | NA | Serious adverse events | 0% (0/66) |
|  |  | Placebo | After Dose 1 | NA | NA | | Adverse events (1-4) | 87.3% (55/63) | NA | NA |
|  |  |  | After Dose 2 | NA | NA | | Adverse events (1-4) | 98.4% (62/63) | NA | NA |
|  |  |  | After Dose 3 | NA | NA | | Adverse events (1-4) | 91.9%  (57/62) | NA | NA |
|  |  |  | After dose 1/2 or 3 | NA | NA | | NA | NA | Serious adverse events | 0% (0/63) |
| 11) NCT01512784^33^  Italy | NA | NA | NA | NA | NA | | NA | NA | NA | NA |
| 12) NCT03525210^34^ (Universitaire Ziekenhuizen Leuven) Belgium | Positive | 9vHPV | 1 month post 3rd dose | NA | NA | | Adverse events | 100% (99/99) | Serious adverse events | 3.0%  (3/99) |
|  | Negative (SOT patients) | 9vHPV | 1 month post 3rd dose | NA | NA | | Adverse events | 100% (170/170) | Serious adverse events | 16.5% (28/170) |
| 13) NA^36^  Poland | NA | NA | NA | NA | NA | | NA | NA | NA | NA |
| **Region: Africa** |  |  |  |  |  | |  |  |  |  |
| 14) NCT00586339^4^ (GlaxoSmithKline) South Africa | Positive | bHPV | Enrollment - 7 months | NA | NA | | Adverse events | 100% (61/61) | Serious adverse events | 4.9% (3/61) |
|  |  | Placebo | Enrollment - 7 months | NA | NA | | Adverse events | 93.2% (55/59) | Serious adverse events | 3.4%  (2/59) |
|  | Negative | bHPV | Enrollment - 7 months | NA | NA | | Adverse events | 96.7% (29/30) | Serious adverse events | 3.3% (1/30) |
| 15) NCT00557245^39^ (University of Washington) Kenya & Uganda | Positive | qHPV | Up to 12 months | NA | NA | | Adverse events | 74.4% (134/180) | Serious adverse events | 2.8% (5/180) |
| **Region: Others** |  |  |  |  |  | |  |  |  |  |
| 16) NCT00604175/ A5240 (NIAID)^45^  United States, Brazil & South Africa | Positive | qHPV - All | From baseline up to week 72 | NA | NA | | Signs and symptoms and laboratory abnormalities of >=Grade 2 and all grades of fever. | 70.0%  (249/315) | Signs and symptoms and laboratory abnormalities of >=Grade 2 and all grades of fever. | 4.4% (14/315) |
|  |  | qHPV - A: CD4 > 350 | From baseline up to week 72 | NA | NA | | Signs and symptoms and laboratory abnormalities of >=Grade 2 and all grades of fever. | 75.6%  (96/127) | Signs and symptoms and laboratory abnormalities of >=Grade 2 and all grades of fever. | 4.7% (6/127) |
|  |  | qHPV –  B: CD4 >200 <350 | From baseline up to week 72 | NA | NA | | Signs and symptoms and laboratory abnormalities of >=Grade 2 and all grades of fever. | 74.7%  (71/95) | Signs and symptoms and laboratory abnormalities of >=Grade 2 and all grades of fever. | 2.1% (2/95) |
|  |  | qHPV –  C: CD4 <200 | From baseline up to week 72 | NA | NA | | Signs and symptoms and laboratory abnormalities of >=Grade 2 and all grades of fever. | 88.2%  (82/93) | Signs and symptoms and laboratory abnormalities of >=Grade 2 and all grades of fever. | 6.5% (6/93) |
| 17) NCT00667563^42^ India | Positive | qHPV | Enrollment – week 52 | Number of grade 3 or 4 adverse events attributed to vaccine per 100 patients | 6.0 (95%CI: 3.1 – 11.6) | | Adverse events | 96.7% (145/150) | Serious Adverse Events | 2.7% (4/150) |
| 18) NCT01031069^40^ (GSK)  Brazil, Estonia, India, & Thailand | Positive | bHPV or qHPV | Enrollment - 7 months | NA | NA | | Adverse events | 90.3%  (232/257) | Serious adverse events | 7.0%  (18/257) |
|  |  | bHPV | Enrollment - 7 months | NA | NA | | Adverse events | 95.4% (123/129) | Serious adverse events | 7.0% (9/129) |
|  |  | qHPV | Enrollment - 7 months | NA | NA | | Adverse events | 85.2%  (109/128) | Serious adverse events | 7.0% (9/128) |
|  | Negative | bHPV | Enrollment - 7 months | NA | NA | | Adverse events | 94.4% (136/144) | Serious adverse events | 2.8% (4/144) |
|  |  | qHPV | Enrollment - 7 months | NA | NA | | Adverse events | 87.6% (127/145) | Serious adverse events | 0.7%  (1/145) |

* Participants with at least one adverse event that was possibly, probably or definitely related to the vaccine.

** Adverse events – “Any untoward or unfavorable medical occurrence in a participant, including any abnormal sign (for example, abnormal physical exam or laboratory finding), symptom, or disease, temporally associated with the participant’s participation in the research, whether or not considered related to the participant’s participation in the research.”^47^

*** Serious adverse events – “ Include adverse events that result in any of the following outcomes: death, a life-threatening adverse event, inpatient hospitalization or prolongation of existing hospitalization, a persistent or significant incapacity or substantial disruption of the ability to conduct normal functions, or a congenital anomaly/birth defect. Important medical events that may not result in death, be life-threatening, or require hospitalization may be considered serious when, based upon appropriate medical judgment, they may jeopardize the participant and may require medical or surgical intervention to prevent one of the outcomes listed in this definition.” ^47^

**Supplement table S9: Quality assessment of available estimates from each publication reporting immunogenicity results on A) seropositivity and B) antibody titers from a study.** Evaluation performed at the publication level using the 12 criteria in supplement table S2A since not all publication report the same results from a given study

1. **Immunogenicity (seropositivity) (N_p_=30)**

| **Reference** | **Study design** | | **Representativeness** | | | **Misclassification bias** | | | **Measurement error** | | | | **Total**  **Score** | **Figures where seropositivity results included** |
| --- | --- | --- | --- | --- | --- | --- | --- | --- | --- | --- | --- | --- | --- | --- |
|  | **1)**  **Aim** | **2)**  **Population** | **3)**  **Participants** | **4)**  **Recruitment** | **5)**  **Enrollment rate and loss-to-follow up** | **6)**  **Intervention exposure** | **7)**  **Assessment of outcome**  **at baseline** | **8)**  **Analysis by previous exposure** | **9)**  **Sample size** | **10)**  **Methods** | **11)**  **Specificity** | **12)**  **Reporting** | **Maximum of 12** |  |
| **America** | | | | | | | | | | | | | | |
| **Trial: NCT00339040 (NIAID)/ IMPAACT P1047** | | | | | | | | | | | | | |  |
| Weinberg 2012 | ***** | ***** | ***** | **-** | ***** | **-** | ***** | ***** | ***** | ***** | ***** | ***** | **10** | **2a-d** |
| Levin 2010 | ***** | ***** | ***** | **-** | ***** | **-** | ***** | ***** | ***** | ***** | ***** | ***** | **10** | **No^%^** |
| Trial data online | ***** | ***** | ***** | **-** | ***** | **-** | ***** | ***** | ***** | ***** | ***** | ***** | **10** | **No^%^** |
| **Trial: NCT01206556 / IMPAACT P1085 (follow up: IMPAACT P1047)** | | | | | | | | | | | | | |  |
| Levin 2017 | ***** | ***** | ***** | **-** | ***** | **-** | ***** | ***** | **-** | ***** | ***** | ***** | **9** | **2a-d** |
| **Trial: ISRCTN33674451** | | | | | | | | | | | | | |  |
| Brophy 2018 | ***** | ***** | ***** | ***** | ***** | ***** | ***** | ***** | **-** | ***** | ***** | ***** | **11** | **No**^γ^ |
| Money 2016 | ***** | ***** | ***** | ***** | ***** | ***** | ***** | ***** | **-** | ***** | ***** | ***** | **11** | **2a-d** |
| Trial data online | ***** | ***** | ***** | ***** | ***** | ***** | ***** | ***** | **-** | ***** | ***** | ***** | **11** | **No**^γ^ |
| **Trial: NCT00513526 (AIDS Malignancy Consortium)** | | | | | | | | | | | | | |  |
| Pinto 2019 | ***** | ***** | ***** | ***** | ***** | ***** | ***** | **-** | **-** | ***** | ***** | ***** | **10** | **S3a-d** |
| Ellsworth 2018 | ***** | ***** | ***** | ***** | ***** | ***** | ***** | **-** | **-** | ***** | ***** | ***** | **10** | **S3a-d** |
| Wilkin 2010 | ***** | ***** | ***** | ***** | ***** | ***** | ***** | ***** | ***** | ***** | ***** | **-** | **11** | **2a-d** |
| Trial data online | ***** | ***** | ***** | ***** | ***** | ***** | ***** | ***** | ***** | ***** | ***** | **-** | **11** | **No**^γ^ |
| **Trial: NCT00710593 (University of North Carolina)** | | | | | | | | | | | | | |  |
| Kahn 2013 | **-** | ***** | ***** | ***** | ***** | ***** | ***** | ***** | **-** | ***** | ***** | ***** | **10** | **2a-d** |
| Kahn 2017 | **-** | ***** | ***** | ***** | ***** | ***** | ***** | ***** | **-** | ***** | ***** | ***** | **10** | **2a-d** |
| Trial data online | **-** | ***** | ***** | ***** | ***** | ***** | ***** | ***** | **-** | ***** | ***** | ***** | **10** | **No**^γ^ |
| **Europe** | | | | | | | | | | | | | | |
| **Trial: NCT01386164 (University of Aarhus)** | | | | | | | | | | | | | |  |
| Faust 2016 | ***** | ***** | ***** | **-** | **-** | ***** | ***** | ***** | **-** | ***** | ***** | ***** | **9** | **2a-d** |
| **Trial: NCT01512784** | | | | | | | | | | | | | |  |
| Giacomet 2014 | ***** | ***** | ***** | **-** | ***** | ***** | ***** | ***** | **-** | ***** | **-** | **-** | **8** | **No^#^** |
| **Trial: NCT03525210** | | | | | | | | | | | | | |  |
| Boey 2020 | ***** | ***** | ***** | **-** | ***** | ***** | ***** | ***** | ***** | ***** | ***** | ***** | **11** | **2a-d** |
| Trial data online | ***** | ***** | ***** | **-** | ***** | ***** | ***** | ***** | ***** | ***** | ***** | ***** | **11** | **No**^γ^ |
| **Trial: N/A** | | | | | | | | | | | | | |  |
| Oldakowska 2012 | ***** | ***** | **-** | **-** | ***** | **-** | **-** | **-** | **-** | ***** | ***** | **-** | **5** | **S1a-d** |
| **Africa** | | | | | | | | | | | | | | |
| **NCT00586339 (GlaxoSmithKline)** | | | | | | | | | | | | | |  |
| Denny 2013 | ***** | ***** | ***** | ***** | **-** | ***** | **-** | **-** | ***** | ***** | ***** | ***** | **9** | **S1a-d** |
| Trial data online | ***** | ***** | ***** | ***** | **-** | ***** | **-** | **-** | ***** | ***** | ***** | ***** | **9** | **No**^γ^ |
| **Trial: NCT00557245 (University of Washington)** | | | | | | | | | | | | | |  |
| Mugo 2021 | ***** | ***** | ***** | **-** | ***** | **-** | ***** | ***** | ***** | ***** | ***** | ***** | **10** | **2a-d** |
| Mugo 2018 | ***** | ***** | ***** | **-** | ***** | **-** | ***** | ***** | ***** | ***** | ***** | ***** | **10** | **2a-d** |
| **Other and/or mixed regions** | | | | | | | | | | | | | | |
| **Trial: NCT01031069** | | | | | | | | | | | | | |  |
| Folschweiller 2020 | ***** | ***** | ***** | **-** | ***** | ***** | ***** | ***** | ***** | ***** | ***** | **-** | **10** | **2a-b** |
| Trial data online | ***** | ***** | ***** | **-** | ***** | ***** | ***** | ***** | ***** | ***** | ***** | **-** | **10** | **No**^γ^ |
| **Trial: NCT00667563** | | | | | | | | | | | | | |  |
| Palefsky 2021 | ***** | ***** | ***** | ***** | ***** | **-** | ***** | ***** | ***** | ***** | ***** | **-** | **10** | **2a-b** |
| Trial data online | ***** | ***** | ***** | ***** | ***** | **-** | ***** | ***** | ***** | ***** | ***** | **-** | **10** | **No**^γ^ |
| **Trial: NCT00604175/ A5240 (NIAID)** | | | | | | | | | | | | | |  |
| Cespedes 2018 | ***** | ***** | ***** | ***** | ***** | ***** | ***** | ***** | ***** | ***** | ***** | ***** | **12** | **2a-d** |
| Kojic 2014 | ***** | ***** | ***** | ***** | ***** | ***** | ***** | ***** | ***** | ***** | ***** | **-** | **11** | **2a-d** |
| Trial data online | ***** | ***** | ***** | ***** | ***** | ***** | ***** | ***** | ***** | ***** | ***** | ***** | **12** | **No**^γ^ |
| **Range of quality score for studies included in the main results among participants seronegative at baseline (Figure 1a-d) (N_s_=11)** | | | | | | | | | | | | | | **9-12** |
| **Range of quality score for studies included in the additional results among participants with mixed or unknown serostatus at baseline (Supplement figure 2a-d) (N_s_=4)** | | | | | | | | | | | | | | **5-10** |

# = not included as the seropositivity results were not stratified by HPV type, γ = duplicate: other results from the same study used in the main analysis

**B)** Publications reporting data on Immunogenicity (antibody titers) (N_p_=23)

| **Reference** | **Study design** | | **Representativeness** | | | **Misclassification bias** | | | | **Measurement error** | | | | **Total**  **Score** | **Figures where GMT results included** |
| --- | --- | --- | --- | --- | --- | --- | --- | --- | --- | --- | --- | --- | --- | --- | --- |
|  | **1)**  **Aim** | **2)**  **Population** | **3)**  **Participants** | **4)**  **Recruitment** | **5)**  **Enrollment rate and loss-to-follow up** | **6)**  **Intervention exposure** | | **7)**  **Assessment of outcome**  **at baseline** | **8)**  **Analysis by previous exposure** | **9)**  **Sample size** | **10)**  **Methods** | **11)**  **Specificity** | **12)**  **Reporting** | **Maximum of 12** |  |
| **America** | | | | | | | | | | | | | | |  |
| **Trial: NCT00339040 (NIAID)/ IMPAACT P1047** | | | | | | | | | | | | | | |  |
| Levin 2010 | ***** | ***** | ***** | **-** | ***** | | **-** | ***** | ***** | ***** | ***** | ***** | ***** | **10** | **2 S2, S6** |
| Trial data online | ***** | ***** | ***** | **-** | ***** | | **-** | ***** | ***** | ***** | ***** | ***** | ***** | **10** | **No**^γ^ |
| **Trial: NCT01206556 / IMPAACT P1085 (follow up: IMPAACT P1047)** | | | | | | | | | | | | | | |  |
| Weinberg 2018 | ***** | ***** | ***** | ***** | ***** | | **-** | ***** | ***** | **-** | ***** | ***** | ***** | **10** | **S2** |
| Levin 2017 | ***** | ***** | ***** | ***** | ***** | | **-** | ***** | ***** | **-** | ***** | ***** | ***** | **10** | **S7, S2** |
| **Trial: NCT01209325** | | | | | | | | | | | | | | |  |
| Online,  PI: Palefsky | **-** | ***** | **-** | **-** | ***** | | ***** | ***** | ***** | **-** | **-** | ***** | ***** | **7** | **S7, S2** |
| **Trial: ISRCTN33674451** | | | | | | | | | | | | | | |  |
| McClymont 2020b | ***** | ***** | ***** | ***** | ***** | | ***** | ***** | ***** | ***** | ***** | ***** | **-** | **11** | **No**^γ^ |
| Brophy 2018 | ***** | ***** | ***** | ***** | ***** | | ***** | ***** | ***** | **-** | ***** | ***** | ***** | **11** | **S7, S2, S6** |
| **Trial: NCT00513526 (AIDS Malignancy Consortium)** | | | | | | | | | | | | | | |  |
| Pinto 2019 | ***** | ***** | ***** | ***** | ***** | | ***** | ***** | ***** | **-** | ***** | ***** | ***** | **11** | **S7, S2** |
| Ellsworth 2018 | ***** | ***** | ***** | ***** | ***** | | ***** | ***** | **-** | **-** | ***** | ***** | ***** | **10** | **2** |
| Wilkin 2010 | ***** | ***** | ***** | ***** | ***** | | ***** | ***** | ***** | ***** | ***** | ***** | **-** | **11** | **2, S2** |
| **Trial: NCT00710593 (University of North Carolina)** | | | | | | | | | | | | | | |  |
| Kahn 2013 | ***** | ***** | ***** | ***** | ***** | | ***** | ***** | ***** | **-** | ***** | ***** | ***** | **11** | **2,S7, S2** |
| Kahn 2017 | ***** | ***** | ***** | ***** | ***** | | ***** | ***** | ***** | **-** | ***** | ***** | ***** | **11** | **2, S2, S7** |
| Trial data online | ***** | ***** | ***** | ***** | ***** | | ***** | ***** | ***** | **-** | ***** | ***** | ***** | **11** | **No**^$^ |
| **Europe** | | | | | | | | | | | | | | |  |
| **Trial: NCT01386164 (University of Aarhus)** | | | | | | | | | | | | | | |  |
| Toft 2014a | ***** | ***** | ***** | **-** | **-** | | ***** | ***** | ***** | **-** | ***** | ***** | **-** | **8** | **2, S2, S3,**  **S4** |
| Toft 2014b | ***** | ***** | ***** | **-** | **-** | | ***** | ***** | ***** | **-** | ***** | ***** | ***** | **9** | **No^#^** |
| **Trial: NCT03525210** | | | | | | | | | | | | | | |  |
| Boey 2020 | ***** | ***** | ***** | **-** | ***** | | ***** | ***** | ***** | **-** | ***** | ***** | ***** | **10** | **S7, S2** |
| **Africa** | | | | | | | | | | | | | | |  |
| **NCT00586339 (GlaxoSmithKline)** | | | | | | | | | | | | | | |  |
| Denny 2013 | ***** | ***** | ***** | ***** | **-** | | ***** | **-** | **-** | ***** | ***** | ***** | ***** | **9** | **S7, S2, S3** |
| **Trial: NCT00557245 (University of Washington)** | | | | | | | | | | | | | | |  |
| Mugo 2021 | ***** | ***** | ***** | **-** | ***** | | **-** | ***** | ***** | ***** | ***** | ***** | ***** | **10** | **2** |
| Mugo 2018 | ***** | ***** | ***** | **-** | ***** | | **-** | ***** | ***** | ***** | ***** | ***** | ***** | **10** | **2, S7, S2** |
| **Other and/or mixed regions** | | | | | | | | | | | | | | |  |
| **Trial: NCT01031069** | | | | | | | | | | | | | | |  |
| Folschweiller 2020 | ***** | ***** | ***** | ***** | ***** | | ***** | ***** | ***** | ***** | ***** | ***** | **-** | **11** | **2, S7, S2,**  **S2** |
| **Trial: NCT00604175/ A5240 (NIAID)** | | | | | | | | | | | | | | |  |
| Cespedes 2018 | **-** | ***** | ***** | ***** | ***** | | ***** | ***** | ***** | ***** | ***** | ***** | ***** | **11** | **2, S2, S7** |
| Kojic 2014 | **-** | ***** | ***** | ***** | ***** | | ***** | ***** | ***** | ***** | ***** | ***** | **-** | **10** | **2, S2** |
| Trial data online | **-** | ***** | ***** | ***** | ***** | | ***** | ***** | ***** | ***** | ***** | ***** | ***** | **11** | **No**^γ^ |
| **Range of quality score for studies included in Figure 2 (N_s_=10)** | | | | | | | | | | | | | | | **7-11** |
| **Range of quality score for studies included in Figure 3 (N_s_=7)** | | | | | | | | | | | | | | | **9-11** |
| **Range of quality score for studies included in Figure S3 (N_s_=12)** | | | | | | | | | | | | | | | **7-11** |
| **Range of quality score for studies included in Figure S4 (N_s_=3)** | | | | | | | | | | | | | | | **8-11** |
| **Range of quality score for studies included in Figure S4 (N_s_=1)** | | | | | | | | | | | | | | | **8** |
| **Range of quality score for studies included in Figure S7 (N_s_=4)** | | | | | | | | | | | | | | | **10-11** |

^#^ Study only included results for HPV types not included in the vaccine, ^$^ only results on ‘peak’ antibody titer but no timing available, ^%^ Duplicate: other results from the same study used in the main analysis.

**Supplement table S10: Quality assessment of available estimates from publications (N_p_=10) reporting on clinical endpoints (e.g. Anogenital HPV DNA or cytology)** Evaluation performed at the publication level using the 15 criteria in supplement table S2B since not all publication report the same results from a given study

|  | **Study design** | | | **Representativeness** | | | | | | **Misclassification bias** | | | | | | **Measurement error** | | | | | | | **Internal validity** | | | |  | **Total**  **Score** |
| --- | --- | --- | --- | --- | --- | --- | --- | --- | --- | --- | --- | --- | --- | --- | --- | --- | --- | --- | --- | --- | --- | --- | --- | --- | --- | --- | --- | --- |
| **Reference & outcome assessed** | **1)**  **Aim** | | **2)**  **Population** | **3)**  **Partici-pants** | | **4)**  **Recruit-ment** | | **5)**  **Enrollment rate and loss-to-follow up** | | **6)**  **Intervention exposure** | | **7)**  **Outcome at baseline** | | **8)**  **Analysis by previous exposure** | | **9)**  **Sample size** | | **10)**  **Methods** | | **11)**  **Specificity** | **12)**  **Follow-up** | | **13)**  **Comparator** | **14)**  **Comparability** | | **15)**  **Confounding** | **16) Reporting** | **Maximum of 16** |
| **A) Randomized controlled trials (N_s_= 3)** | | | | | | | | | | | | | | | | | | | | | | | | | | | | |
| **Trial:** NCT01386164 | | | | | | | | | | | | | | | | | | | | | | | | | | | | |
| Toft 2014a:  Anogenital HPV DNA | **-*** | | ***** | ***** | | ***** | | **-** | | | ***** | | ***** | | **-** | | **-** | | ***** | ***** | | ***** | **-** | ***** | | ***** | ***** | **11** |
| Toft 2014b: Anogenital HPV DNA | **-*** | | ***** | ***** | | ***** | | **-** | | | ***** | | ***** | | ***** | | **-** | | ***** | **-** | | ***** | **-** | ***** | | ***** | ***** | **11** |
| **Trial: NCT01461096** | | | | | | | | | | | | | | | | | | | | | | | | | | | | |
| Wilkin 2018 &  Trial data online  Anal HPV DNA | ****** | | ***** | ***** | | **-** | | **-** | | | ***** | | ***** | | ***** | | ***** | | ***** | ***** | | ***** | ***** | | ***** | ***** | ***** | **14** |
| Oral HPV DNA | ***-** | | ***** | ***** | | **-** | | **-** | | | ***** | | ***** | | ***** | | ***** | | ***** | ***** | | ***** | ***** | | ***** | ***** | ***** | **14** |
| Anal cytology | ****** | | ***** | ***** | | **-** | | **-** | | | ***** | | ***** | | **-** | | **-** | | **-** | **-** | | ***** | ***** | | ***** | ***** | ***** | **9** |
| **Trial: ISRCTN14732216** | | | | | | | | | | | | | | | | | | | | | | | | | | | | |
| Hidalgo-Tenorio 2021:  Anal HPV DNA | ****** | | ***** | ***** | | **-** | | **-** | | | ***** | | ***** | | ***** | | **-** | | ***** | **-** | | ***** | ***** | | ***** | ***** | ***** | **12** |
| HSIL/EAGL/Condyloma | ****** | | **-** | ***** | | **-** | | **-** | | | **-** | | **-** | | **-** | | **-** | | ***** | **-** | | ***** | ***** | | ***** | ***** | ***** | **8** |
| **B) Single arm trials – with historical controls (N_s_= 2)** | | | | | | | | | | | | | | | | | | | | | | | | | | | | |
| **Trial: ISRCTN33674451** | | | | | | | | | | | | | | | | | | | | | | | | | | | | |
| McClymont 2019:  HPV DNA infection | ***-** | | ***** | ***** | | ***** | | **-** | | | ***** | | ***** | | ***** | | **-** | | ***** | **-** | | ***** | **-** | | **-** | ***** | **-** | **10** |
| Genital warts | ***-** | | ***** | ***** | | ***** | | **-** | | | ***** | | ***** | | **-** | | **-** | | **-** | **-** | | ***** | **-** | | **-** | ***** | **-** | **8** |
| CIN 2+ | ***-** | | ***** | ***** | | ***** | | **-** | | | ***** | | ***** | | **-** | | **-** | | **-** | **-** | | ***** | **-** | | **-** | ***** | **-** | **8** |
| McClymont 2020a:  HPV DNA infection | ***-** | | ***** | ***** | | ***** | | **-** | | | ***** | | ***** | | ***** | | **-** | | **-** | ***** | | ***** | **-** | | **-** | **-** | **-** | **9** |
| **Trial: NCT01209325** | | | | | | | | | | | | | | | | | | | | | | | | | | | | |
| Online, PI - Palefsky:  Genital warts | **-*** | ***** | | | **-** | | ***** | | **-** | | ***** | | ***** | | **-** | | **-** | | ***** | **-** | | ***** | **-** | | **-** | **-** | **-** | **6** |
| Anal HPV DNA | **-*** | ***** | | | **-** | | ***** | | **-** | | ***** | | ***** | | ***** | | **-** | | ***** | ***** | | ***** | **-** | | **-** | **-** | **-** | **8** |
| **C) Single arm trials - without any control (N_s_= 3)** | | | | | | | | | | | | | | | | | | | | | | | | | | | | |
| **Trial: NCT00604175** | | | | | | | | | | | | | | | | | | | | | | | | | | | | |
| Cespedes 2018:  Anal and Cervical DNA | ***-** | ***** | | | ***** | | ***** | | **-** | | ***** | | ***** | | ***** | | **-** | | ***** | ***** | | ***** | **-** | | **-** | **-** | ***** | **11** |
| **Trial: NCT00513526** | | | | | | | | | | | | | | | | | | | | | | | | | | | | |
| Wilkin 2010:  Anal DNA | ***-** | ***** | | | ***** | | ***** | | **-** | | ***** | | ***** | | ***** | | **-** | | ***** | ***** | | ***** | **-** | | **-** | **-** | ***** | **11** |
| Anal cytology | ***-** | ***** | | | ***** | | ***** | | **-** | | ***** | | ***** | | **-** | | **-** | | ***** | **-** | | **-** | **-** | | **-** | **-** | ***** | **8** |
| **Trial: NCT00667563** | | | | | | | | | | | | | | | | | | | | | | | | | | | | |
| Palefsky 2021:  Cervical HPV DNA | ***-** | ***** | | | ***** | | ***** | | **-** | | **-** | | ***** | | ***** | | **-** | | ***** | ***** | | ***** | **-** | | **-** | **-** | ***** | **10** |
| **Range of quality score for Randomized controlled trials (N_s_= 3)** | | | | | | | | | | | | | | | | | | | | | | | | | | |  | **8-14** |
| **Range of quality score for Single arm trials – with historical controls (N_s_= 2)** | | | | | | | | | | | | | | | | | | | | | | | | | | |  | **6-10** |
| **Range of quality score for Single arm trials – with historical controls (N_s_= 2)** | | | | | | | | | | | | | | | | | | | | | | | | | | |  | **8-11** |

# Supplement f**igure S1A-D:** Seropositivity for HPV in HIV positive populations that were not seronegative (e.g. seropositive or mixed populations) for the specific HPV type at baseline. Timing indicates the number of weeks since receiving the 1^st^ dose in the vaccination schedule. Locations: POL = Poland, SA = South Africa, USA = United States of America. Vaccine: bHPV = bivalent HPV vaccine, qHPV = quadrivalent HPV vaccine. ** indicates which results were included in the pooled estimate, ** indicates which results would have been pooled if enough estimates (n>1) were available*

#
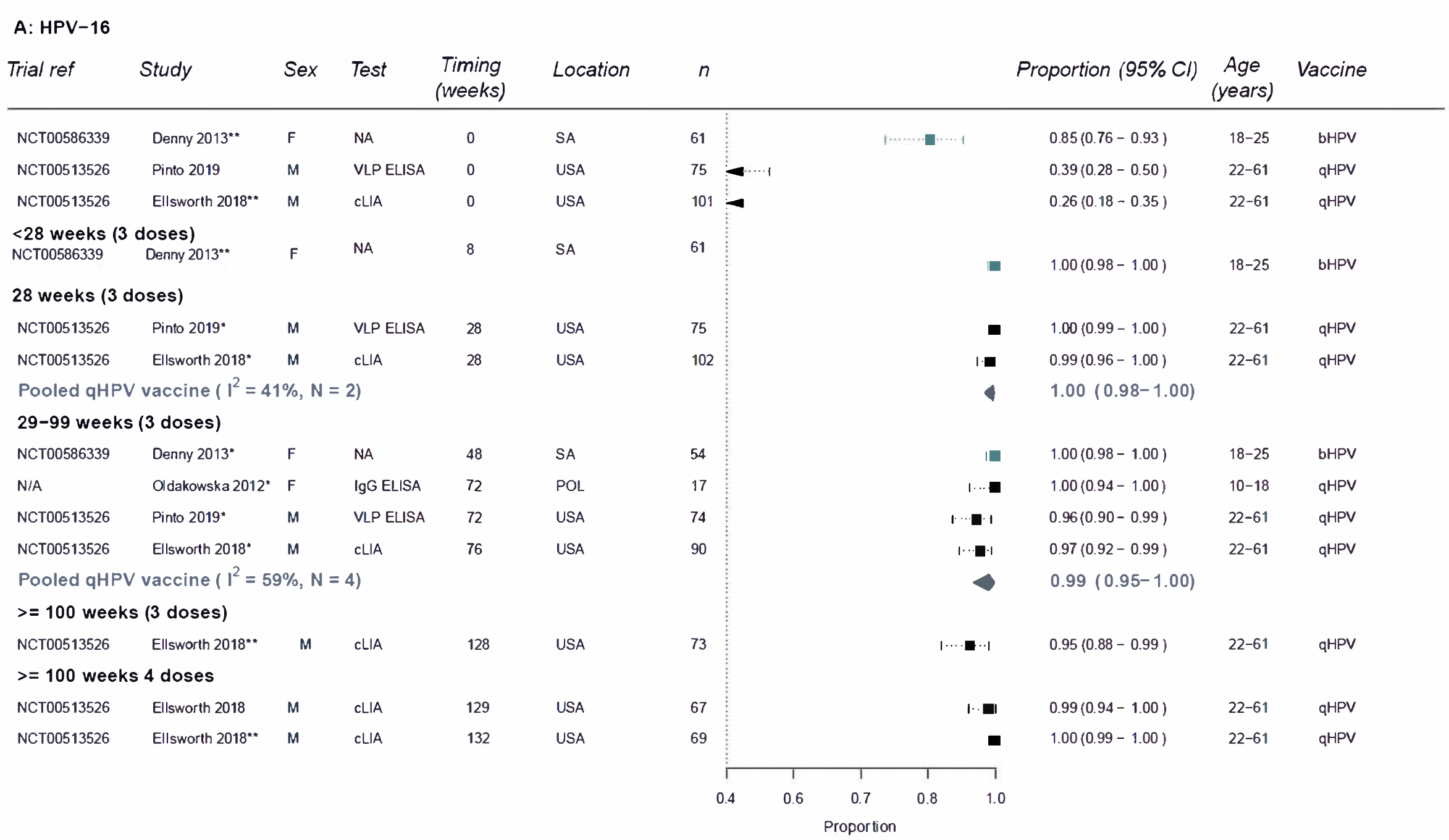


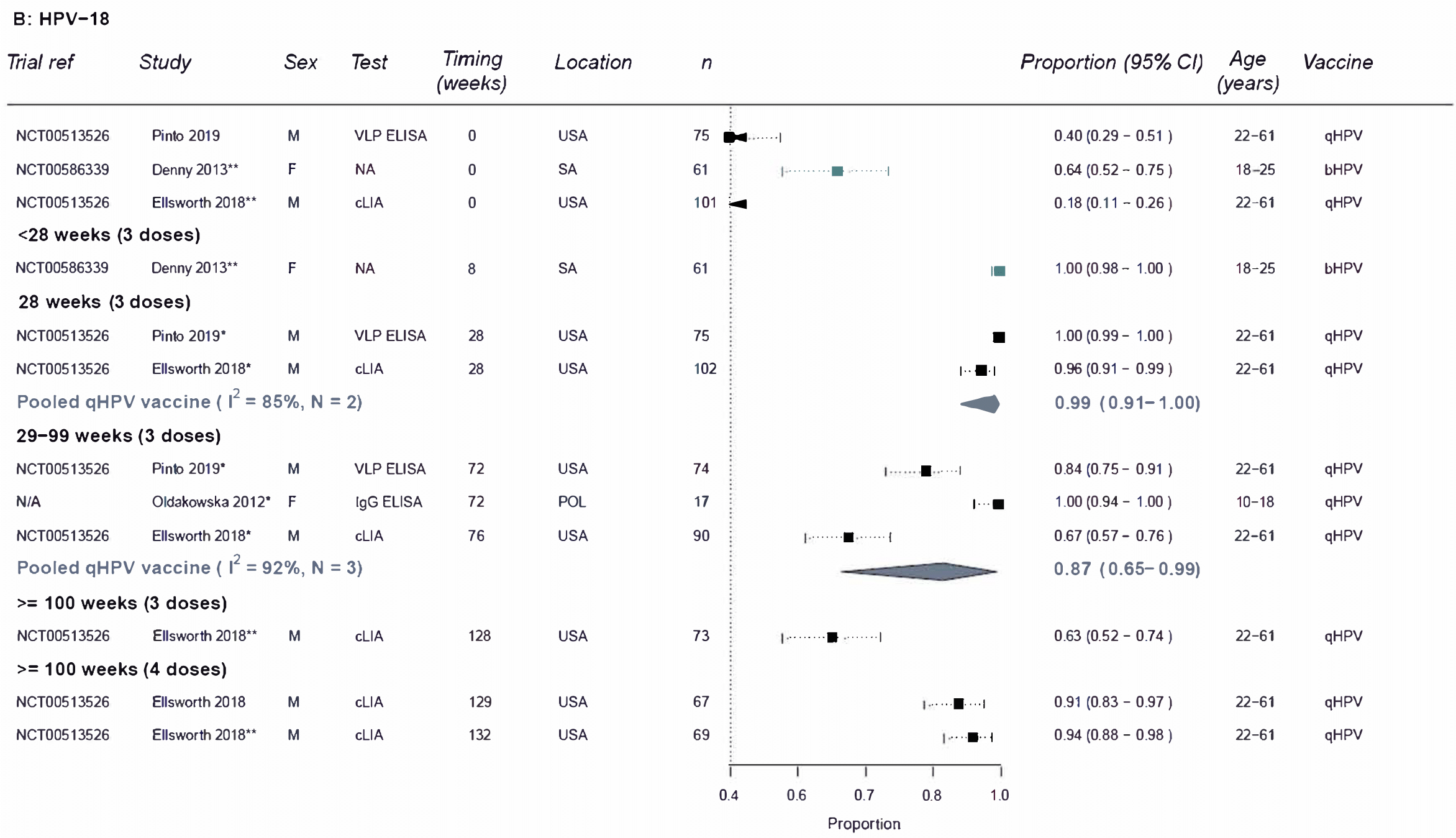

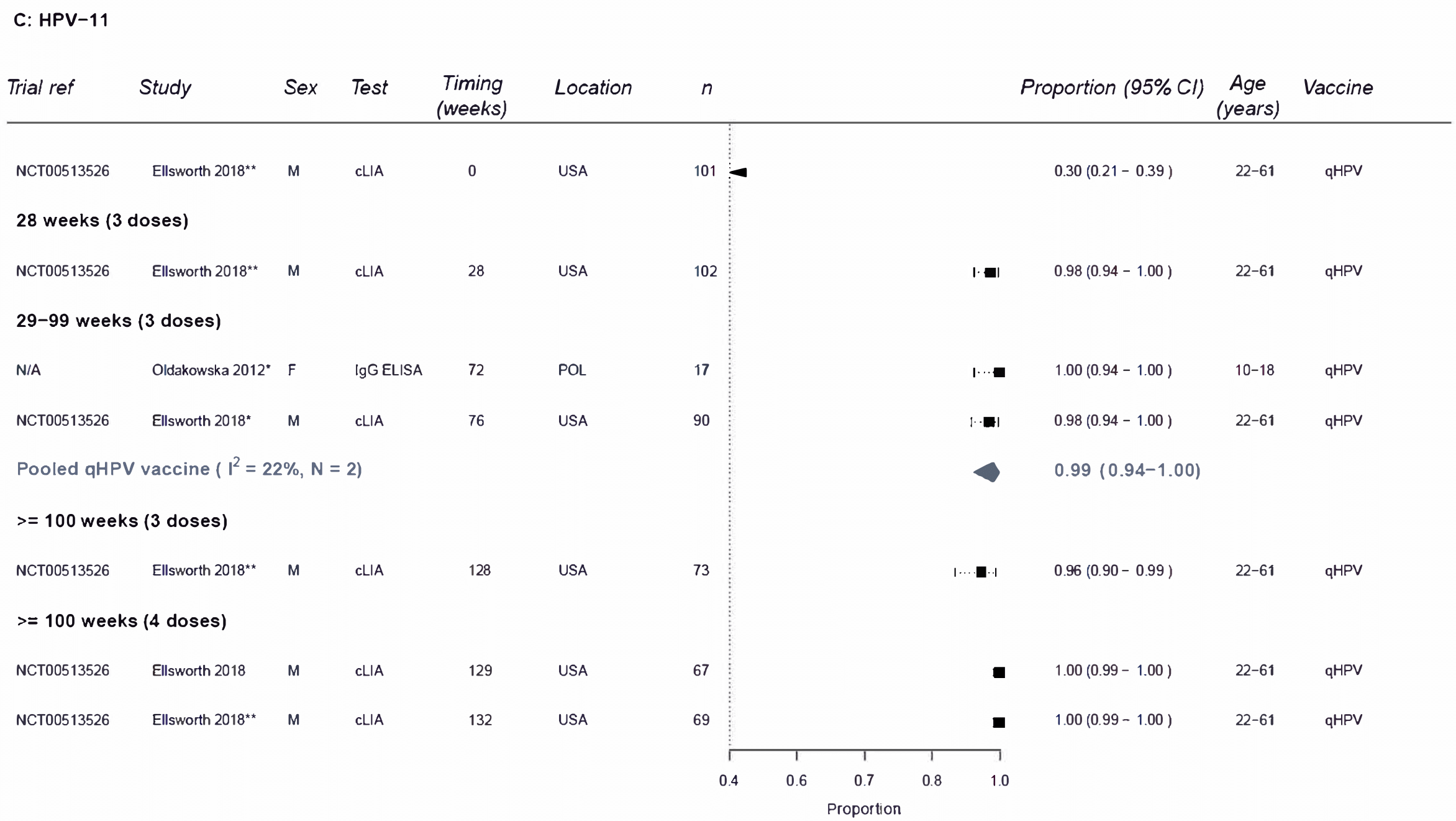

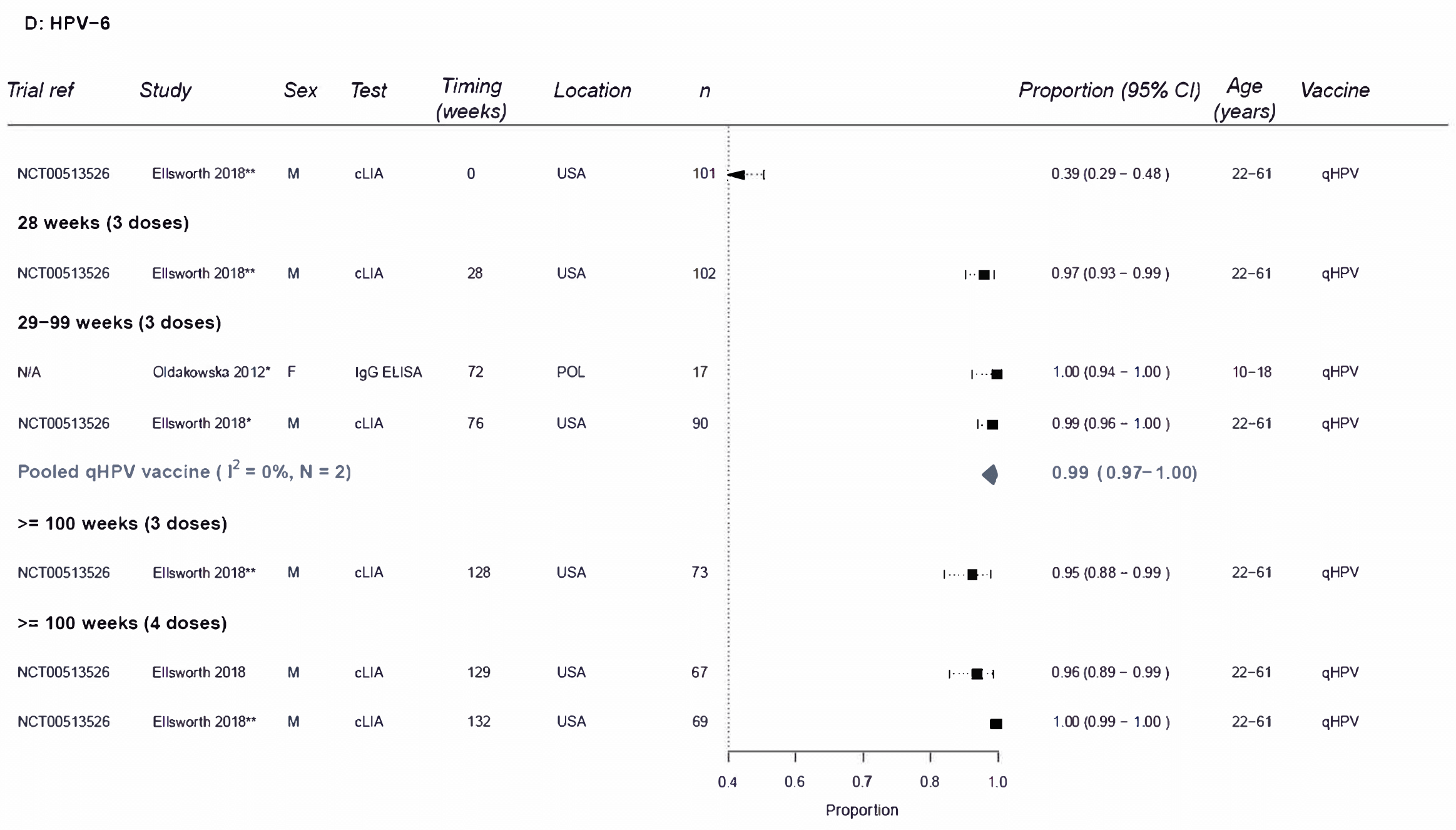


**Supplement figure S2A-D: GMT results following vaccination with the bivalent, quadrivalent or nonavalent vaccine for each individual study available for A) HPV 18 B) HPV-16, C) HPV-6, D) HPV-11.** Arrows indicate approximate timing of vaccination. Dotted line indicates cut-off value for seropositivity for the cLIA assay.


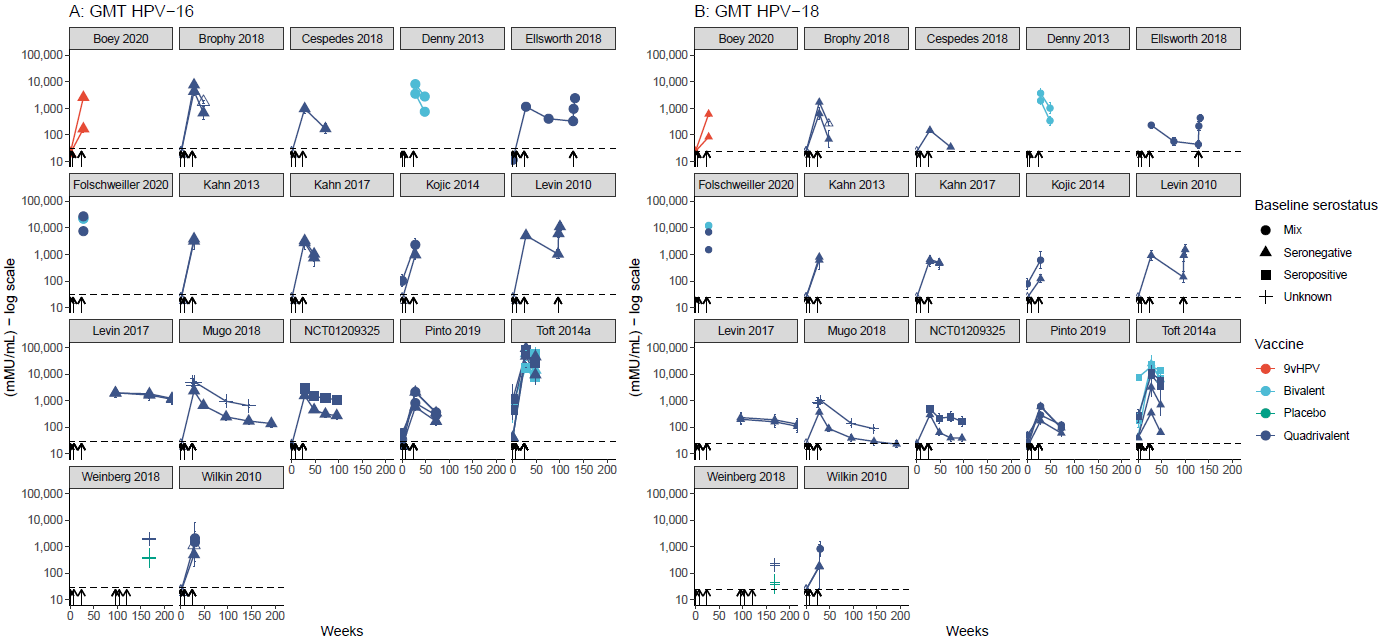


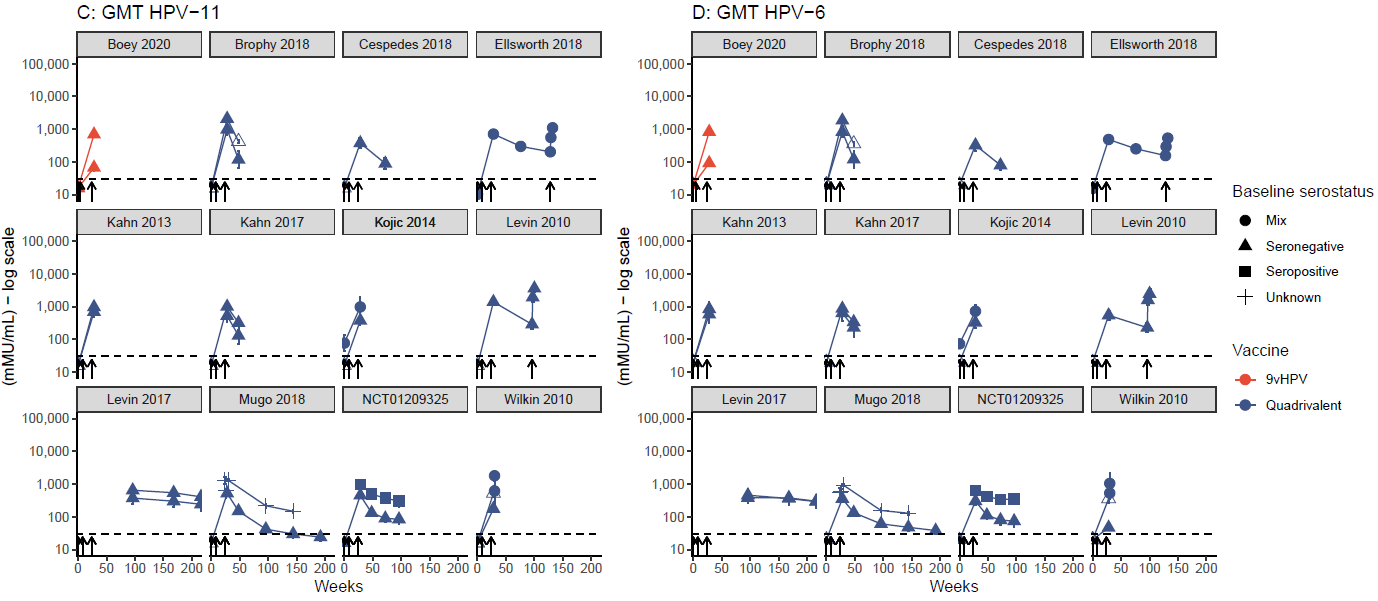


**Supplement figure S3: Geometric mean antibody titers over time for the bivalent vaccine in available studies (N_s_=3)**^1,2,29^**.** Arrows indicate approximate timing of vaccination for each study. Dotted line indicates cut-off value for seropositivity for the cLIA assay. N_s_ is the number of independent trials from which the data was taken.


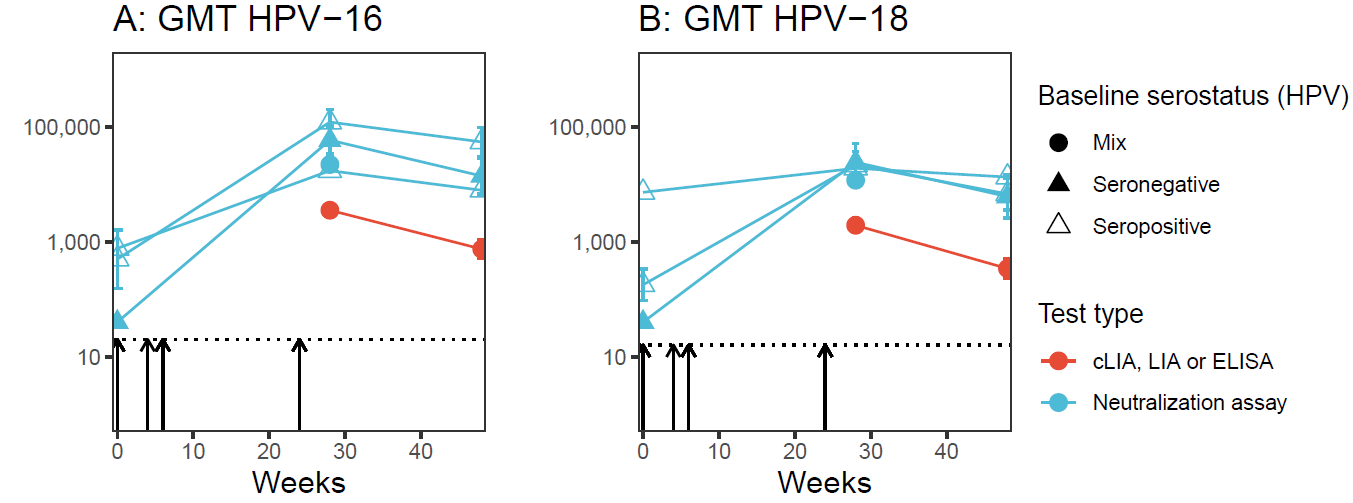


**Supplement figure S4: GMT titers– comparing the bivalent and quadrivalent vaccine (N_s_ = 1)**^29^**.** Arrows indicate approximate timing of vaccination. Neutralization assay was used – no cutoff for seropositivity was reported. N_p_ is the number of publications from which the data was taken.


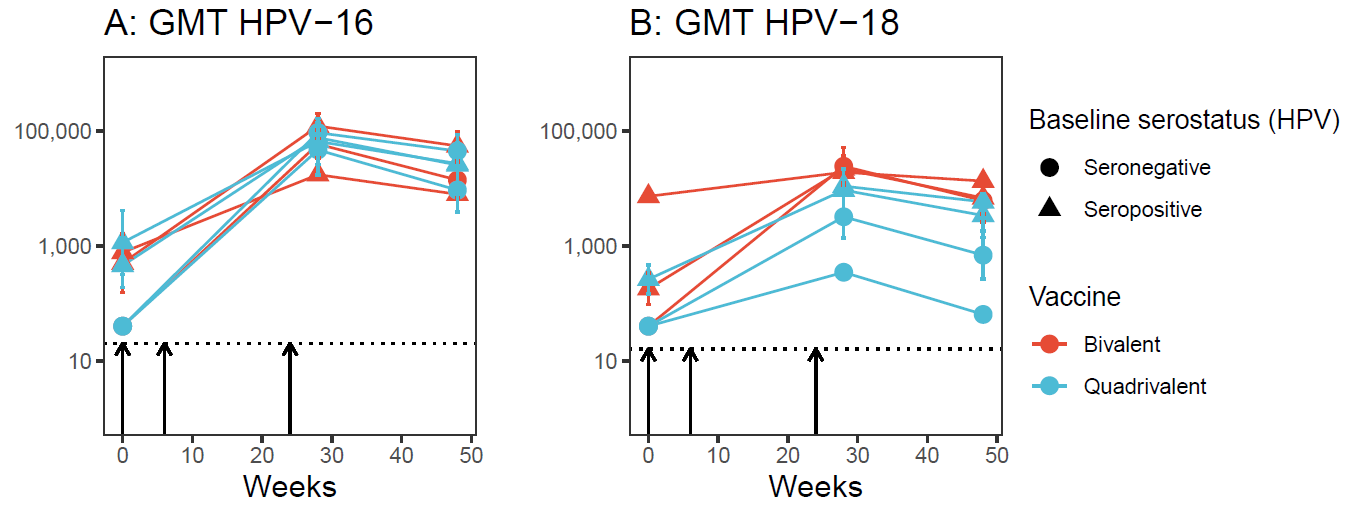


**Supplement Figure S5: Seropositivity results for HPV in HIV positive or negative populations receiving 3 doses of the quadrivalent HPV vaccine who were either baseline HPV negative or positive^a^.** Vaccines are colour-coded{ qHPV (black) or bHPV (dark green). Asterisk (*) indicates which results were included in the pooled estimate, ** indicates which results would have been pooled if enough estimates (n>1) were available. Sex are both (B), female (F) or male (M). Timing indicates the number of weeks since receiving the 1st dose in the vaccination schedule. Bivalent results added for completion; not added in pooled estimate. Locations; BR = Brazil, CAN = Canada, Den = Denmark, IND = India, KEN = Kenya, PR = Puerto Rico, SA = South Africa, USA = United States of America.


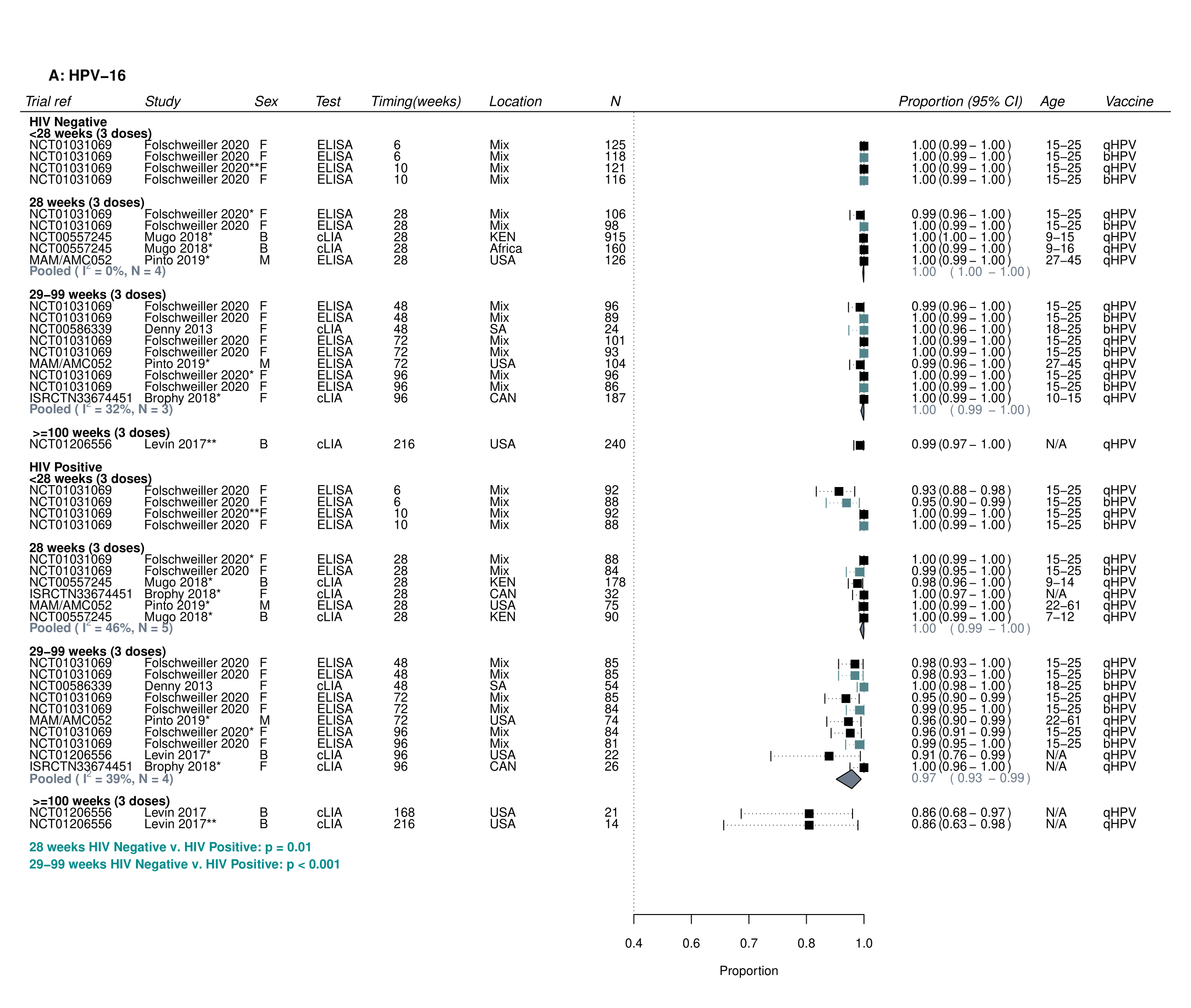


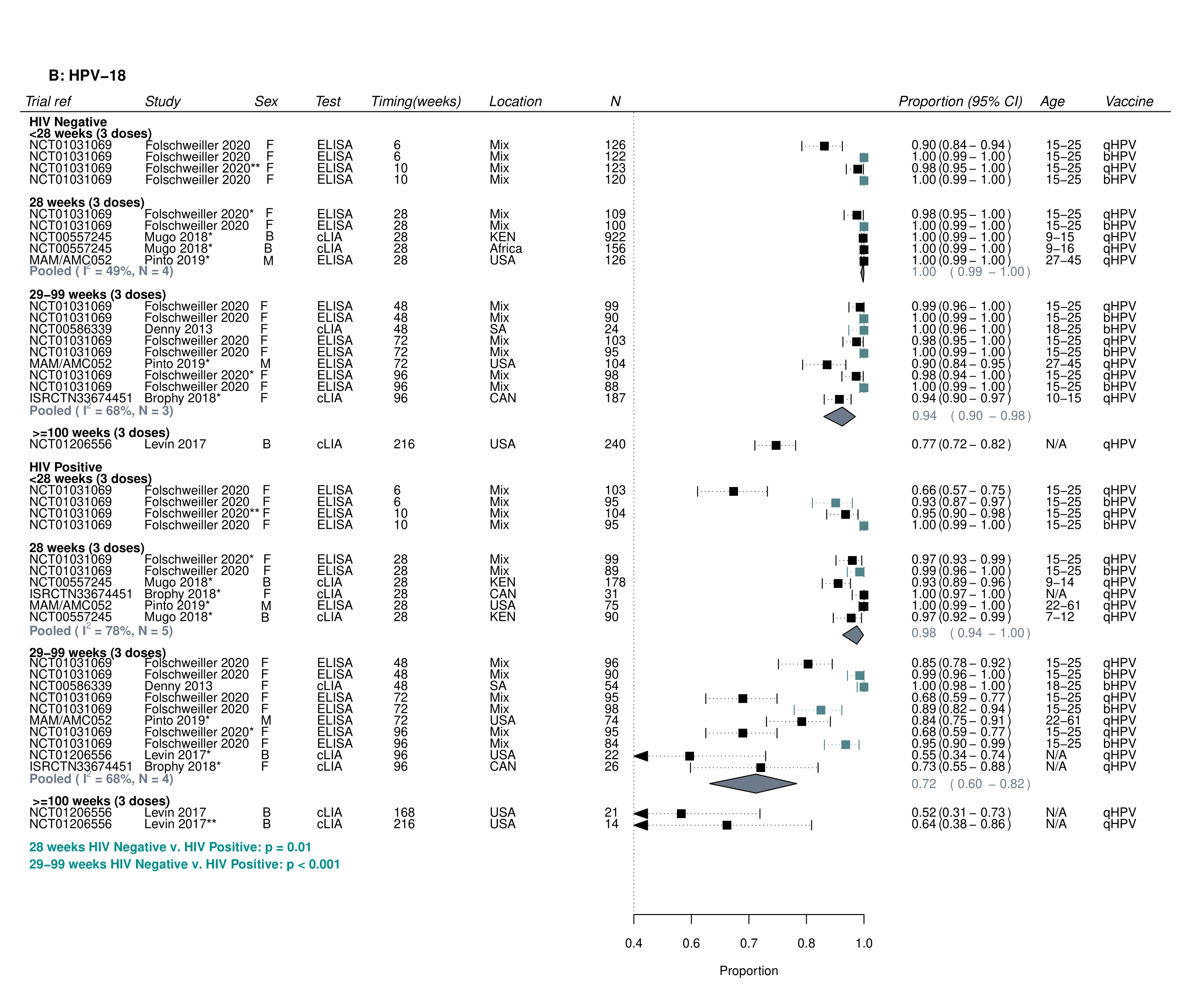


*
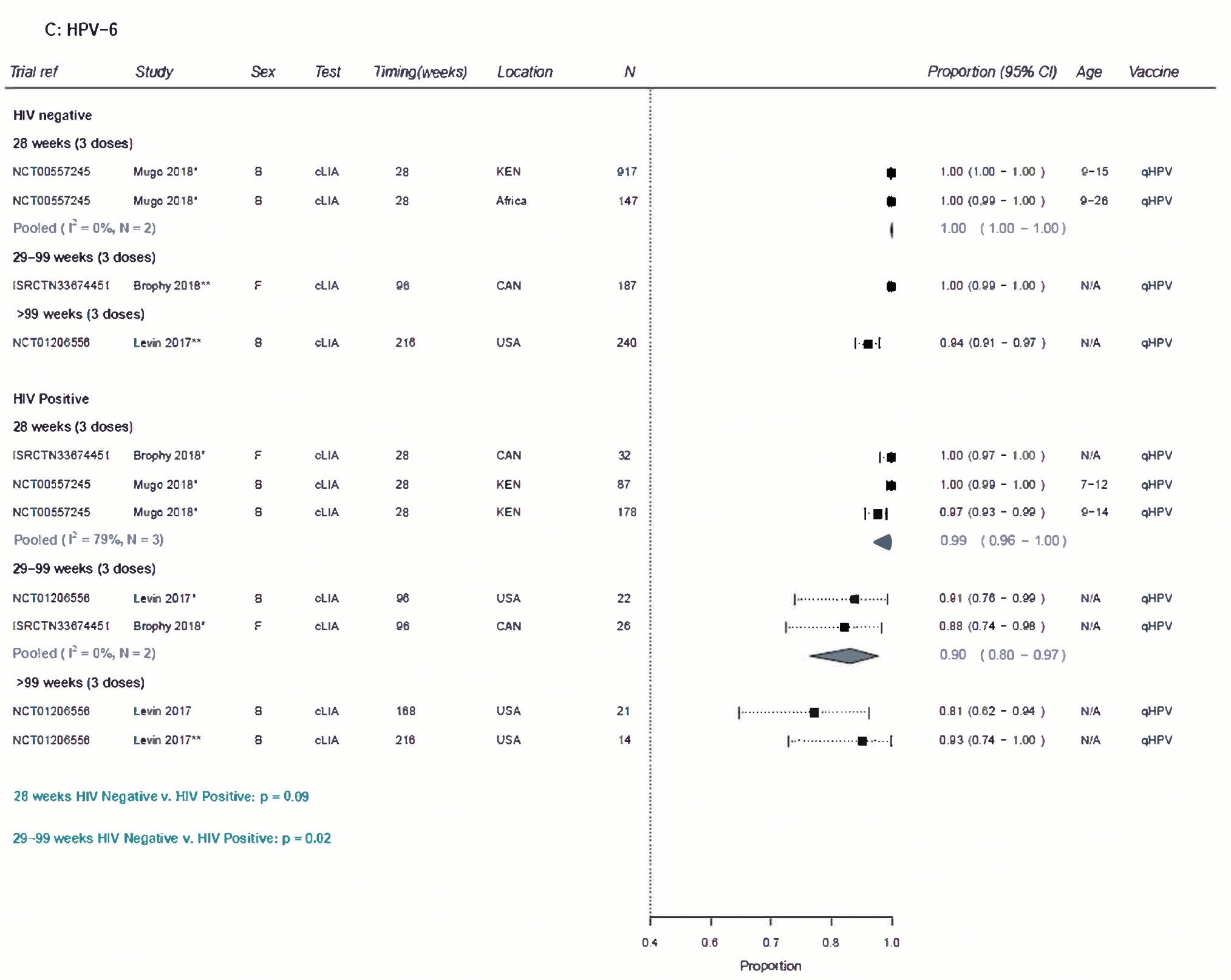

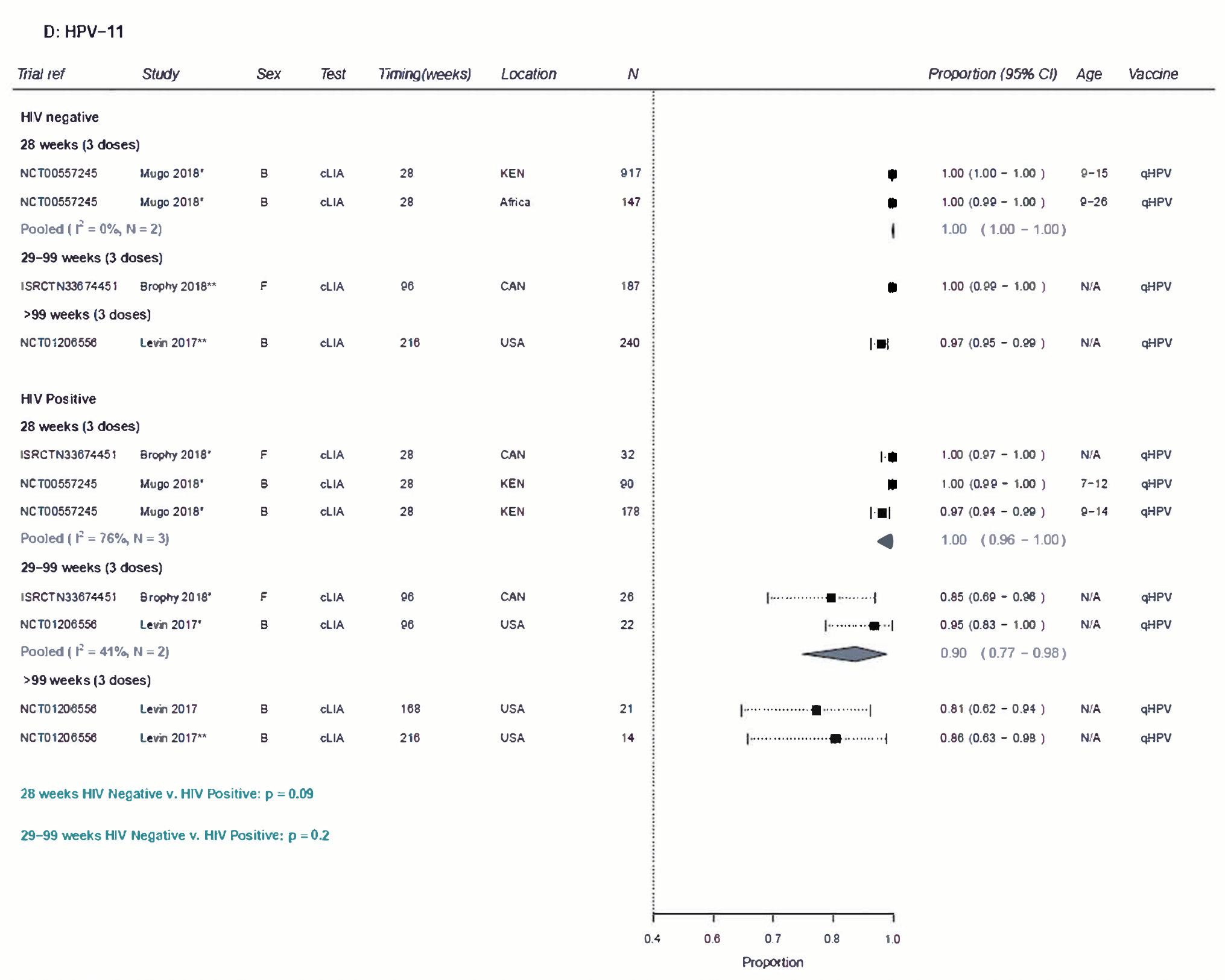
*

1. Results for Pinto 2019 and Denny 2013 are in a mixed population, the rest of the results are in a seronegative population.

***Supplement figure S6A-D: GMT titers stratified by HIV stages and ART.*** Results stratified by CD4 count were taken from Cespedes 2018, results stratified by CD4% (nadir) were taken from Levin 2010, results stratified by ART status were taken from Kahn 2017, results stratified by viral suppression (VL <50 copies/mL) were taken from Brophy 2018.


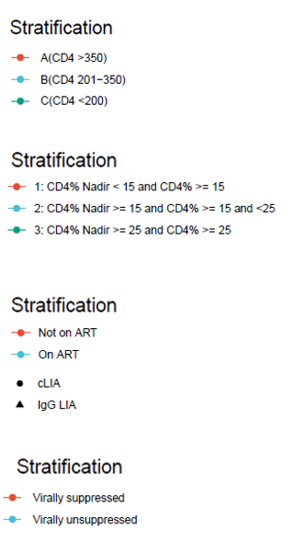

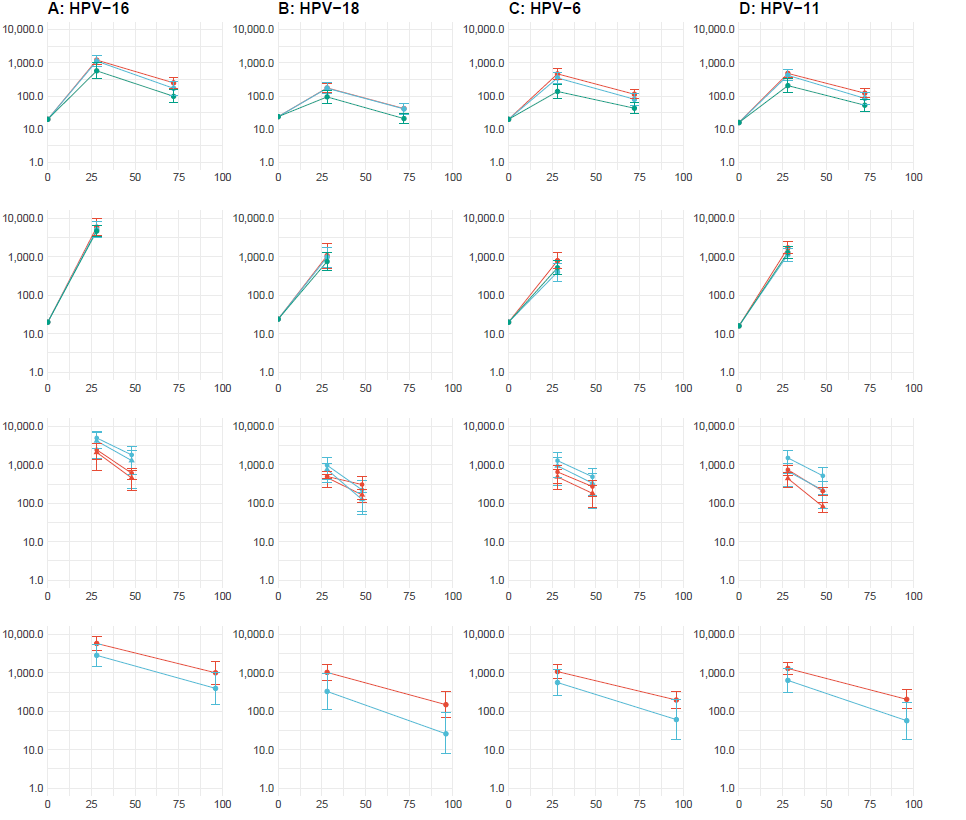


**Figure 7 A-D: Geometric antibody means titre (GMT) for A) HPV-16, B) HPV-18, C) HPV-6, and D) HPV-11 after administration of the quadrivalent, bivalent or nonavalent vaccine in PLHIV and HIV-negative individuals with mixed HPV serostatus at baseline (N_s_= 7 trials).** Arrows indicate timing of vaccine doses. Dotted line indicates cut-off value for seropositivity for the cLIA assay. Participants received the quadrivalent vaccine otherwise the panel heading indicates; * = bivalent vaccine, ** = nonavalent vaccine. SOT: solid organ transplant comparison group, HC: indicates when HIV negative participants were a historical control. Trials were only shown below if they contained both an arm (or HC) with PLHIV and HIV-negative individuals.


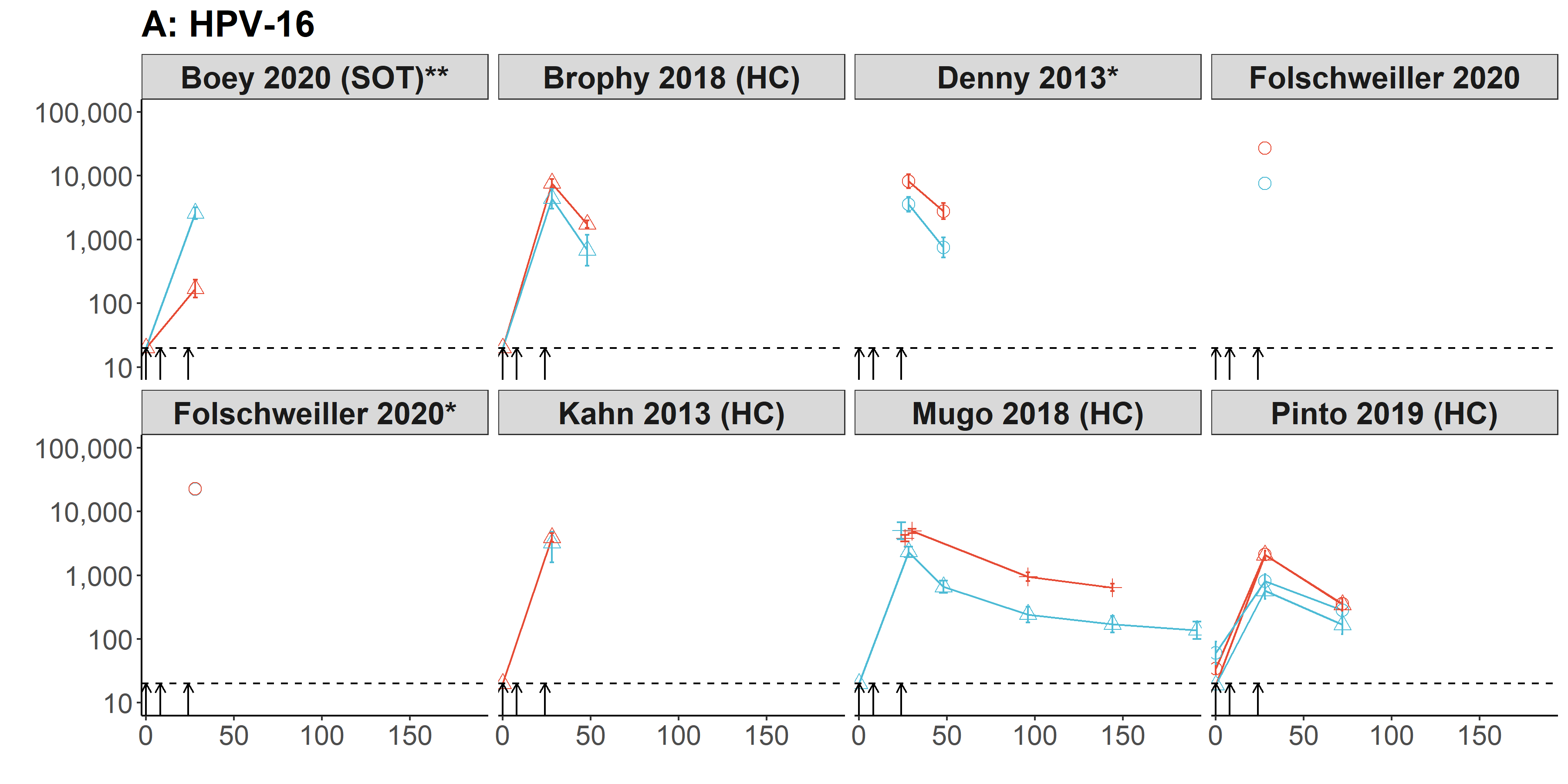


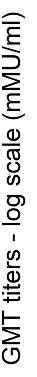


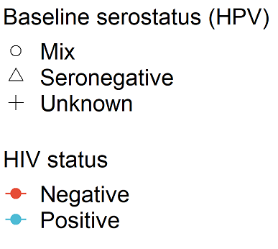


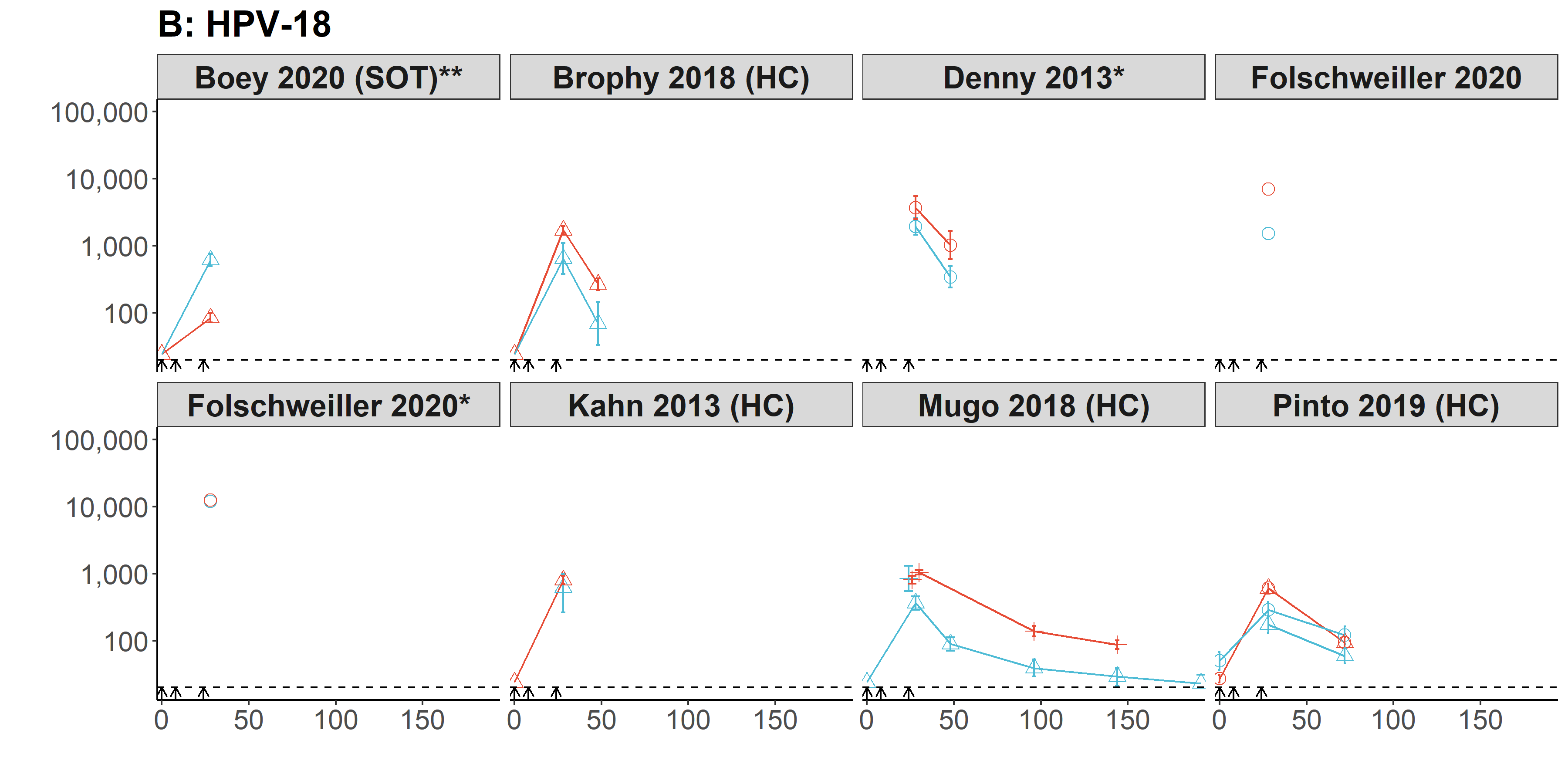


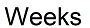


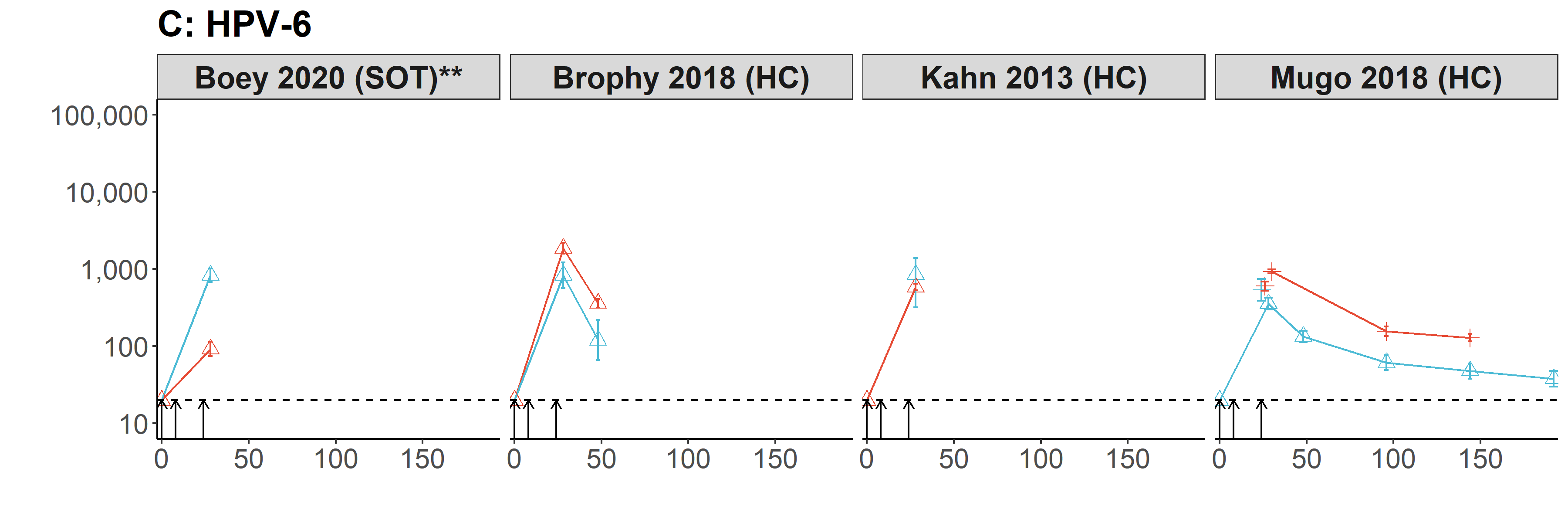


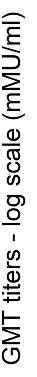


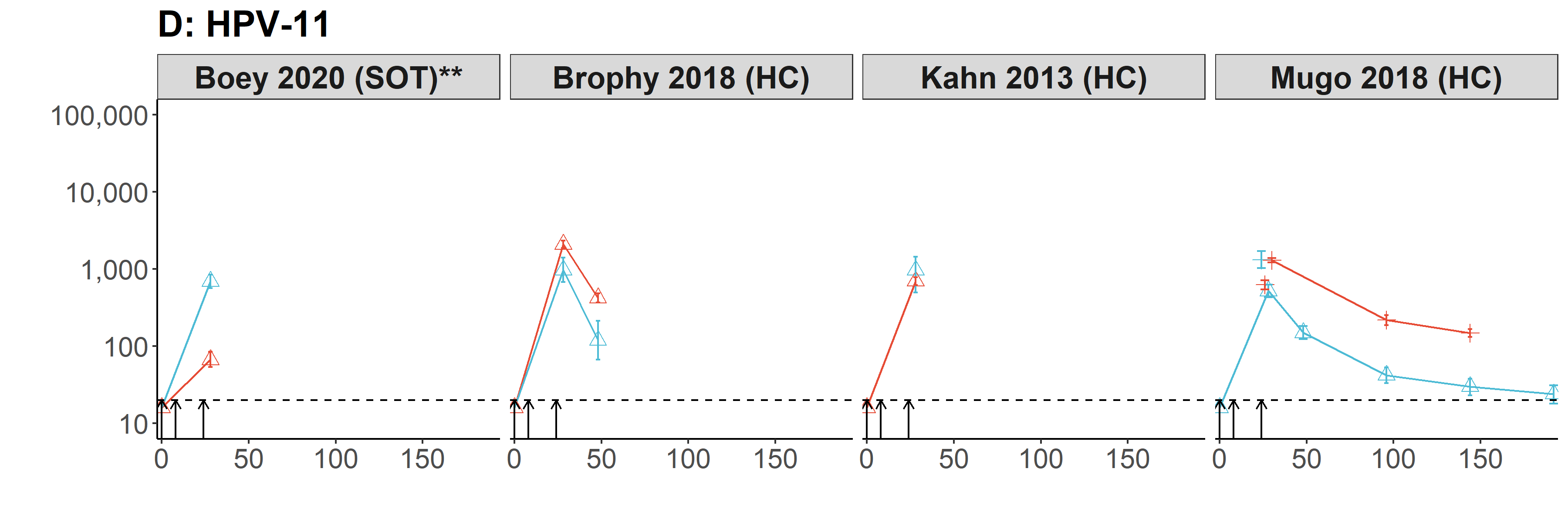


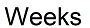


**Bibliography**

1. Denny, L. *et al.* Safety and immunogenicity of the HPV-16/18 AS04-adjuvanted vaccine in HIV-positive women in South Africa: A partially-blind randomised placebo-controlled study. *Vaccine* **31**, 5745–5753 (2013).

2. Folschweiller, N. *et al.* Immunogenicity and safety of the AS04-HPV-16/18 and HPV-6/11/16/18 human papillomavirus vaccines in asymptomatic young women living with HIV aged 15–25 years: A phase IV randomized comparative study. *EClinicalMedicine* **23**, (2020).

3. ClinicalTrials.gov. Evaluation of Safety and Immunogenicity of a Human Papillomavirus (HPV) Vaccine in Human Immunodeficiency Virus (HIV) Infected Females. https://clinicaltrials.gov/ct2/show/NCT01031069.

4. ClinicalTrials.gov. Safety and Immunogenicity of GlaxoSmithKline Biologicals’ HPV Vaccine 580299 (Cervarix) in HIV Infected Females. https://clinicaltrials.gov/ct2/show/NCT00586339.

5. Weinberg, A. *et al.* Humoral, mucosal, and cell-mediated immunity against vaccine and nonvaccine genotypes after administration of quadrivalent human papillomavirus vaccine to HIV-infected children. *J. Infect. Dis.* **206**, 1309–18 (2012).

6. Levin, M. J. *et al.* Safety and immunogenicity of a quadrivalent human papillomavirus (types 6, 11, 16, and 18) vaccine in HIV-infected children 7 to 12 years old. *J. Acquir. Immune Defic. Syndr.* **55**, 197–204 (2010).

7. ClinicalTrials.gov. Safety of and Immune Response to a Novel Human Papillomavirus Vaccine in HIV Infected Children. https://www.clinicaltrials.gov/ct2/show/NCT00339040.

8. Weinberg, A., Huang, S., Moscicki, A. B., Saah, A. & Levin, M. J. Persistence of memory B-cell and T-cell responses to the quadrivalent HPV vaccine in HIV-infected children. *AIDS* **32**, 851–859 (2018).

9. Levin, M. J. *et al.* Four-year persistence of type-specific immunity after quadrivalent human papillomavirus vaccination in HIV-infected children: Effect of a fourth dose of vaccine. *Vaccine* **35**, 1712–1720 (2017).

10. Wilkin, T. J. *et al.* A Randomized, Placebo-Controlled Trial of the Quadrivalent Human Papillomavirus Vaccine in Human Immunodeficiency Virus-Infected Adults Aged 27 Years or Older: AIDS Clinical Trials Group Protocol A5298. *Clin. Infect. Dis.* **67**, 1339–1346 (2018).

11. ClinicalTrials.gov. Quadrivalent HPV Vaccine to Prevent Anal HPV in HIV-infected Men and Women. https://clinicaltrials.gov/ct2/show/study/NCT01461096.

12. Fontes, A. *et al.* High specific immune response to a bivalent anti-HPV vaccine in HIV-1-infected men in São Paulo, Brazil. *Papillomavirus Res.* **2**, 17–20 (2016).

13. ClinicalTrials.gov. Vaccine Therapy in Preventing Human Papillomavirus Infection in Young HIV-Positive Male Patients Who Have Sex With Males. https://clinicaltrials.gov/ct2/show/NCT01209325.

14. McClymont, E. *et al.* Brief Report: Persistence of Non-Vaccine Oncogenic HPV Genotypes in Quadrivalent HPV-Vaccinated Women Living With HIV. *J. Acquir. Immune Defic. Syndr.* **83**, 230–234 (2020).

15. McClymont, E. *et al.* Impact of quadrivalent HPV vaccine dose spacing on immunologic response in women living with HIV. *Vaccine* **38**, 3073–3078 (2020).

16. McClymont, E. *et al.* The Efficacy of the Quadrivalent Human Papillomavirus Vaccine in Girls and Women Living With Human Immunodeficiency Virus. *Clin. Infect. Dis.* **68**, 788–794 (2019).

17. Brophy, J. *et al.* Immunogenicity and safety of the quadrivalent human papillomavirus vaccine in girls living with HIV. *Pediatr. Infect. Dis. J.* **37**, 595–597 (2018).

18. Money, D. M. *et al.* HIV viral suppression results in higher antibody responses in HIV-positive women vaccinated with the quadrivalent human papillomavirus vaccine. *Vaccine* **34**, 4799–4806 (2016).

19. ISRCTN registry. ISRCTN33674451: Human papillomavirus (HPV) vaccine in human immunodeficiency virus (HIV) positive girls and women. http://www.isrctn.com/ISRCTN33674451?q=cancer -radiotherapy&filters=conditionCategory:Infections and Infestations,trialStatus:Completed,recruitmentCountry:Canada&sort=&offset=3&totalResults=5&page=1&pageSize=10&searchType=basic-search.

20. Pinto, L. A. *et al.* Oral and systemic HPV antibody kinetics post-vaccination among HIV-positive and HIV-negative men. *Vaccine* **37**, 2502–2510 (2019).

21. Ellsworth, G. B. *et al.* A delayed dose of quadrivalent human papillomavirus vaccine demonstrates immune memory in HIV-1-infected men. *Papillomavirus Res.* **6**, 11–14 (2018).

22. Wilkin, T. *et al.* Safety and Immunogenicity of the Quadrivalent Human Papillomavirus Vaccine in HIV‐1–Infected Men. *J. Infect. Dis.* **202**, 1246–1253 (2010).

23. ClinicalTrials.gov. Human Papillomavirus Vaccine Therapy in Treating Men With HIV-1 Infection. https://clinicaltrials.gov/ct2/show/NCT00513526.

24. Kahn, J. A. *et al.* Immunogenicity and Safety of the Human Papillomavirus 6, 11, 16,18 Vaccine in HIV- Infected Young Women. *Clin. Infect. Dis.* **57**, 735–744 (2013).

25. Kahn, J. A., Xu, J., Kapogiannis, B. G. & Sleasman, J. W. Brief Report: Antibody Responses to Quadrivalent HPV Vaccination in HIV-Infected Young Women as Measured by Total IgG and Competitive Luminex Immunoassay. *J. Acquir. Immune Defic. Syndr.* **75**, 241–245 (2017).

26. ClinicalTrials.gov. Impact of a Human Papilloma Virus (HPV) Vaccine in HIV-Infected Young Women. https://clinicaltrials.gov/ct2/show/NCT00710593.

27. Zurek Munk-Madsen, M. *et al.* Cellular immunogenicity of human papillomavirus vaccines Cervarix and Gardasil in adults with HIV infection. *Hum. Vaccines Immunother.* **14**, 909–916 (2018).

28. Faust, H. *et al.* Human Papillomavirus neutralizing and cross-reactive antibodies induced in HIV-positive subjects after vaccination with quadrivalent and bivalent HPV vaccines. *Vaccine* **34**, 1559–1565 (2016).

29. Toft, L. *et al.* Comparison of the immunogenicity and reactogenicity of Cervarix and Gardasil human papillomavirus vaccines in HIV-infected adults: a randomized, double-blind clinical trial. *J. Infect. Dis.* **209**, 1165–73 (2014).

30. Toft, L. *et al.* Comparison of the immunogenicity of Cervarix® and Gardasil® human papillomavirus vaccines for oncogenic non-vaccine serotypes HPV-31, HPV-33, and HPV-45 in HIV-infected adults. *Hum. Vaccines Immunother.* **10**, 1147–1154 (2014).

31. Hidalgo-Tenorio, C. *et al.* Effectiveness of the Quadrivalent HPV Vaccine in Preventing Anal ≥ HSILs in a Spanish Population of HIV+ MSM Aged > 26 Years. *Viruses* **13**, (2021).

32. Hidalgo-Tenorio, C. *et al.* Safety and immunogenicity of the quadrivalent human papillomavirus (qHPV) vaccine in HIV-positive Spanish men who have sex with men (MSM). *AIDS Res. Ther.* **14**, 34 (2017).

33. Giacomet, V. *et al.* Safety and immunogenicity of a quadrivalent human papillomavirus vaccine in HIV-infected and HIV-negative adolescents and young adults. *Vaccine* **32**, 5657–5661 (2014).

34. Boey, L. *et al.* Immunogenicity and Safety of the 9-Valent Human Papillomavirus Vaccine in Solid Organ Transplant Recipients and Adults Infected With Human Immunodeficiency Virus (HIV). *Clin. Infect. Dis.* **73**, e661–e671 (2020).

35. ClinicalTrials.gov. Study of Safety, Tolerability and Immunogenicity of Gardasil®9 in Immunocompromised Patients. https://clinicaltrials.gov/ct2/show/NCT03525210.

36. Ołdakowska, A. *et al.* [Evaluation of antibody response to HPV vaccination in HIV infected girls]. *Przegl. Epidemiol.* **66**, 651–5 (2012).

37. Denny, L. *et al.* Safety and immunogenicity of the HPV-16/18 AS04-adjuvanted vaccine in HIV-positive women in South Africa: A partially-blind randomised placebo-controlled study. *Vaccine* **31**, 5745–5753 (2013).

38. Mugo, N. *et al.* Antibody responses to prophylactic quadrivalent human papillomavirus vaccine at 48 months among HIV-infected girls and boys ages 9–14 in Kenya, Africa. *Vaccine* **39**, 4751–4758 (2021).

39. Mugo, N. R. *et al.* Quadrivalent HPV vaccine in HIV-1-infected early adolescent girls and boys in Kenya: Month 7 and 12 post vaccine immunogenicity and correlation with immune status. *Vaccine* **36**, 7025–7032 (2018).

40. ClinicalTrials.gov. Evaluation of Safety and Immunogenicity of a Human Papillomavirus (HPV) Vaccine in Human Immunodeficiency Virus (HIV) Infected Females. https://clinicaltrials.gov/ct2/show/study/NCT01031069.

41. Palefsky, J. M. *et al.* Safety and Immunogenicity of the Quadrivalent vaccine in Indian Women Living with HIV. *JAIDS J. Acquir. Immune Defic. Syndr.* (2021) doi:10.1097/QAI.0000000000002657.

42. ClinicalTrials.gov. Vaccine Therapy in Preventing HPV in HIV-Positive Women in India. https://clinicaltrials.gov/ct2/show/NCT00667563.

43. Cespedes, M. S. *et al.* Anogenital human papillomavirus virus DNA and sustained response to the quadrivalent HPV vaccine in women living with HIV-1. *Papillomavirus Res.* **6**, 15–21 (2018).

44. Kojic, E. M. *et al.* Immunogenicity and safety of the quadrivalent human papillomavirus vaccine in HIV-1-infected women. *Clin. Infect. Dis.* **59**, 127–35 (2014).

45. ClinicalTrials.gov. Safety of and Immune Response to the Human Papillomavirus (HPV) Vaccine in HIV-Infected Women. https://clinicaltrials.gov/ct2/show/NCT00604175.

46. Toft, L., Tolstrup, M., Storgaard, M., Østergaard, L. & Søgaard, O. S. Vaccination against oncogenic human papillomavirus infection in HIV-infected populations: review of current status and future perspectives. *Sex. Health* **11**, (2014).

47. ClinicalTrials.gov Results Data Element Definitions for Interventional and Observational Studies. https://prsinfo.clinicaltrials.gov/results_definitions.html.
